# Highly luminescent palladium(ii) complexes with sub-millisecond blue to green phosphorescent excited states. Photocatalysis and highly efficient PSF-OLEDs†
†Electronic supplementary information (ESI) available. CCDC 1048773 and 1449190. For ESI and crystallographic data in CIF or other electronic format see DOI: 10.1039/c6sc00462h


**DOI:** 10.1039/c6sc00462h

**Published:** 2016-06-15

**Authors:** Pui-Keong Chow, Gang Cheng, Glenna So Ming Tong, Chensheng Ma, Wai-Ming Kwok, Wai-Hung Ang, Clive Yik-Sham Chung, Chen Yang, Feng Wang, Chi-Ming Che

**Affiliations:** a State Key Laboratory of Synthetic Chemistry , Institute of Molecular Functional Materials , HKU-CAS Joint Laboratory on New Materials and Department of Chemistry , The University of Hong Kong , Pokfulam Road , Hong Kong , China . Email: cmche@hku.hk; b HKU Shenzhen Institute of Research and Innovation , Shenzhen 518053 , China; c School of Chemistry and Chemical Engineering , Shenzhen University , Shenzhen 518060 , China; d Department of Applied Biology and Chemical Technology , The Hong Kong Polytechnic University , Hung Hom , Kowloon , Hong Kong SAR , China

## Abstract

Palladium(ii) complexes supported by tetradentate [N^C^C^N] and [O^N^C^N] ligand systems display sky blue to red phosphorescence with emission quantum yields and emission lifetimes up to 0.64 and 272 μs, respectively. Femtosecond time-resolved fluorescence (fs-TRF) measurements on these Pd(ii) complexes reveal a fast intersystem crossing from singlet to triplet manifolds with time constants of 0.6–21 ps. DFT/TDDFT calculations revealed that, as a result of the spiro-fluorene and bridging tertiary amine units of the ligands, the T_1_ excited state is more ligand-localized and has smaller structural distortion, leading to slower non-radiative decay as well as radiative decay of T_1_ → S_0_ transition and thereby highly emissive, long-lived triplet excited states. The Pd(ii) complexes have been found to be efficient catalysts for visible light-driven, reductive C–C bond formation from unactivated alkyl bromides with conversions and yields of up to 90% and 83%, respectively. These complexes have also been employed as photosensitizers for [2 + 2] cycloaddition of styrenes, with conversions and yields comparable to those of the reported Ir(iii) complexes. Both green and sky blue organic-light emitting devices (OLEDs) have been generated with these Pd(ii) complexes as guest emitters. Maximum external quantum efficiencies (EQE) of up to 16.5% have been achieved in the sky blue OLEDs. The long emission lifetimes render the Pd(ii) complexes good sensitizers for phosphor-sensitized fluorescent OLEDs (PSF-OLEDs). By utilizing these phosphorescent Pd(ii) complexes as sensitizers, highly efficient green and yellow PSF-OLEDs having high EQE (up to 14.3%), high colour purity and long operation lifetimes, with 90% of initial luminance (LT_90_) for more than 80 000 h, have been realized.

## Abstract 2

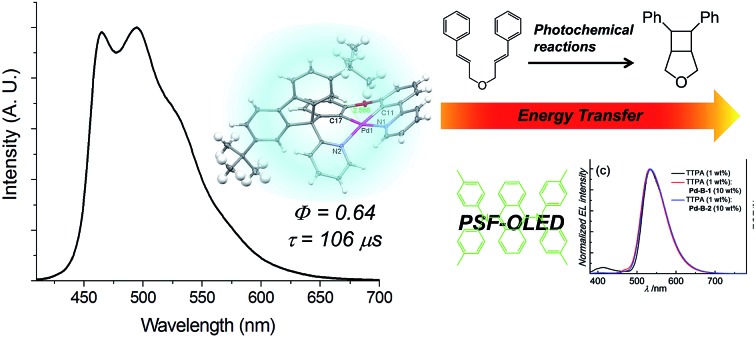
Pd(II) complexes with long-lived emissive excited states found applications in photo-catalysis and PSF-OLEDs.

## Introduction

Phosphorescent transition metal complexes having long-lived (100 μs range), high-energy triplet excited states (*E*_t_ ∼ 2.4–2.7 eV) accompanied by high emission quantum yields are highly desirable since they can be promising photo-catalysts in photochemical reactions,^1^ probes for bio-imaging and bio-molecule sensing,^2^ and sensitizers for energy conversion processes such as triplet–triplet annihilation (TTA) energy transfer.^3^ Most phosphorescent complexes of Ru(ii), Pt(ii) and Ir(iii) have emission lifetimes within 0.1–10 μs owing to their emissive excited states having ^3^MLCT (MLCT = metal-to-ligand charge transfer) character; the large spin–orbit coupling (SOC) constants of the heavy metal ion facilitates radiative decay (*k*_r_) from the lowest energy triplet excited state (T_1_) to the ground state (S_0_).^4^ Introducing naphthalenediimide, coumarin, Bodipy, anthracene or pyrene to the ligand system, or as ancilliary ligand of these metal complexes, was found to result in low-energy phosphorescence in the red to near-infrared spectral region (580–732 nm) with long emission lifetimes in the tens to hundreds of microseconds.^5^–^7^ This has been explained by the localization of emitting triplet excited states on the ligands [^3^ππ* or ^3^LLCT (LLCT = ligand-to-ligand charge transfer)]. Thus, the SOC effect is diminished and *k*_r_ is subsequently decreased. Our recent works showed that neutral complexes of Pd(ii) and Au(iii) exhibit green phosphorescence with long emission lifetimes in the 100 μs range and high emission quantum yields of up to 22% and 61%, respectively.^8^,^9^ The applications of these luminescent Pd(ii) and Au(iii) complexes in photocatalytic C–H functionalization, energy conversion by TTA, and OLEDs have also been examined. More recently, tris-cyclometalated Pt(iv) complexes, *fac*-[Pt(C^N)_3_]OTf (H–C^N = 2-phenylpyridine/1-phenylpyrazole), which emanate ligand-centred phosphorescence at 402–453 nm with emission lifetimes of up to 319 μs, have been reported.^10^

Few reports describe Pd(ii) complexes that display phosphorescence in the blue to green spectral region (*λ*_em_ ∼ 450–540 nm) at room temperature. A common perception is that the low lying d–d excited states of Pd(ii) complexes usually occur at an energy lower than that of the intraligand and/or MLCT excited state, thereby effectively quenching the emission of the complexes.^9^,^11^ Deliberate ligand design, such as introducing strong σ-donor atoms to the ligand system to destabilize the d–d state, or employing a multidentate ligand system to minimize excited state structural distortion, has been found to be effective in harvesting intense phosphorescence for most platinum group metal (PGM) complexes.^12^,^13^ Our recent work shows that Pd(ii) complexes supported by the tetradentate [O^N^C^N] ligands that have deprotonated carbon donor atom(s) exhibit intense green phosphorescence at room temperature with emission maxima (*λ*_em_) at 498–540 nm, high emission quantum yields (*Φ*_em_ up to 0.22) and long emission lifetimes (*τ*_obs_ = 62–122 μs).^9^ Detailed analysis by DFT/TDDFT calculations together with fs-time-resolved fluorescence (TRF) and fs/ns-transient absorption (TA) measurements suggest that structural distortions of the emissive triplet excited states of this class of complexes are small; this has been plausibly attributed to the rigid metal chelating ring system. The destabilization of the d–d excited state by the strong C donor atom also leads to less effective quenching of the emissions. The long phosphorescence lifetimes of these Pd(ii) complexes, being in the 100 μs range, are attributed to the emission derived from intraligand excited states with little metal parentage.

Long-lived excited states are desirable for photochemical reactions as they have sufficient time for bimolecular energy/electron transfer processes to take place. While the visible light-induced reductive C–C bond formation from alkyl halides and [2 + 2] styrene cycloaddition by energy transfer mechanism have been widely studied using *fac*-Ir(ppy)_3_, [Ir{dF(CF_3_)ppy}_2_(dtbbpy)]PF_6_ or Ru(bpy)_3_Cl_2_ as a sensitizer,1a,b,^14^ the utilization of other luminescent transition metal complexes for these photo-catalytic reactions has been less explored. This is especially true for Pd(ii) complexes, whose photochemistry is under-developed. While the severe efficiency roll-off caused by long emission lifetimes (up to 100 μs) would disfavour the utility of luminescent Pd(ii) complexes as phosphorescent dopant materials for OLEDs, the long emission lifetimes would be advantageous in phosphor-sensitized fluorescent OLEDs (PSF-OLEDs). The PSF-OLED works by using a phosphorescent sensitizer to excite a fluorescent dye.15a,b In brief, as the phosphor contains a heavy metal ion, all the high energy singlet excited states of the phosphor would rapidly decay to the lowest energy triplet excited state due to the spin–orbit coupling effect. This triplet excited state undergoes energy transfer to the radiative singlet manifold of a fluorescent dye *via* a Förster resonance energy transfer mechanism (FRET).15a,b This energy conversion has been shown to be an effective means for a fluorescent OLED to acquire a higher EL efficiency than its statistical limit of 25%.^15^ Luminescent Ir(iii) complexes have previously been used as phosphor sensitizers in most reported PSF-OLEDs, but the energy transfer from the Ir(iii)-phosphor to organic fluorophores is usually incomplete, and hence a significant residual phosphorescence is present. Incomplete energy transfer arises because the phosphorescence lifetimes of most reported luminescent Ir(iii) complexes are relatively short (usually below 10 μs).^16^ Since the radiative decay of the triplet excited state and energy transfer of phosphor to the organic fluorophore are two competitive processes, by using a phosphor with a long emission lifetime (say in the 100 μs range), complete energy transfer from the phosphor to the organic fluorophore becomes more likely. This is crucial to attain high efficiency PSF-OLEDs with good colour purity.

**Scheme 1:**
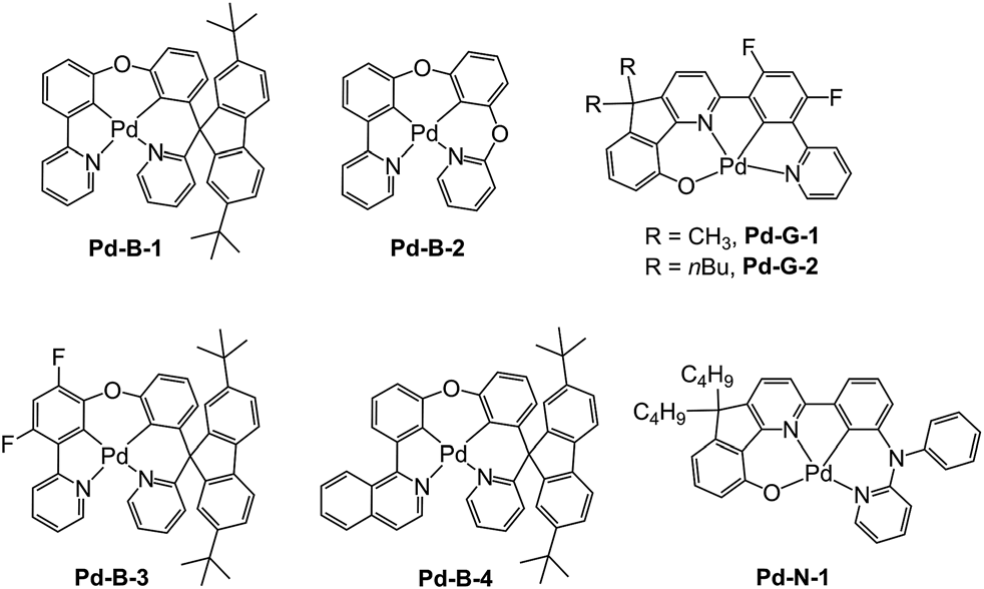
Chemical structures of the Pd(ii) complexes.

In the present work, we describe a panel of new luminescent Pd(ii) complexes (Scheme 1) which display phosphorescence in the blue to red spectral region (*λ*_em_ = 466–599 nm) and with long lifetimes of up to 272 μs (in CH_2_Cl_2_) as well as high *Φ*_em_ of up to 64% (in PMMA) at room temperature. High EQEs of 14.5% and 16.5% have been achieved for the green OLED with the **Pd-G-1** emitter and the sky blue OLED with the **Pd-B-1** emitter, respectively. By using these Pd(ii) complexes as sensitizers in the energy down conversion process, highly efficient PSF-OLEDs having EQEs of up to 14.3%, high colour purity, slow efficiency roll-offs, and long device operation lifetimes (LT_90_) of more than 80 000 h have been produced. Using these Pd(ii) complexes as photocatalysts, the visible light-driven reductive C–C bond formation of alkyl bromides has been investigated. Conversions and product yields of up to 90% and 83%, respectively, have been achieved. The [2 + 2] cycloaddition of styrenes, induced by energy transfer from the excited state of the Pd(ii) complexes, has been found to have substrate conversions and product yields of up to 100% and 97%, respectively.

## Results

### Design and synthesis of ligands and complexes

In previous study, we reported a series of Pd(ii) complexes supported by [O^N^C^N] ligands that show an intense green phosphorescence (*Φ*_em_ up to 22%) with a long *τ*_obs_ (up to 122 μs) at room temperature. These [Pd(O^N^C^N)] complexes were found to sensitize triplet–triplet annihilation (TTA) of 9,10-diphenylanthracene (DPA) (*Φ*_delayed fluorescence_ of up to 21%), catalyse photo-induced aerobic oxidation of secondary amines with up to 1650 turnovers in 2 h, and serve as a phosphorescent dopant in OLEDs with an EQE of up to 7.4%.^9^ As the ligand structure has a profound effect on the photophysical and photochemical properties of the metal complex, a new panel of tetradentate ligands have been prepared and used for preparing highly luminescent Pd(ii) complexes. **Pd-G-1** was designed by modification of the reported [Pd(O^N^C^N)] complexes by replacing the *n*-butyl with methyl groups.^9^ This modification is envisaged to lead to shorter intermolecular contact. **Pd-N-1** features a bridging tertiary amine between the phenyl and terminal pyridine groups of the [O^N^C^N] ligand resulting in a 6-5-6-membered metallocycle. Complexes **Pd-B-1**, **Pd-B-3** and **Pd-B-4** have a [N^C^C^N] ligand scaffold containing a spiro-fluorene group installed between a phenyl ring and a pyridine ring.

**Scheme 2:**
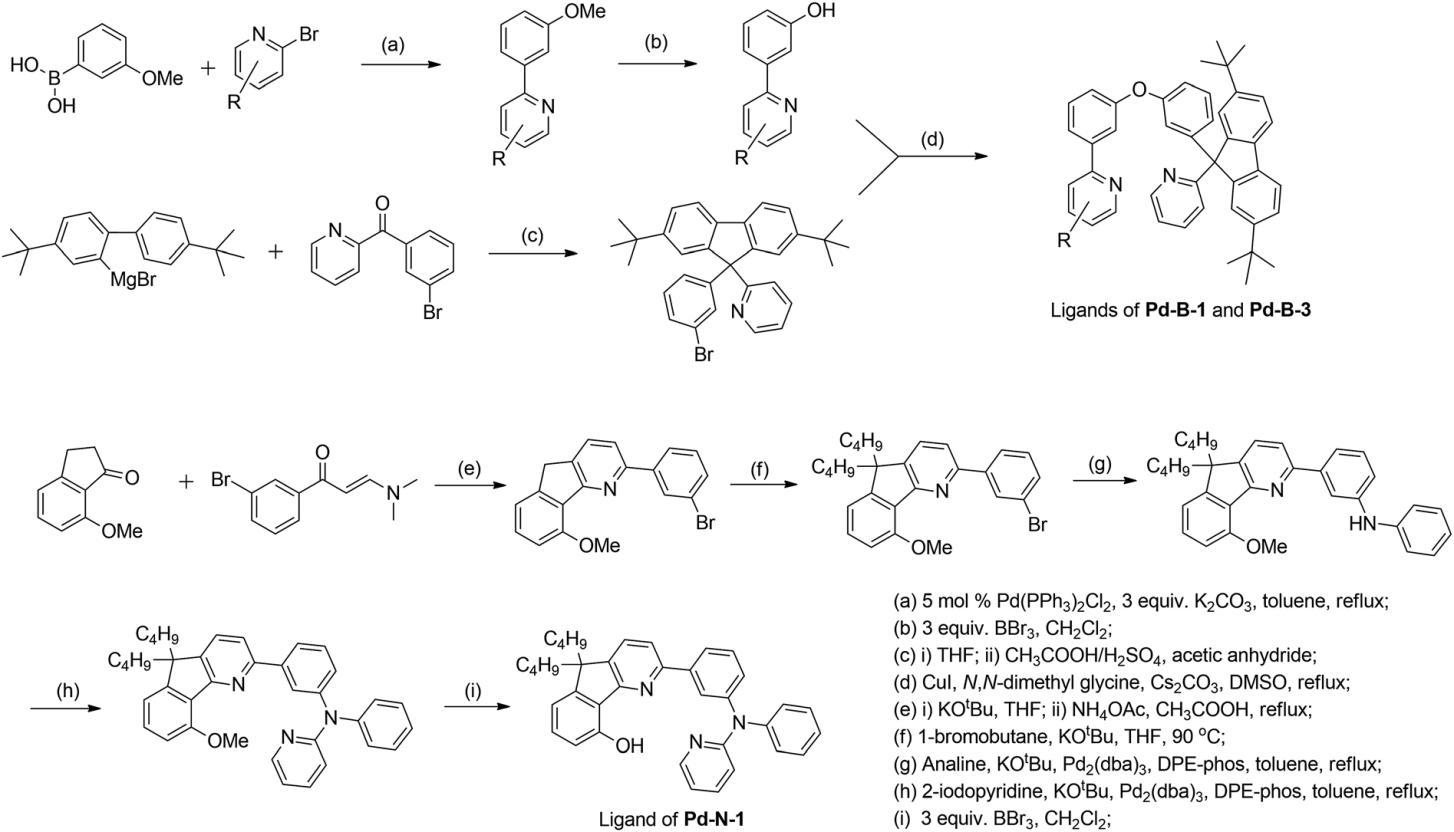
General synthetic scheme for the ligands of **Pd-B-1**, **Pd-B-3** and **Pd-N-1**.

The tetradentate ligands of complexes **Pd-N-1**, **Pd-B-1** and **Pd-B-3** were prepared by the procedures depicted in Scheme 2 (the synthetic procedure for the ligand of **Pd-B-4** can be found in the ESI†). The Pd(ii) complexes were synthesized by reacting the corresponding ligands with Pd(OAc)_2_ in refluxing CH_3_CO_2_H, and were purified by flash column chromatography on a SiO_2_ column using a hexane–ethyl acetate mixture as eluent. The ^1^H NMR data of all reaction intermediates, ligands and metal complexes are given in the ESI.† Assignments of the ^1^H signals are based on 2D COSY and NOESY NMR spectra. Complexes **Pd-B-1**, **Pd-B-3** and **Pd-B-4** show a downfield ^1^H signal at ∼10 ppm assignable to protons on the spiro-fluorene unit. The ^1^H NMR spectra of **Pd-B-1** at a temperature of 273–323 K are depicted in Fig. S1 of the ESI.† All the ^1^H signals retain their chemical shifts except that the protons on the spiro-fluorene become broader at temperatures >313 K. Thermal stability of **Pd-B-1**, **Pd-B-2**, **Pd-B-4**, **Pd-G-1**, **Pd-G-2** and **Pd-N-1** has been investigated by thermogravimetric analysis (TGA). Except for **Pd-B-4**, all the complexes show a decomposition temperature (*T*_d_) at 315 to 392 °C (Fig. S2 in the ESI†). High purity samples of **Pd-B-1**, **Pd-B-2**, **Pd-G-1** and **Pd-G-2** were obtained by sublimation at 285 to 300 °C under 4 × 10^–5^ Torr. The structures of **Pd-B-1** and **Pd-B-3** were characterized by X-ray crystallography.

### X-ray crystal structure of **Pd-B-1** and **Pd-B-3**

**Fig. 1:**
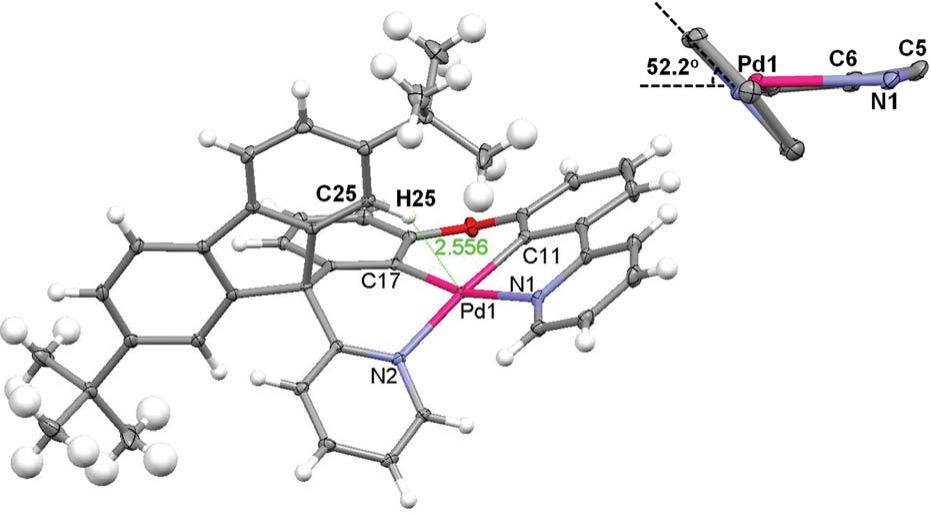
X-ray crystal structure of **Pd-B-1**. Thermal ellipsoids are drawn at the 35% probability level (note: the distance between the hydrogen atom residing on C25 and the normal plane of the Pd1–C11–C6–C5–N1 chelating ring is 2.556 Å); the inset depicts the angle between the pyridine ring and the aforementioned chelating ring.

Crystals of **Pd-B-1** and **Pd-B-3** were obtained by slow diffusion of Et_2_O into CHCl_3_ solutions of the metal complexes. Their X-ray crystal structures (Fig. 1 and S3†) reveal a distorted coordination geometry from planarity with C_phenyl_-Pd-N_pyridine_ angles of 165.96(7)–173.74(8)°. The Pd–N distances of 2.0968(19)–2.1441(18) Å are longer than those of the reported [Pd(O^N^C^N)] complexes (1.982(1)–2.061(3) Å).^9^ The spiro-fluorene moiety forces the pyridine ring nearby to twist at an angle of ∼52.2° (**Pd-B-1**) or ∼54.4° (**Pd-B-3**) to the normal plane of the Pd1–C11–C6–C5–N1 (**Pd-B-1**) or Pd1–C6–C7–C5–N1 (**Pd-B-3**) ring, resulting in a highly distorted, rigid ligand scaffold (inset in Fig. 1 and S3†). It is noted that the hydrogen atoms residing on C25 (**Pd-B-1**) and C42 (**Pd-B-3**) point toward the Pd1–C11–C6–C5–N1 (**Pd-B-1**) and Pd1–C6–C7–C5–N1 (**Pd-B-3**) chelating rings, respectively, with a distance separation of 2.543–2.556 Å (Fig. 1 and S3†). There is no close Pd···Pd contact in the crystal structures.

### Electrochemical properties of Pd(ii) complexes

**Fig. 2:**
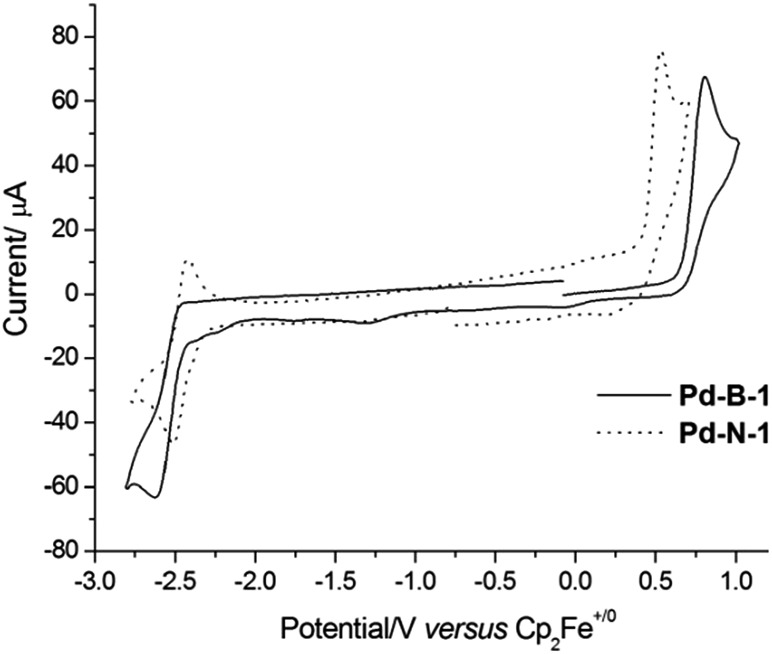
Cyclic voltammograms of **Pd-B-1** and **Pd-N-1** in DMF (0.1 mol dm^–3 *n*^Bu_4_NPF_6_ as supporting electrolyte) at 298 K. Scan rate: 100 mV s^–1^.

The electrochemical data of the Pd(ii) complexes in DMF (0.1 mol dm^–3 *n*^Bu_4_NPF_6_ as the supporting electrolyte) are summarized in Table 1. Cyclic voltammograms of **Pd-N-1**, **Pd-B-1**, **Pd-B-2**, **Pd-B-4**, **Pd-G-1**, and **Pd-G-2** in DMF show a quasi-reversible reduction couple at *E*_1/2_ of –2.16 to –2.54 V and an irreversible oxidation wave at *E*_pa_ of +0.53 to +0.81 V *versus* Cp_2_Fe^+/0^ (Fig. 2 and S4†). **Pd-N-1** shows a similar *E*_pa_ value (∼+0.53 V) but a more cathodic *E*red1/2 (*E*red1/2 with a cathodic shift of 260 mV) compared to those of **Pd-G-1** and **Pd-G-2**. The less cathodic *E*red1/2 of **Pd-G-1** and **Pd-G-2** is ascribed to the two electron-withdrawing F substituents on the [O^N^C^N] ligand of **Pd-G-1** and **Pd-G-2** which cause lowering of the LUMO of these two complexes when compared to **Pd-N-1**. On the other hand, complexes **Pd-N-1**, **Pd-G-1**, and **Pd-G-2** have similar *E*_pa_ values attributable to their HOMOs being mainly localized on the phenoxide ion of the [O^N^C^N] ligand (MO surfaces was shown in Fig. S5 in the ESI†). For the Pd(ii) complexes with [N^C^C^N] ligands, **Pd-B-1**, **Pd-B-2** and **Pd-B-4**, they display similar *E*_pa_ values but different *E*red1/2 values. This is attributed to their HOMOs being mainly localized on the C-deprotonated phenyl group of the [N^C^C^N] ligand with some electron density delocalized to the bridging fluorene unit (**Pd-B-1** and **Pd-B-4**) or oxygen atom (**Pd-B-2**). However, the LUMO is mainly localized on the C^N fragment (the part without the linker unit, see MO surfaces in Fig. S5†) such that **Pd-B-1** and **Pd-B-2** have similar *E*red1/2; for **Pd-B-4**, due to the extended π-conjugation of the 1-isoquinoline pendant moiety of the C^N unit, the LUMO is stabilized resulting in less cathodic *E*red1/2 compared to **Pd-B-1**.

**Table 1:** Electrochemical data of selected complexes

	*E*_pa_ ^*a*^ (V)	*E* red 1/2 ^*a*^ (V)	HOMO^*b*^ (eV)	LUMO^*b*^ (eV)
**Pd-N-1**	0.53	–2.47	–5.33	–2.33
**Pd-B-1**	0.81	–2.54	–5.61	–2.26
**Pd-B-2**	0.73	–2.44	–5.53	–2.36
**Pd-B-4**	0.81	–2.16	–5.61	–2.64
**Pd-G-1**	0.53	–2.24	–5.33	–2.56
**Pd-G-2**	0.59	–2.21	–5.39	–2.59

^*a*^Determined in DMF at 298 K with 0.1 mol dm^–3 *n*^Bu_4_NPF_6_ as the supporting electrolyte. Scan rate: 100 mV s^–1^. Values are *versus* Cp_2_Fe^+/0^. Cp_2_Fe^+/0^ occurs at 0.06–0.08 V *versus* Ag/AgNO_3_ (0.1 mol dm^–3^ in CH_3_CN) reference electrode.

^*b*^Estimated from *E*_pa_/*E*_1/2_ using a Cp_2_Fe^+/0^ value of 4.8 eV below the vacuum level.

When the anodic scan was performed prior to the cathodic scan, the cyclic voltammograms of **Pd-B-1** and **Pd-B-2** showed cathodic waves at around +0.03 V, –1.22 V (**Pd-B-1**) and –0.09 V (**Pd-B-2**), respectively (Fig. S6†). As these cathodic waves were not observed in the initial cathodic scan, they are attributable to the decomposition of the electrochemically oxidized species of the palladium(ii) complexes.

### UV/Vis absorption and steady-state emission measurements

**Fig. 3:**
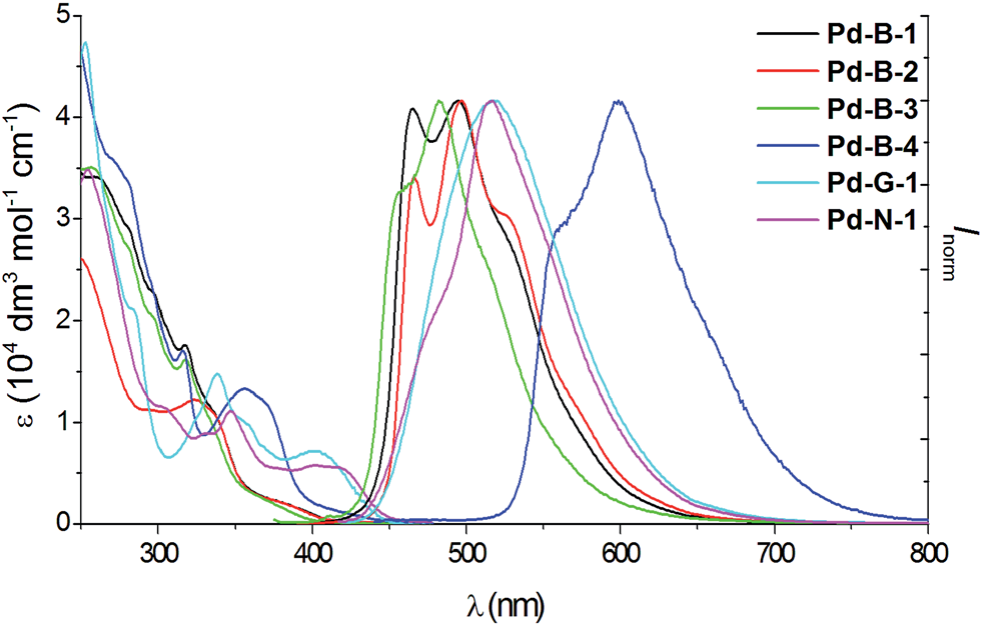
UV/Vis absorption and emission spectra of **Pd-B-1**, **Pd-B-2**, **Pd-B-3**, **Pd-B-4**, **Pd-G-1** and **Pd-N-1** in degassed CH_2_Cl_2_ solution (4 × 10^–5^ mol dm^–3^) at room temperature.

All the complexes show intense absorption bands at 250–370 nm with molar absorptivities (*ε*) of the order of 10^4^ dm^3^ mol^–1^ cm^–1^, and less intense absorption bands at 370–450 nm with *ε* values of the order of 10^3^ dm^3^ mol^–1^ cm^–1^ (Fig. 3). Compared to **Pd-B-1**, **Pd-B-2**, and **Pd-B-3**, the low-energy absorption bands of **Pd-B-4** are red-shifted. Such red shifts can be attributed to the lower LUMO level of **Pd-B-4** as revealed by the electrochemical data and DFT calculations on these complexes. Complexes **Pd-G-1**, **Pd-G-2** and **Pd-N-1** show more intense absorption bands at *λ* > 400 nm. The intense absorptions at 250–320 nm (**Pd-B-1**) and 250–350 nm (**Pd-G-1** and **Pd-G-2**) are attributed to ^1^IL (intraligand) π → π* transitions as the free ligands show similar absorption bands (Fig. S7 and S8 in the ESI†) and insignificant changes in absorption maxima with solvent polarity are observed (Fig. S9 and S10 in the ESI†). On the other hand, the less intense lower-energy absorption bands display negative solvatochromic shifts (*λ*_max_ of the lowest energy absorption band of **Pd-B-1** is blue-shifted from 421 nm in hexane to 383 nm in CH_3_OH; *λ*_max_ of **Pd-G-2** is blue-shifted from 446 nm in hexane to 391 nm in CH_3_OH), indicating that these absorptions are charge transfer in nature (Fig. S9 and S10 in the ESI†).^17^

All the Pd(ii) complexes are strongly emissive with peak maxima (*λ*_em_) at 466–599 nm, lifetimes (*τ*_obs_) in the range of 48–272 μs, and quantum yields (*Φ*_em_) of up to 0.47 in degassed CH_2_Cl_2_ solutions and 0.64 in thin film samples at room temperature (Fig. 3 and Table 2). Increasing solvent polarity results in a blue shift in the emission maximum of **Pd-B-1** (*λ*_max_ = 468 [toluene]; 464 nm [DMF]) and **Pd-G-2** (*λ*_max_ = 506 nm [hexane]; 501 nm [CH_3_OH]) (Fig. S11 and S12 in the ESI†). This finding, together with the long *τ*_obs_ in the 10 to 100 μs range, suggests that the emission of these Pd(ii) complexes is mainly derived from ^3^IL π→ π* excited states.^17^ The emission energy of **Pd-B-1** can be tuned by ligand modification. For example, **Pd-B-3** which has two fluorine substituents on the cyclometalated ligand shows a blue-shifted (∼10 nm) emission band whereas **Pd-B-4**, with a 1-isoquinoline ring instead of the pyridine ring in **Pd-B-1**, shows a remarkable red-shifted emission band (*λ*_max_ at 599 nm) compared to that of **Pd-B-1**. However, the *Φ*_em_ values of **Pd-B-3** and **Pd-B-4** are lower than that of **Pd-B-1** (0.47 [**Pd-B-1**] *versus* 0.20 [**Pd-B-3**] *versus* 0.07 [**Pd-B-4**]). Although the emission spectra of **Pd-B-1** and **Pd-B-2** are similar, the emission intensity ratio of the *v*′′ = 1 to *v*′′ = 0 transition is higher for **Pd-B-2**. Together with the faster *k*_nr_ (*k*_nr_ = 5.00 × 10^3^ s^–1^ [**Pd-B-1**]; 1.27 × 10^4^ s^–1^ [**Pd-B-2**]), the emissive excited state of **Pd-B-2** has a greater structural distortion and hence a lower *Φ*_em_ for **Pd-B-2** (*Φ*_em_ = 0.47 [**Pd-B-1**] *versus* 0.39 [**Pd-B-2**]) is found. The effect of temperature on the emission of **Pd-B-1** in toluene has been investigated (Fig. S13 in the ESI†). Upon increasing the temperature from 275 K to 353 K, only a small change in the emission intensity was observed (a decrease of 6.2% at 353 K).

**Table 2:** Photo-physical characteristics of the Pd(ii) complexes

Complex	UV/Vis absorption *λ*_abs_ (nm) (*ε* (10^4^ dm^3^ mol^–1^ cm^–1^))^*a*^	Emission		
		*λ*_max_ (nm) (*τ*_obs_ (μs))^*a*^	*Φ*_em_	*k*_r_; *k*_nr_^*f*^ (s^–1^)
**Pd-B-1**	259 (3.39), 282 (2.83), 298 (sh, 2.21), 317 (1.73), 339 (0.98), 387 (sh, 0.17)	466, 495 (106)	0.47^*b*^; 0.64^*c*^	4.43 × 10^3^; 5.00 × 10^3^
**Pd-B-2**	249 (2.61), 294 (1.12), 323 (1.21), 338 (sh, 1.02), 387 (sh, 0.16)	466, 497 (48)	0.39^*b*^; 0.54^*c*^	8.13 × 10^3^; 1.27 × 10^4^
**Pd-B-3**	257 (3.49), 281 (sh, 2.73), 298 (sh, 2.01), 318 (1.61), 335 (sh, 0.97), 380 (sh, 0.16)	456, 483 (69)	0.20^*b*^; 0.09^*c*^	2.90 × 10^3^; 1.16 × 10^4^
**Pd-B-4**	271 (3.57), 280 (sh, 3.38), 297 (sh, 2.27), 3.16 (1.70), 356 (1.33), 372 (sh, 1.12), 404 (sh, 0.18)	599 (44)	0.07^*d*^; 0.04^*c*^	1.59 × 10^3^; 2.11 × 10^4^
**Pd-G-1**	253 (4.75), 284 (sh, 2.10), 323 (sh, 1.00), 338 (1.48), 357 (sh, 1.00), 403 (br, 0.71)	517 (55.4)	0.22^*d*^; 0.27^*e*^	3.97 × 10^3^; 1.41 × 10^4^
**Pd-N-1**	254 (3.48), 301 (sh, 1.16), 329 (sh, 0.88), 347 (1.10), 400 (br, 0.57), 422 (sh, 0.52)	515 (272)	0.18^*d*^; 0.01^*c*^	6.62 × 10^2^; 3.01 × 10^3^

^*a*^Determined in degassed CH_2_Cl_2_ (2 × 10^–5^ mol dm^–3^).

^*b*^Emission quantum yield was measured by using the optical dilute method with BPEA (9,10-bis(phenylethynyl)anthracene) in degassed benzene as the reference (*Φ*_em_ = 0.85).

^*c*^Emission quantum yield was measured in PMMA thin film with a dopant concentration of 10 wt%.

^*d*^Emission quantum yield was measured by the optical dilution method with [Ru(bpy)_3_](PF_6_)_2_ (bpy = 2,2′-bipyridine) in degassed CH_3_CN as the reference (*Φ*_em_ = 0.062).

^*e*^Emission quantum yield was measured in mCP thin film with a doping concentration of 10 wt%.

^*f*^Radiative decay rate constant estimated from the equation *k*_r_ = *Φ*_em_/*τ*; nonradiative decay rate constant estimated from the equation *k*_nr_ = (1 – *Φ*_em_)/*τ*.

#### Variable-temperature emission lifetime of **Pd-B-1** in the solid state and in PMMA

**Fig. 4:**
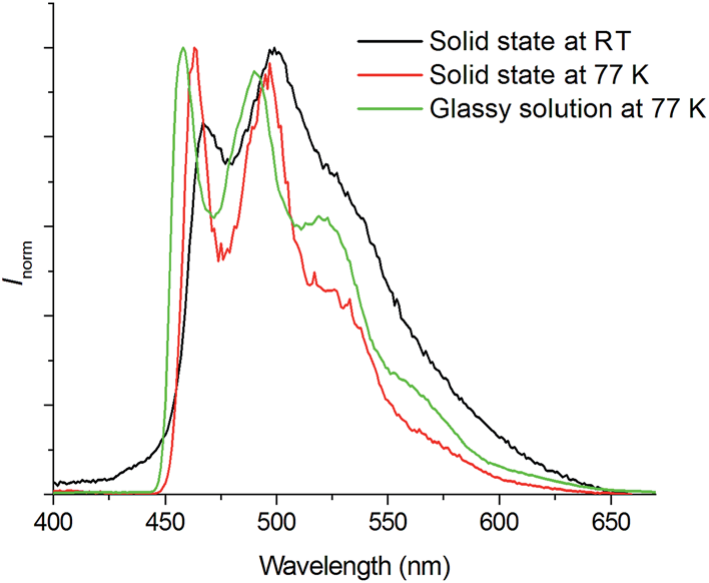
Emission spectra of **Pd-B-1** in the solid state at room temperature (black line) and 77 K (red line), and glassy solution (2-methyltetrahydrofuran at 77 K, green line) (*λ*_ex_ = 350 nm).

The emission spectrum of **Pd-B-1** in the solid state resembles that in CH_2_Cl_2_ solution (Fig. 4). At 77 K, the emission spectra of **Pd-B-1** in the solid state and in the glassy solution (1 × 10^–5^ mol dm^–3^ in 2-methyltetrahydrofuran) are similar, showing slightly blue-shifted, well-resolved emission bands relative to the solid state emission at room temperature (Fig. 4). No emission from excimer/ground-state aggregates is observed. The emission lifetimes of **Pd-B-1** in the solid state and in PMMA (5% dopant concentration) at temperatures from 50 to 300 K are depicted in Fig. S14 in the ESI.† The emission lifetime of the complex in the solid state and in PMMA decreases from 55 μs to 2 μs and from 245 μs to 80 μs, respectively. As there is no abrupt change in the emission lifetime as the temperature is increased from 77 to 300 K (Fig. S14 in the ESI†), the emissive excited state of **Pd-B-1** is unlikely to undergo reverse intersystem crossing (RISC) from T_1_ to S_1_. Hence, thermally activated delayed fluorescence (TADF) is not expected.^4^,^18^ Instead, the decrease in emission lifetime with increasing temperature is ascribed to an increase in the accessibility of vibrational states and/or cross-over to non-radiative excited states at higher temperatures. The much longer emission lifetimes of **Pd-B-1** in PMMA relative to those in the solid state at various temperatures can be rationalized by the facile TTA in the solid state in which closer intermolecular interactions are allowed. This facile TTA also contributes to the more significant decrease in emission lifetime of **Pd-B-1** in the solid state as compared to that in PMMA.

#### DFT/TDDFT calculations on the Pd(ii) complexes

**Fig. 5:**
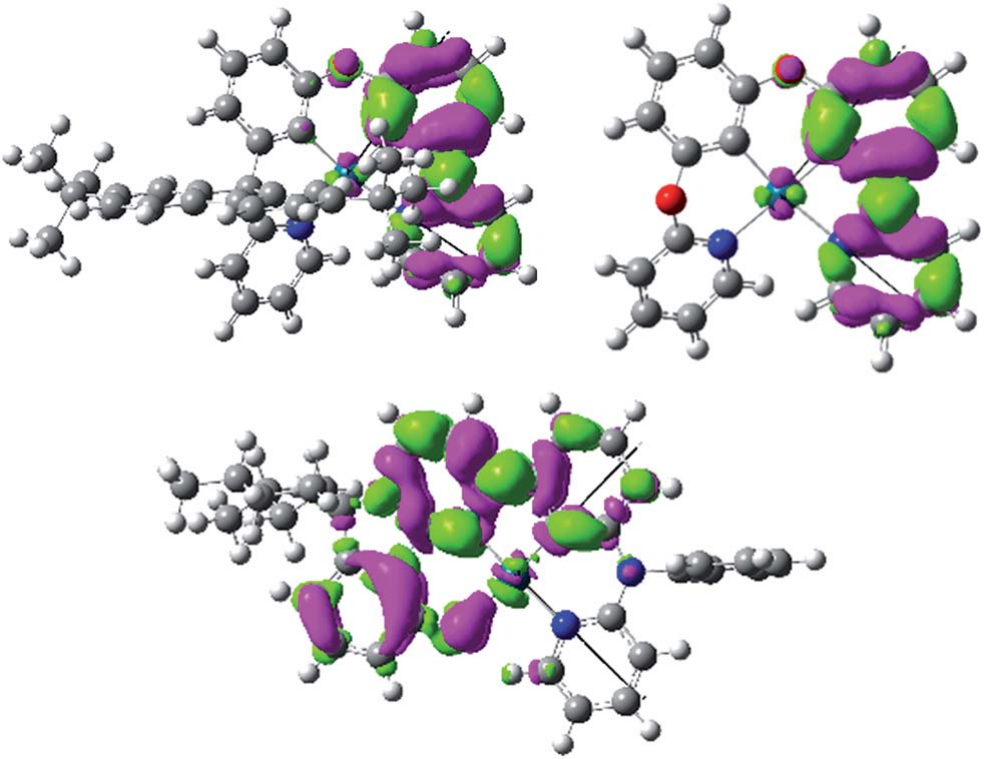
Electron density difference maps for the T_1_ → S_0_ transition of **Pd-B-1** (top left), **Pd-B-2** (top right), and **Pd-N-1** (bottom). Green: increase in electron density; magenta: decrease in electron density.

Based on the photophysical data, **Pd-B-1** shows a much longer *τ*_obs_ than **Pd-B-2**. This can be explained by the slower radiative decay and non-radiative decay rates of **Pd-B-1** compared to those of **Pd-B-2** (**Pd-B-1**: *k*_r_ = 4.43 × 10^3^ s^–1^ and *k*_nr_ = 5.00 × 10^3^ s^–1^; **Pd-B-2**: *k*_r_ = 8.13 × 10^3^ s^–1^ and *k*_nr_ = 1.27 × 10^4^ s^–1^). To shed light on the effect of the linker between the phenyl ring and the terminal pyridine ring (a spiro-fluorene [**Pd-B-1**] *versus* an O atom [**Pd-B-2**]) on the photophysics of the Pd(ii) complexes, we performed DFT/TDDFT calculations on **Pd-B-1** and **Pd-B-2**. Geometry optimizations using the DFT method revealed that the lowest triplet excited state (T_1_) is mainly of ^3^ππ*(ppy) character (ppy = phenylpyridine; see Fig. 5 for the electron density difference map of the T_1_ excited state of these two complexes). For **Pd-B-2**, the major contribution (80%) is H – 1 → LUMO transition where both H – 1 and LUMO are respectively π and π* orbitals of the ppy moiety of the tetradentate ligand (with less than 15% Pd character; see Fig. S15 in the ESI† for the MO surfaces and their compositions). For **Pd-B-1**, although the major contributions to the T_1_ excited state are derived from H – 2 → LUMO (43%) and H – 1 → LUMO (31%) transitions that have significant ^3^ππ*(ppy) character, there is also a non-negligible contribution from HOMO → LUMO transition (13%) with the HOMO localized on both phenyl moieties of the tetradentate ligand (see Fig. S16 in the ESI† for the MO surfaces). This means that the T_1_ excited state of **Pd-B-1** involves more delocalized orbitals than that of **Pd-B-2**. Hence, the T_1_ excited state of **Pd-B-1** experiences a smaller structural distortion than that of **Pd-B-2**, which is supported by the smaller Huang–Rhys factor (*S*) of the aromatic C=C/C=N stretching mode calculated for **Pd-B-1** (*S* = 0.98) compared to **Pd-B-2** (*S* = 1.02; see ESI†). Therefore, using an O atom instead of a spiro-fluorene as the linker would result in more localized orbitals and hence, a larger excited state structural distortion. Thus, a faster non-radiative decay rate of the T_1_ → S_0_ transition is observed for **Pd-B-2**. In addition, with an O atom as the linker, the metal character in both the H – 1 and LUMO are higher (see Fig. S15 and S16 in the ESI† for the MO characters). The higher metal character in the frontier orbitals gives rise to larger spin–orbit coupling (SOC), leading to an increase in both *k*_r_ and *k*_nr_. As a result, **Pd-B-2** shows faster *k*_r_ and *k*_nr_ values than those of **Pd-B-1**.

**Pd-N-1** has the longest emission lifetime among the complexes studied in this work. Based on the photophysical data, the radiative and non-radiative decay rates of **Pd-N-1** at room temperature are 6.62 × 10^2^ s^–1^ and 3.01 × 10^3^ s^–1^ respectively. Comparing these values with those of **Pd-B-1**, both complexes have similar *k*_nr_ but **Pd-N-1** has a significantly smaller *k*_r_. Examination of the T_1_ excited state of **Pd-N-1** by DFT/TDDFT calculations revealed that the triplet excited state is derived from HOMO → LUMO (58%), H – 1 → LUMO (21%), and H – 2 → LUMO (10%) transitions. The electron density difference map of the T_1_ excited state and MO surfaces are depicted in Fig. 5 and S17 in the ESI,† respectively. The orbitals are delocalized over the tetradentate ligand, thus the T_1_ → S_0_ transition is conceived to be accompanied with a small structural distortion, as in the case of **Pd-B-1** (Huang–Rhys factor for the C=C/C=N stretch is ∼0.89 for **Pd-N-1**). In addition, the metal contribution is found to be smaller in the case of **Pd-N-1** than in **Pd-B-1** (*e.g.*, for the dominant contribution to the T_1_ excited state of **Pd-N-1** [HOMO → LUMO] and **Pd-B-1** [H–2 → LUMO]; HOMO of **Pd-N-1** has only 3% Pd character but H – 2 of **Pd-B-1** has 7% Pd character). With this lower metal parentage in the T_1_ excited state and hence a smaller SOC, the *k*_r_ of **Pd-N-1** should be slower than that of **Pd-B-1**, such that the former has the longest emission lifetime of 272 μs.

#### Excited state properties of Pd(ii) complexes

##### Time-resolved fluorescence measurements

Femtosecond time-resolved fluorescence (fs-TRF) measurements of **Pd-N-1**, **Pd-G-1**, **Pd-B-1**, and **Pd-B-2** in CH_2_Cl_2_ solutions with excitation at 300 nm, have been conducted. The fs-TRF spectra and related kinetic decay of the fluorescence of the four complexes are shown in Fig. S18 and S19 in the ESI.† The fs-TRF spectra of **Pd-N-1** and **Pd-G-1** are similar (Fig. S18a and b†), featuring intensity decay accompanied with dynamic Stokes shift (DSS) of *λ*_max_ from 475 nm to 495 nm (**Pd-N-1**) and from 480 nm to 510 nm (**Pd-G-1**) in several picoseconds. The TRF of both complexes were observed to vanish completely by about 100 ps after photo-excitation. Analysis of the decay of TRF intensity (Fig. S19c and d†) revealed bi-exponential dynamics with time constants (*τ*_1_ and *τ*_2_) of 0.9 ps and 21 ps for **Pd-N-1** and 0.9 ps and 13 ps for **Pd-G-1**. The short-lived species in both cases (*τ*_1_ = 0.9 ps) are probably ascribable to the DSS arisen from the vibrational and structural relaxation while *τ*_2_ (21 ps and 13 ps for **Pd-N-1** and **Pd-G-1**, respectively) can be attributed to the process of intersystem crossing (ISC). On the other hand, fs-TRF of **Pd-B-1** and **Pd-B-2** (Fig. S19 in the ESI†) followed bi-exponential decay with *τ*_1_ of 0.3 (**Pd-B-1**)/0.4 ps (**Pd-B-2**) and *τ*_2_ of 1.0 (**Pd-B-1**)/0.6 ps (**Pd-B-2**), that is, the decays of fs-TRF of both **Pd-B-1** and **Pd-B-2** are faster than those of **Pd-N-1** and **Pd-G-1**. The short- and long-lived species are similarly accounted for by DSS (*λ*_max_ of both the complexes are shifted from 430 nm to 445 nm) and ISC of the complexes, respectively.

##### Nano-second, time-resolved absorption and emission measurements

**Fig. 6:**
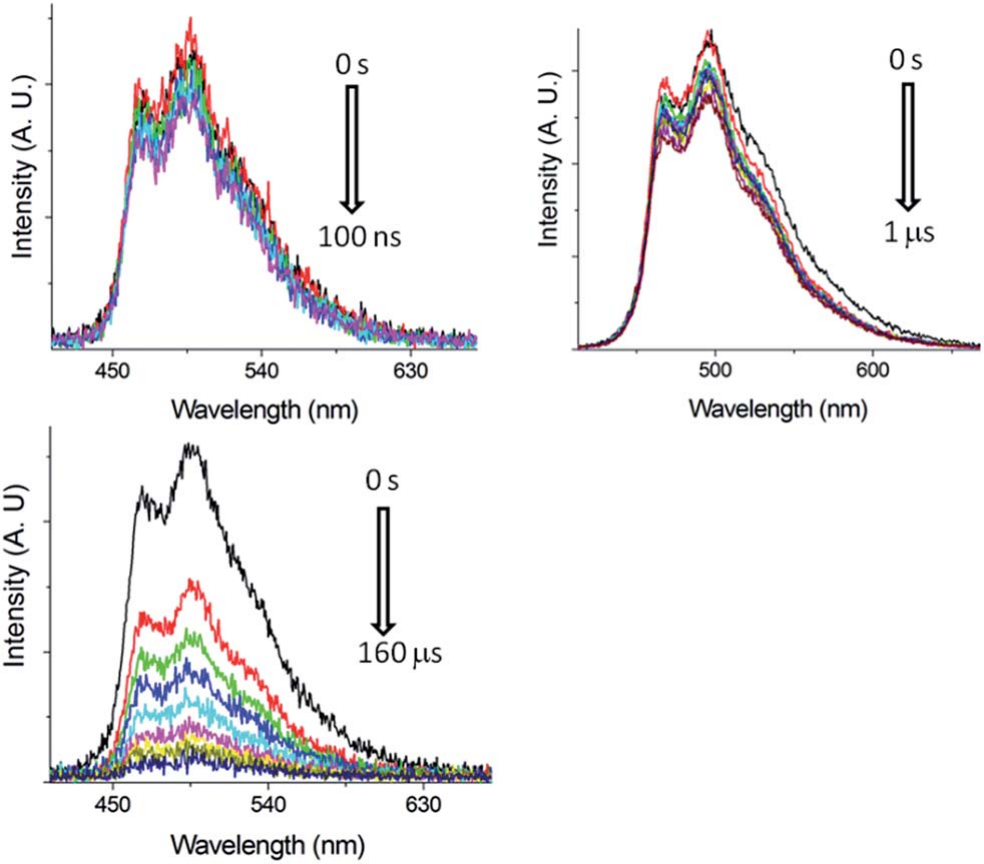
ns-TRE of **Pd-B-1** in CH_2_Cl_2_ (5 × 10^–5^ mol dm^–3^) with gate delays of 0–100 ns (top left), 0–1 μs (top right) and 0–160 μs (bottom) after laser excitation (355 nm).

Excited state dynamics of **Pd-B-1** on a nanosecond to microsecond time scale have been investigated by nanosecond time-resolved emission (ns-TRE) and transient absorption (ns-TA) measurements with the details depicted in Fig. S20 of the ESI.† The emission spectra with gate delays of 0–100 ns/0–1000 ns/0–160 μs at time intervals of 20 ns/100 ns/20 μs show similar emission profiles with no new emission band (Fig. 6). For the ns-TA spectra measured at similar time intervals, the decay kinetics at 409 nm has *τ*_obs_ of 60 μs which is similar to that of the ns-TRE at 466 nm, suggesting that the emission of **Pd-B-1** originates from the triplet excited state.

The long-lived excited state of **Pd-B-1** is found to be easily quenched by O_2_. In the presence of air, the time resolved absorption of **Pd-B-1** in CH_3_CN follows a faster decay than that in the degassed solution. Kinetic study of the TA at 398 nm reveals a *τ*_obs_ of 122 ns in aerated conditions, which is about 260-fold faster than that of the degassed sample (Fig. S21 in the ESI†). It is noteworthy that the absorption profiles for the degassed and non-degassed samples are almost the same as no new absorption band could be found in the absorption difference spectrum for the sample saturated with O_2_ (Fig. S21 in the ESI†). This suggests that the excited state of **Pd-B-1** is quenched by O_2_*via* an energy transfer pathway.

The excited state potentials, *E*(M*/M^–1^) and *E*(M^+^/M*), of **Pd-B-1**, **Pd-B-2**, **Pd-G-1** and **Pd-N-1** are estimated from the electrochemical and spectroscopic data using eqn (1) and (2)

1 *E*(M*/M^–1^) = *E*red1/2 + *E*_0–0_

2 *E*(M^+^/M*) = *E*_pa_ – *E*_0–0_

*E* red 1/2 and *E*_pa_ are obtained from the cyclic voltammograms of the complexes (Table 1). *E*_0–0_ is estimated from the triplet emission band. The *E*(M*/M^–1^) and *E*(M^+^/M*) of these complexes are in the range of +0.01 to +0.37 V and –1.90 to –2.08 V, respectively (Table 3).

**Table 3:** Electrochemical data of selected complexes

	*E*_pa_ ^*a*^ (V)	*E* red 1/2 ^*a*^ (V)	*E*_0–0_ ^*b*^ (eV)	*E*(M*/M^–1^) (V)	*E*(M^+^/M*) (V)
**Pd-N-1**	0.53	–2.47	2.48	0.01	–1.95
**Pd-B-1**	0.81	–2.54	2.71	0.17	–1.90
**Pd-B-2**	0.73	–2.44	2.70	0.26	–1.97
**Pd-G-1**	0.53	–2.24	2.61	0.37	–2.08

^*a*^Determined in DMF at 298 K with 0.1 mol dm^–3 *n*^Bu_4_NPF_6_ as the supporting electrolyte. Scan rate: 100 mV s^–1^. Values are *versus* Cp_2_Fe^+/0^. Cp_2_Fe^+/0^ occur at 0.06–0.08 V *versus* Ag/AgNO_3_ (0.1 mol dm^–3^ in CH_3_CN) reference electrode.

^*b*^Estimated from *λ*_max_ of the triplet emission band at 77 K.

**Fig. 7:**
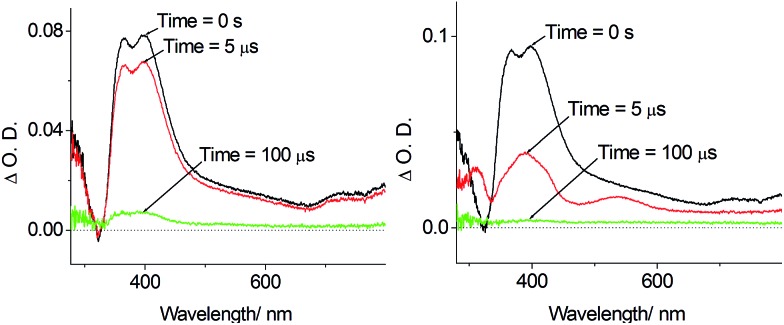
Left: Time resolved absorption difference spectra of **Pd-B-1** (5 × 10^–5^ mol dm^–3^) in degassed CH_3_CN monitored at 0 s, 5 μs and 100 μs; Right: time resolved absorption difference spectra of **Pd-B-1** (5 × 10^–5^ mol dm^–3^) and TMEDA (0.69 mol dm^–3^) in CH_3_CN monitored at 0 s, 5 μs and 100 μs.

The photo-induced electron transfer reactions between **Pd-B-1** and electron donor/electron acceptor were studied by nanosecond time resolved absorption spectroscopy. The time resolved absorption difference spectra of a CH_3_CN solution of **Pd-B-1** (5 × 10^–5^ mol dm^–3^) and tetramethylethylenediamine (TMEDA; 0.69 mol dm^–3^) showed strong absorptions at 390 and 543 nm at delay time > 5 μs after 355 nm laser excitation (Fig. 7 and S22 in the ESI†). Kinetic analysis of the decay at 398 nm revealed one short-lived component and one long-lived component with time constants of 3.1 and 36 μs, respectively. The short-lived species is assigned to the triplet excited state of **Pd-B-1** (Fig. S22 in the ESI†). Since the electron transfer reaction between **Pd-B-1** in the excited state and TMEDA is thermodynamically favoured with a driving force (Δ*E*) of +0.15 eV, the long-lived species is tentatively assigned to the one-electron reduced species of **Pd-B-1**, [**Pd-B-1**]^–^. The emission of **Pd-B-1** is quenched by MV^2+^ with a quenching rate constant (*k*_q_) of 8.66 × 10^9^ dm^3^ mol^–1^ s^–1^ (Fig. S23 in the ESI†). The time resolved absorption difference spectra of a CH_3_CN solution of **Pd-B-1** (5 × 10^–5^ mol dm^–3^) and MV^2+^ (2.5 × 10^–4^ mol dm^–3^) recorded with delay time of 0–2 μs after 355 nm laser excitation showed the strong absorptions at 398 and 605 nm (Fig. S24 in the ESI†). Kinetic analysis of the decay at 398 nm revealed a long-lived species with a *τ*_obs_ of ∼80 μs. These findings altogether reveal that the electron transfer reaction between **Pd-B-1** in the excited state and MV^2+^ has a Δ*E* of +1.01 eV, leading to the formation of MV^+^ cation radical.^19^

#### Photochemical properties of the Pd(ii) complexes

##### Photo-reductive C–C bond formation

The Pd(ii) complexes have long-lived excited states with *τ*_obs_ of up to 272 μs, which is useful for photo-catalysis. In this work, the photo-induced reductive cyclization of alkyl bromides using **Pd-B-1**, **Pd-B-2**, **Pd-G-1** or **Pd-N-1** as photo-redox catalyst has been examined. In the presence of diisopropylethylamine (^i^Pr_2_NEt) as a sacrificial electron donor in CH_3_CN and using a blue LED as the light source (*λ*_em_ = 420–520 nm, *λ*_max_ = 462 nm), cyclized products were found in the reactions of **Pd-N-1** with conversions and yields of up to 90% and 83%, respectively (Table 4). For **Pd-B-1** or **Pd-B-2**, no cyclized product was found with excitation at *λ*_ex_ > 370 nm using a xenon lamp as light source. The low absorptivities of **Pd-B-1** and **Pd-B-2** in the visible spectral region may contribute to this finding. Nevertheless, the cyclized product **A_2_** with 15% yield was found when TMEDA was used instead of ^i^Pr_2_NEt in the reaction of **Pd-B-1** (entry 3 in Table 4). This suggests that the photo-cyclization catalysed by **Pd-B-1** follows a reductive quenching pathway, by which the Pd(ii) complex in the excited state is reductively quenched by TMEDA [driving force (Δ*E*) = +0.15 V] but not by ^i^Pr_2_NEt (Δ*E* = –0.16 V).1a,b

**Table 4:** Photo-induced C–C bond formation of alkyl bromide

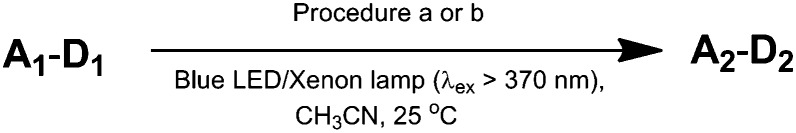					
Entry	Cat.	Reaction time (h)	Substrate	Product	Conv.; yield ^*f*^(%)
1^*a*^ ^,^^*c*^	**Pd-N-1**	10	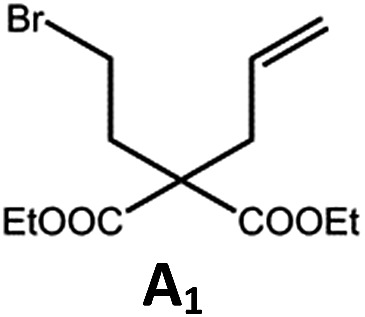	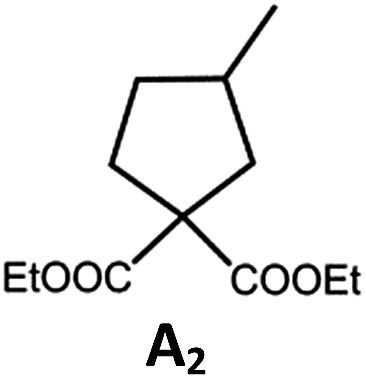	90; 83
2^*a*^ ^,^^*d*^	**Pd-B-1**	10			0; 0
3^*d*^ ^,^^*e*^	**Pd-B-1**	10			30; 15
4^*b*^ ^,^^*c*^	**Pd-N-1**	10	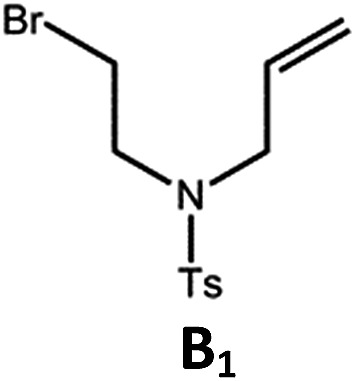	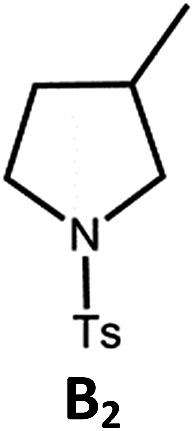	84; 63
5^*b*^ ^,^^*c*^	**Pd-N-1**	10	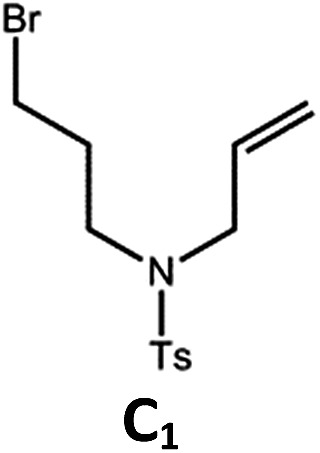	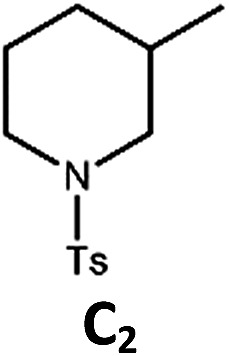	88; 66
6^*a*^ ^,^^*c*^	**Pd-N-1**	20	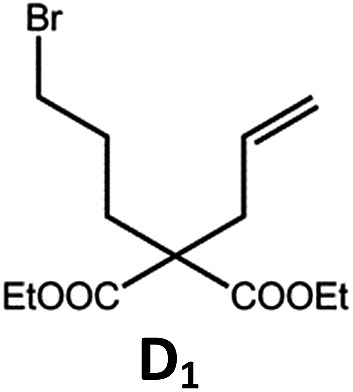	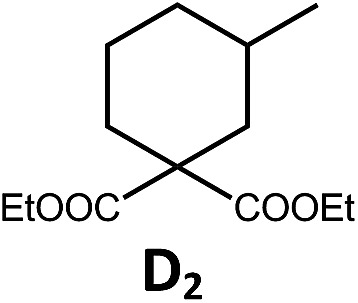	90; 68

^*a*^Procedure a: Pd(ii) complex (1 mol%) ^i^Pr_2_NEt (2 equiv.), CH_3_CN.

^*b*^Procedure b: Pd(ii) complex (1 mol%) ^i^Pr_2_NEt (5 equiv.), HCO_2_H (2 equiv.), CH_3_CN.

^*c*^The reaction mixture was irradiated with a blue LED (*λ*_max_ = 462 nm)

^*d*^The reaction mixture was irradiated with a xenon lamp (*λ*_ex_ > 370 nm).

^*e*^The procedure is the same as *a* except TMEDA was used instead of ^i^Pr_2_NEt.

^*f*^Determined by ^1^H NMR spectroscopy using 4,4′-dimethyl-2,2′-bipyridine as an internal standard. Ts = Tosylate.

The reaction mechanism for the photo-reductive C–C bond formation catalysed by **Pd-N-1** is different from that of **Pd-B-1**. This is because the reduction of **Pd-N-1** in the excited state by ^i^Pr_2_NEt, with a Δ*E* value of –0.32 V, is not thermodynamically favourable. On the other hand, no new absorption band attributable to [**Pd-N-1**]^+^ could be observed in the time-resolved absorption difference spectra of the solutions containing **Pd-N-1** and ^i^Pr_2_NEt, or **Pd-N-1** and substrate **A_1_**, after laser excitation (*λ*_ex_ = 355 nm; Fig. S25 in the ESI†). Also, the emissive excited state of **Pd-N-1** is not quenched by substrate **A_1_** or ^i^Pr_2_NEt. Nevertheless, as no product **A_2_** was observed in the reaction mixture with addition of 1 equivalent of 2,2,6,6-tetramethyl-1-piperidinyloxy (TEMPO), which is a well-known radical scavenger, the involvement of radical intermediate(s) in photo-catalysis is suggested.

##### Visible light photo-catalysis of [2 + 2] styrene cycloadditions

Complexes **Pd-B-1** and **Pd-N-1**, having high energy (up to 2.71 eV) long-lived triplet excited states with lifetimes up to 272 μs, were found to catalyse the formation of [2 + 2] cycloadducts **E_2_–H_2_** from **E_1_–H_1_** with substrate conversions and yields of up to 100% and 97% under irradiation with a xenon lamp (*λ* > 350 nm)/blue LED (*λ*_max_ at 462 nm)/23 W compact fluorescent lamp (CFL). No product was detected in the control experiment, that is, in the absence of Pd(ii) complexes (entry 5 in Table 5).

**Table 5:** Photo-catalysis of [2 + 2] styrene cycloaddition^*a*^

Entry	Cat.	Reaction time (h)	Substrate	Product	Conv.; yield (%) (d. r.)^*e*^
1	**Pd-B-1**	3	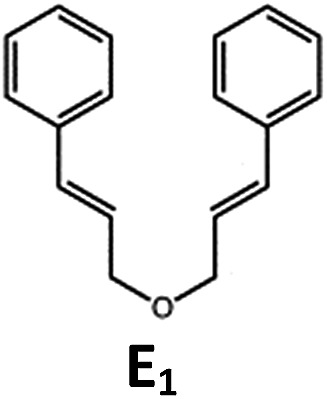	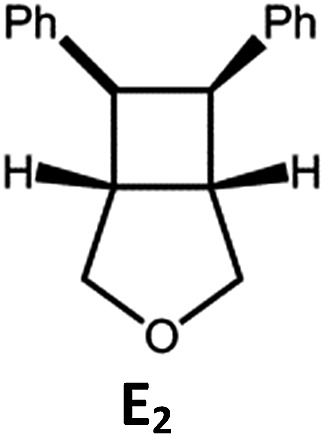	100; 88 (4 : 1)^*b*^
		4			27; 14 (4 : 1)^*d*^
2	**Pd-N-1**	4			100; 97 (4 : 1)^*c*^
		4			79; 73 (4 : 1)^*d*^
3	**Pd-B-4**	4			0; 0^*b*^
		4			0; 0^*c*^
4	*fac*-Ir(ppy)_3_	4			100; 77 (4 : 1)^*c*^
		4			76; 62 (4 : 1)^*d*^
5	—	4			0; 0^*b*^
6	**Pd-B-1**	8.5	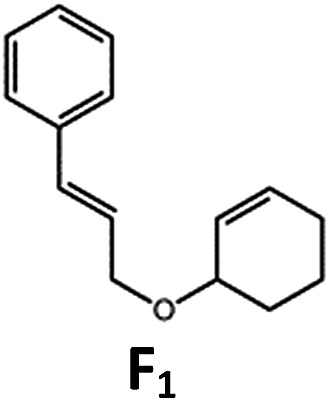	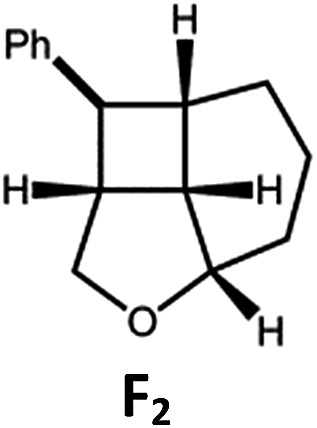	90; 74(>10 : 1)^*b*^
7	**Pd-N-1**	24			100; 82 (7 : 1)^*c*^
8	**Pd-B-1**	8.5	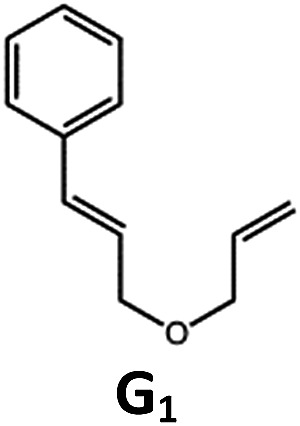	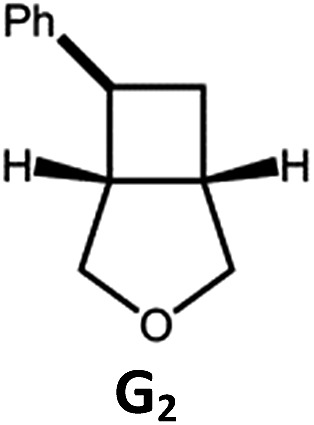	100; 94 (6 : 1)^*b*^
9	**Pd-N-1**	24			100; 97 (5 : 1)^*c*^
10	*fac*-Ir(ppy)_3_	24			100; 61 (6 : 1)^*c*^
11	**Pd-B-1**	8.5	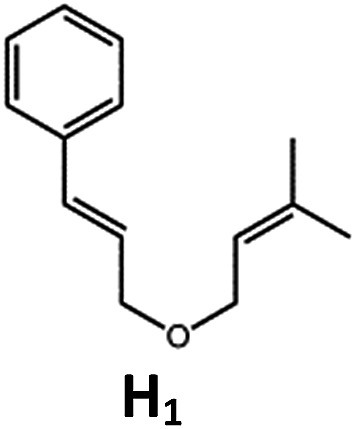	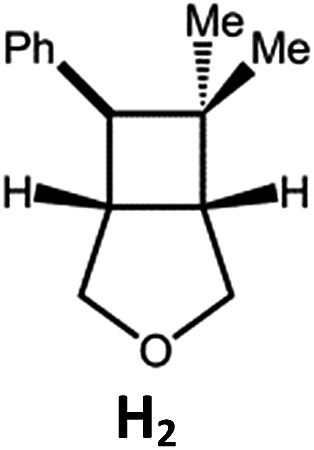	100; 85 (7 : 1)^*b*^
12	**Pd-N-1**	16			>99; 80 (7 : 1)^*c*^
13	*fac*-Ir(ppy)_3_	16			>99; 79 (6 : 1)^*c*^

^*a*^Reaction condition: metal complex (1 mol%), substrate (0.01 mol dm^–3^) in CH_3_CN.

^*b*^Xenon lamp (*λ*_ex_ > 350 nm) as the light source.

^*c*^blue LED (*λ*_em_ = 462 nm) as the light source.

^*d*^23 W compact fluorescent lamp (CFL) as the light source.

^*e*^Product yield determined by ^1^H NMR spectroscopy using 4,4′-dimethyl-2,2′-bipyridine as an internal standard.

The results obtained with the two Pd(ii) complexes are comparable to those of parallel experiments using *fac*-Ir(ppy)_3_ as the catalyst (Table 5) and those reported by Yoon using Ir(dF(CF_3_)ppy)_2_(dtbbpy)(PF_6_)_2_ under similar reaction conditions.14b,c The reactions presumably proceed through the Dexter energy transfer pathway as electron transfer reactions between the substrate **E_1_** and **Pd-B-1** or **Pd-N-1** are not thermodynamically favourable (Δ*E* = –0.94 to –1.10 eV; **Pd-B-1**/**Pd-N-1** in the excited state can act as oxidant or reductant, Table S5 in the ESI†). No cyclised product was observed when **Pd-B-4** was used as the sensitizer (entry 3 in Table 5) as this complex has a lower triplet energy (2.07 eV), thereby lending support to an energy transfer pathway. It is noted that the *k*_q_ between the triplet excited state of **Pd-B-1** and **E_1_** (*k*_q_ = 2.51 × 10^9^ dm^3^ mol^–1^ s^–1^) is one order of magnitude larger than that between **Pd-N-1** and **E_1_** (2.62 × 10^8^ dm^3^ mol^–1^ s^–1^) (Fig. S26 in the ESI†).

### Application of Pd(ii) complexes in blue and green OLEDs as well as in green and yellow PSF-OLEDs

Based on their electrochemical data and photo-physical properties (Tables 1 and 2), different device structures were used to fabricate green (**Pd-G-1** and **Pd-G-2**) and blue (**Pd-B-1** and **Pd-B-2**) OLEDs from these Pd(ii) complexes. For the devices with **Pd-G-1** and **Pd-G-2**, an architecture of ITO/MoO_3_ (2 nm)/TAPC (40 nm)/mCP(10 nm)/mCP: emitter(s) (20 nm)/TmPyPb (40 nm)/LiF (1.2 nm)Al (150 nm) was constructed. 4,4′-Cyclohexylidenebis[*N*,*N*-bis(4-methylphenyl)benzamine] (TAPC) and 3,3′-[5′-[3-(3-pyridinyl)phenyl][1,1′:3′,1′′-terphenyl]-3,3′′-diyl]bispyridine (TmPyPB) were the hole-transporting layer (HTL) and electron-transporting layer (ETL), respectively. In the emissive layer (EML), 1,3-bis(carbazol-9-yl)benzene (mCP) was used as the host, in which **Pd-G-1** or **Pd-G-2** was dispersed as the phosphorescent emitter. For yellow PSF-OLEDs, **Pd-G-1** or **Pd-G-2** was used as the phosphor sensitizer while 2,8-di-*tert*-butyl-5,11-bis(4-*tert*-butylphenyl)-6,12-diphenyltetracene (TBRb) was the yellow fluorescent emitter.20*a* An additional mCP layer was inserted between HTL and EML as an exciton-blocking layer (EBL) to confine triplet excitons inside the EML. In the cases of **Pd-B-1** and **Pd-B-2**, a more complicated device structure of ITO/MoO_3_ (2 nm)/TAPC (40 nm)/TCTA (10 nm)/CzSi (3 nm)/CzSi: emitter(s) (20 nm)/TSPO1 (10 nm)/TmPyPb (40 nm)/LiF (1.2 nm)/Al (150 nm) was used. Considering the high triplet energies of **Pd-B-1** and **Pd-B-2**, EML and EBL having higher triplet energies (*E*_t_) were used; 9-(4-*tert*-butylphenyl)-3,6-bis(triphenylsilyl)-9*H*-carbazole (CzSi; *E*_t_ = 3.02 eV) as the host in the EML, bilayer tris(4-carbazoyl-9-ylphenyl)amine (TCTA; *E*_t_ = 2.8 eV)/CzSi (*E*_t_ = 3.02 eV) as a hole-transporting EBL between the HTL and EML,21*b* and diphenyl-4-triphenylsilylphenyl-phosphineoxide (TSPO1; *E*_t_ = 3.36 eV) as an electron-transporting EBL between the EML and ETL.16*e***Pd-B-1** or **Pd-B-2** was used as the phosphorescent emitter in blue phosphorescent OLEDs or as the sensitizer in green PSF-OLEDs in which 9,10-bis[*N*,*N*-di-(*p*-tolyl)-amino]anthracene (TTPA) was used as the fluorescent emitter.20*b*

**Fig. 8:**
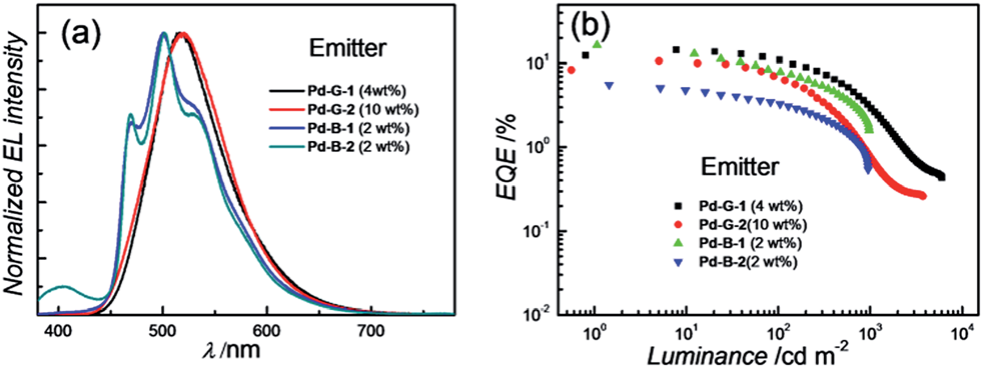
(a) Normalized EL spectra and (b) EQE-luminance characteristics of OLEDs with **Pd-G-1**, **Pd-G-2**, **Pd-B-1** and **Pd-B-2** with optimized dopant concentrations.

Normalized EL spectra and EQE-luminance characteristics of all phosphorescent OLEDs based on Pd(ii) complexes at optimized dopant concentrations are depicted in Fig. 8. The dependence of EL spectra and EQE on the doping concentrations of these Pd(ii) complexes are depicted in Fig. S27–S30 (ESI†) and the findings are summarized in Table 6. For the green light-emitting **Pd-G-1** and **Pd-G-2**, their electroluminescent (EL) spectra are almost identical to their solution photoluminescence (PL) spectra (Fig. 3). With increasing complex concentration, the EL spectra of both **Pd-G-1** and **Pd-G-2** showed a red shift in the emission maximum, attributable to intermolecular interactions of the complex (details are given in Fig. S27a and S28a of ESI†). However, no emission from aggregated states, such as excimer, was observed even at the high dopant concentration of 20 wt%. As depicted in Fig. 8b, a maximum EQE of 14.45% was achieved for the OLED with 4 wt% **Pd-G-1**. This is nearly 2-fold higher than that of other reported Pd(ii)-OLEDs.^9^ For the **Pd-G-2** device, a maximum EQE of 10.65% was achieved at a higher dopant concentration of 10 wt%. In addition, with increasing dopant concentration, the EQE of the **Pd-G-2** device decreases slowly (Fig. S28b in the ESI†), suggesting that triplet–triplet annihilation of **Pd-G-2** is less efficient. Normalized EL spectra and EQE-luminance curves of OLEDs with **Pd-B-1** and **Pd-B-2** respectively are depicted in Fig. 8. The EL spectra of **Pd-B-1** and **Pd-B-2** devices have similar profiles at a low dopant concentration of 2 wt% and sky blue emission with Commission Internationale De L'Eclairage (CIE) coordinates of (0.24, 0.46) and (0.24, 0.45), respectively. To our knowledge, these are the first examples of sky blue Pd-OLEDs. The EL performance of **Pd-B-1** and **Pd-B-2** devices varied with dopant concentration. The EL spectrum for the **Pd-B-1** device remained unchanged while the EL maximum of the **Pd-B-2** device red-shifted from 501 nm to 534 nm when the dopant concentration was increased from 2 to 10 wt% (Fig. S29a and S30a of ESI†). The dopant concentration-dependent EL behaviour of **Pd-B-1** and **Pd-B-2** devices is attributable to different structures of these two Pd(ii) complexes; the ligand of **Pd-B-1** is more sterically bulky (Fig. 1) and hence weak intermolecular interactions among the complex molecules are anticipated. Thus it is not surprising to find that the EL spectrum of the **Pd-B-1** device was quite insensitive to dopant concentration. A maximum EQE of 16.48% was achieved at a low luminance of ∼1 cd m^–2^ for the OLED with 2 wt% **Pd-B-1**. This value is comparable to those of the most efficient blue Pt-OLEDs.^21^ For the **Pd-B-2** device, the low EQE of 5.60% can be accounted for by the lower PL quantum yield of the Pd(ii) complex (Table 2).

**Table 6:** Key performance data of OLEDs with **Pd-G-1**, **Pd-G-2**, **Pd-B-1** and **Pd-B-2**

Complex (wt%)	CE^*a*^ (cd A^–1^)	PE^*b*^ (lm W^–1^)	EQE (%)		CIE^*c*^ (*x*, *y*)
			Max.	at 500 cd m^–2^	
**Pd-G-1** (4%)	46.11	48.29	14.45	5.72	0.27, 0.56
**Pd-G-1** (10%)	40.79	42.17	12.24	7.24	0.30, 0.58
**Pd-G-1** (20%)	20.07	20.97	6.54	3.11	0.34, 0.58
**Pd-G-2** (4%)	30.76	32.22	10.20	0.92	0.26, 0.51
**Pd-G-2** (10%)	33.90	35.50	10.65	2.11	0.27, 0.55
**Pd-G-2** (20%)	24.68	22.81	7.29	2.10	0.30, 0.58
**Pd-B-1** (2%)	42.62	39.38	16.48	4.00	0.24, 0.46
**Pd-B-1** (6%)	28.71	25.06	10.47	3.21	0.24, 0.46
**Pd-B-1** (10%)	17.36	15.15	6.31	2.16	0.24, 0.47
**Pd-B-2** (2%)	14.75	13.63	5.60	1.67	0.24, 0.45
**Pd-B-2** (6%)	16.25	15.60	5.64	1.12	0.26, 0.49
**Pd-B-2** (10%)	13.12	12.03	4.32	0.87	0.29, 0.52

^*a*^Max. current efficiency.

^*b*^Max. power efficiency.

^*c*^CIE coordinates at 500 cd m^–2^.

**Fig. 9:**
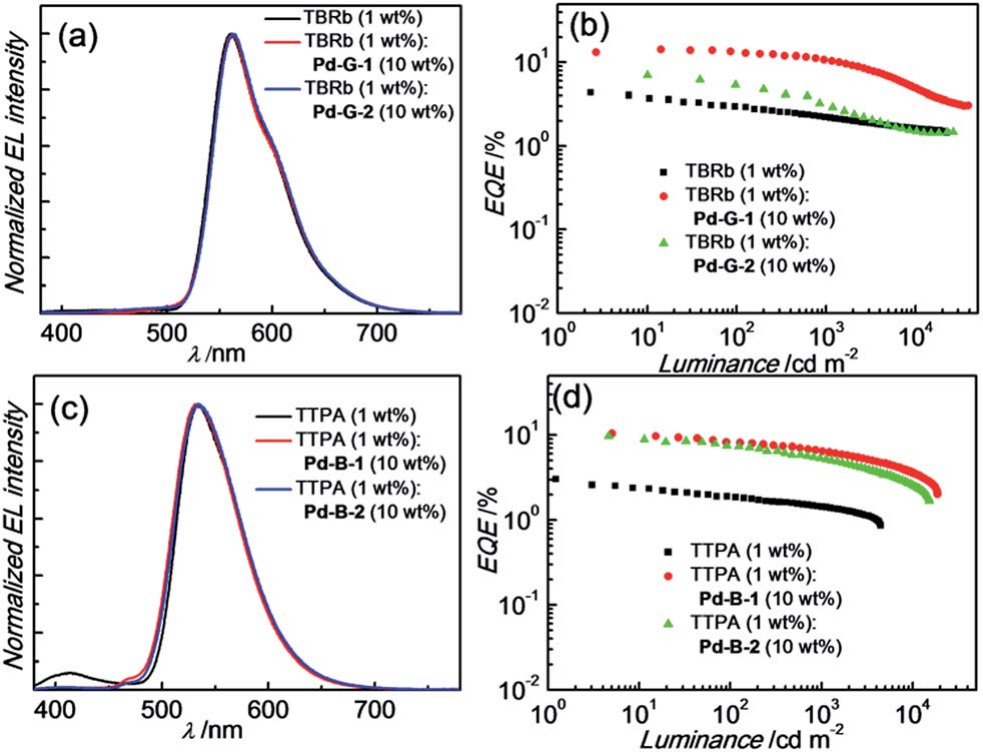
(a) Normalized EL spectra and (b) EQE-luminance characteristics of yellow PSF-OLEDs based on the emission of TBRb. (c) Normalized EL spectra and (d) EQE-luminance characteristics of green PSF-OLEDs based on the emission of TTPA.

For the Pd-OLEDs studied in this work, the efficiency roll-off with increasing luminance was quite significant (Fig. 8b). The EQEs of devices with **Pd-G-1** (4 wt%), **Pd-G-2** (10 wt%), **Pd-B-1** (2 wt%) and **Pd-B-2** (2 wt%) at 500 cd m^–2^ were 5.72%, 2.11%, 4.00% and 1.67%, corresponding to roll-offs of 60.4%, 80.2%, 75.7% and 70.2%, respectively. The rapid efficiency roll-off could be due to saturation of triplet excited states of the Pd(ii) complexes, which have very long emission lifetimes.^4^,^9^ Nevertheless, these long emission lifetimes render the Pd(ii) complexes useful sensitizers for PSF-OLEDs. Both yellow and green PSF-OLEDs using the Pd(ii) complexes as sensitizer have been investigated and the results are summarized in Table 7. The EL spectra and EQE-luminance performances of the yellow OLEDs using TBRb as fluorescent emitter with and without **Pd-G-1** or **Pd-G-2** sensitizer are depicted in Fig. 9a and b, respectively. Although the doping concentration of the Pd(ii) complex was 10-fold higher than that of TBRb, the yellow light-emitting devices with and without the Pd(ii) complexes as sensitizer showed identical EL spectra, revealing complete quenching of the phosphorescence of these Pd(ii) complexes *via* Förster resonance energy transfer (FRET) to TBRb. Consequently, a maximum EQE of 14.32% for the **Pd-G-1**-PSF OLED device was more than 3-fold higher than that of the one without Pd(ii) sensitizer. A relatively lower EQE of 7.0% for the **Pd-G-2**-PSF OLED device could be attributed to the bulky tetradentate ligand of **Pd-G-2** that may hamper energy transfer to TBRb. Meanwhile, the **Pd-G-1**-PSF OLED device showed a significant improvement of efficiency roll-off; this can be attributed to the expansion of the carrier recombination site and/or the reduction of exciton/polaron quenching.^22^ For the green PSF-OLEDs using 1 wt% fluorescent TTPA as the emitter, phosphorescent **Pd-B-1** or **Pd-B-2** was employed as the sensitizer (Fig. 9c). Like those of the yellow PSF-OLEDs depicted in Fig. 9a, phosphorescence from the Pd(ii) complex in the green **Pd-B-1** or **Pd-B-2** PSF-OLED device was almost completely quenched and a strong emission from TTPA was observed. It is noteworthy that without the phosphorescent Pd(ii) sensitizer, a low EQE of 3.14% was recorded in the TTPA-only device (Fig. 9d). On the other hand, although the efficiency of the **Pd-B-2**-based OLED was lower than that of the **Pd-B-1** one (Fig. 8b), the EQEs of both PSF-OLEDs with **Pd-B-1** or **Pd-B-2** as the sensitizer were similar; 10.41% for the former and 9.73% for the latter. These values are more than 3-fold higher than the EQE of the TTPA-only device. Compared to the reported PSF-OLEDs using phosphorescent Ir(iii) complexes as sensitizer, improved colour purity and higher efficiency have been realized in the PSF-OLEDs with the phosphorescent Pd(ii) sensitizers herein described.^15^

**Table 7:** Key performance data of PSF-OLEDs

EML	CE^*a*^ (cd A^–1^)	PE^*b*^ (lm W^–1^)	EQE (%)		CIE^*c*^ (*x*, *y*)
			Max.	at 1000 cd m^–2^	
TBRb (1%)	14.88	13.75	4.38	2.21	0.47, 0.51
**Pd-G-1** (10%): TBRb (1%)	48.70	45.00	14.32	10.60	0.47, 0.52
**Pd-G-2** (10%): TBRb (1%)	22.44	20.14	7.00	3.10	0.47, 0.51
TTPA	11.80	11.60	3.14	1.45	0.32, 0.60
**Pd-B-1** (10%), TTPA (1%)	38.85	38.14	10.41	6.55	0.33, 0.60
**Pd-B-2** (10%), TTPA (1%)	36.82	36.15	9.73	5.24	0.33, 0.60

^*a*^Max. current efficiency.

^*b*^Max. power efficiency.

^*c*^CIE coordinates at 1000 cd m^–2^.

Preliminary studies on the operational stability of OLEDs with **Pd-G-1** as the emitter and PSF-OLEDs with **Pd-G-1** as the phosphorescent sensitizer were undertaken, using the device structure of ITO/MoO_3_ (5 nm)/NPB (70 nm)/mCBP: dopant(s) (30 nm)/BAlq (10 nm)/Alq (30 nm)/LiF (1.2 nm)/Al (150 nm).^23^ In these devices, *N*,*N*′-di(1-naphthyl)-*N*,*N*′-diphenyl-(1,1′-biphenyl)-4,4′-diamine (NPB) was used as the hole-transporting layer, with 3,3-di(9*H*-carbazol-9-yl)biphenyl (mCBP) as the host material, bis(2-methyl-8-quinolinolato-N1,O8)-(1,1′-biphenyl-4-olato)aluminum (BAlq) as the hole-blocking layer, and tris-(8-hydroxyquinoline)aluminum (Alq) as the electron-transporting layer. **Pd-G-1** (10 wt%) and **Pd-G-1** (10 wt%):TBRb (1 wt%) were used as dopant(s) for Pd-OLED and PSF-OLED, respectively. The dependence of relative luminance on operation time of both devices is depicted in Fig. S31 (in the ESI†). The Pd-OLED and the Pd(ii)-PSF-OLED were operated at constant current densities of 10 and 20 mA cm^–2^, respectively. For the Pd-OLED, its lifetime at 90% initial luminance (LT_90_) was found to be 135 h. With the formula LT_90_(*L*_1_) = LT_90_(*L*_0_) × (*L*_0_/*L*_1_)^1.7^,^24^ where *L*_1_ and *L*_0_ respectively represent the objective and experimental (930 cd m^–2^, here) initial luminance, LT_90_ at an objective luminance of 100 cd m^–2^ was estimated to be 5980 h. For the yellow PSF-OLED, a LT_90_ of 182 h was found at *L*_0_ = 3810 cd m^–2^, corresponding to more than 80 000 h at the objective luminance of 100 cd m^–2^. The longer device lifetime of the yellow PSF-OLED can be attributed to the slower efficiency roll-off of this device as well as the stability of the fluorescent emitter TBRb.

## Discussion

All the Pd(ii) complexes were synthesized by reacting Pd(OAc)_2_ with the corresponding ligand in glacial acetic acid. ^1^H NMR spectra of **Pd-B-1** at 273 K to 323 K show that the ^1^H signals of spiro-fluorene unit become broader at temperatures >313 K whereas all other ^1^H signals retain their chemical shifts and shapes. This finding is suggestive of swinging of the spiro-fluorene moiety at higher temperatures, resulting in indistinguishable chemical environments experienced by the protons. The minimal changes found for the other proton signals of **Pd-B-1** at elevated temperatures are attributable to the rigid ligand scaffold of this complex. It is noted that complexes **Pd-B-1**, **Pd-B-3** and **Pd-B-4** display a ^1^H signal at ∼10 ppm corresponding to the aromatic C–H proton of the spiro-fluorene. As aromatic C–H protons are usually found at 6–9 ppm, the downfield ^1^H signal at ∼10 ppm could be attributed to the C–H···π/C–H···Pd interactions, which has also been revealed in the X-ray crystal structures of **Pd-B-1** and **Pd-B-3**. Due to the C–H···π/C–H···Pd interactions, the C–H(H25) protons of the spiro-fluorene unit were observed to point into the metal chelating ring with distances of 2.543–2.556 Å.^25^

**Fig. 10:**
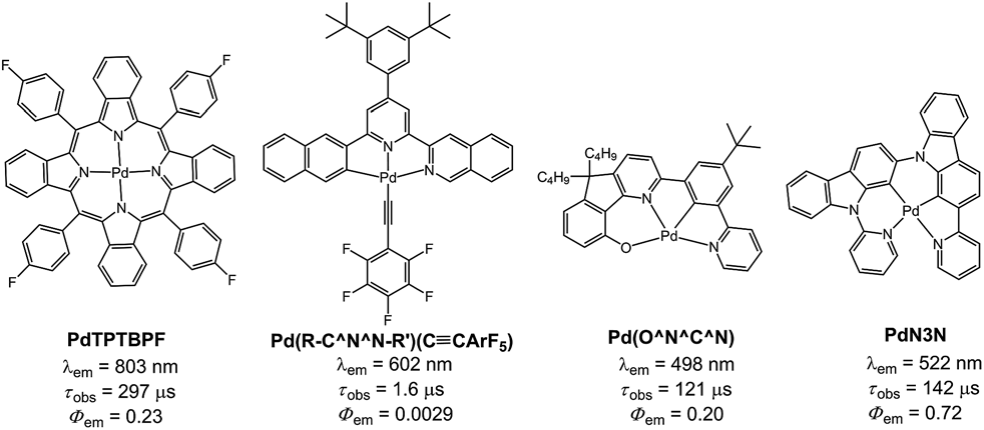
Selected examples of phosphorescent Pd(ii) complexes.

Palladium(ii) complexes are seldom reported to display intense phosphorescence in the blue to green spectral region. It is generally conceived that the d–d excited states are close in energy to, or lower in energy than, the emissive excited state(s) of most reported luminescent Pd(ii) complexes containing non-porphyrin ligands.^11^ Population of the d–d state leads to severe structural distortion and hence facile non-radiative decay of the excited state. In literature, palladium(ii) complexes of benzoporphyrin and naphthoporphyrin (Fig. 10) were reported to show phosphorescence in the red to near-infrared region (673–882 nm) with *Φ*_em_ and *τ*_obs_ of up to 23% and 520 μs, respectively. These Pd(ii) porphyrin complexes have been widely used for TTA and oxygen sensing.^26^ The significant energy difference between the d–d and low energy emissive ^3^ππ* excited states of the porphyrin ligand is the main reason accounting for the observed intense phosphorescence from Pd(ii) porphyrins. In previous work, we showed that Pd(ii) complexes containing C-deprotonated R–C^N^N–R′ and pentafluorophenylacetylide ligands display orange to red phosphorescence (570–600 nm) in CH_2_Cl_2_ solutions at high concentrations (Fig. 10).11*h* This finding was attributed to the formation of intermolecular aggregates where the energy level of the emissive triplet excited state would be lowered upon aggregation, thereby reducing the quenching *via* thermal population of the d–d state. Nevertheless, high energy emissive excited states in the blue to green spectral region, which are desirable in the context of photo-catalysis and energy down conversion processes, are sparsely found in Pd(ii) complexes. To overcome this challenge, we designed luminescent Pd(ii) complexes with ligands having a rigid scaffold and strong donor atoms for minimizing structural distortion in the T_1_ excited state and for destabilizing the d–d excited state to a thermally inaccessible energy level. Based on this design strategy, we previously reported a series of Pd(ii) complexes with the tetradentate [O^N^C^N] ligands showing green phosphorescence at 498–540 nm with *Φ*_em_ and *τ*_obs_ of up to 22% and 122 μs, respectively (Fig. 10).^9^ These were the first reported Pd(ii) complexes which display long-lived and high-energy triplet excited states. Subsequently, Li and co-workers reported two Pd(ii) complexes, namely **PdN3N** and **PdN3O** (Fig. 10), which display intense green phosphorescence in CH_2_Cl_2_ (*λ*_em_ ∼ 530 nm; *Φ*_em_ = 0.72–0.76) and metal-assisted delayed fluorescence (MADF).23*b*

The Pd(ii) complexes described herein are supported by the tetradentate ligand systems [N^C^C^N] and [O^N^C^N]; they display intense phosphorescence with *λ*_em_ = 466–599 nm, long emission lifetime (*τ*_obs_ up to 272 μs), and *Φ*_em_ up to 0.47 in CH_2_Cl_2_ and 0.64 in PMMA. The long emission lifetime is attributed to little metal character and small structural distortion in the T_1_ excited state, rendering a slower decay from the T_1_ to S_0_, as is also revealed by the DFT/TDDFT calculations. In addition, the Pd(ii) complexes described in this work feature fast ISC from singlet to triplet manifolds with time constants of 0.6 to 21 ps. These time constants are about one to two orders of magnitude larger than those of the Pt(ii) analogues which is attributed to the larger spin–orbit coupling constant of Pt(ii) ion than Pd(ii) ion. Notably, the temporal evolution of fs-TRF of **Pd-B-1** and **Pd-B-2** and time constants of the fluorescence decay (*τ*_1_ = 0.3–0.4 ps for DSS and *τ*_2_ = 0.6–1 ps for ISC) are different from those of **Pd-N-1** and **Pd-G-1** (*τ*_1_ = 0.9 ps for DSS and *τ*_2_ = 12–21 ps for ISC), and other Pd(ii) complexes containing tetradentate [O^N^C^N] ligands.^9^ Thus the ISC process of the Pd(ii) complexes can be significantly tuned by the structure of the tetradentate ligand scaffold.

The fast ISC revealed from fs-TRF data and long emission lifetime as well as the unchanged emission profile observed in ns-TRE measurements with microseconds delay time lend support to the emissions from the current Pd(ii) complexes to have triplet parentage. For **Pd-B-1** there is no drastic change in the emission lifetime and *k*_r_ with increasing temperature from 77 to 300 K; thus, this complex does not show TADF properties.^18^

**Pd-B-1** exhibits blue phosphorescence accompanied with a high emission quantum yield, properties that are unprecedented for Pd(ii) complexes. Thus, the [N^C^C^N] ligand can raise the energy of Pd(ii) d–d excited states well above that of a blue–green emission. DFT/TDDFT calculations reveal that the spiro-fluorene of **Pd-B-1** plays a determinant role in the highly emissive and long-lived character of the excited state of this Pd(ii) complex. With the spiro-fluorene linkage, the T_1_ excited state of **Pd-B-1** shows little metal character, while the *O*-linkage in **Pd-B-2** favours more metal character in the T_1_ excited state. This leads to a faster radiative decay process and hence a larger *k*_r_ is found for **Pd-B-2** (*k*_r_ = 8.13 × 10^3^ s^–1^) than for **Pd-B-1** (*k*_r_ = 4.43 × 10^3^ s^–1^). However, the higher metal parentage in the T_1_ excited state of **Pd-B-2** also results in a faster SOC for the T_1_ → S_0_ non-radiative decay process than that of **Pd-B-1**. Therefore, **Pd-B-2** shows a larger *k*_nr_ (1.27 × 10^4^ s^–1^) than **Pd-B-1** (5.00 × 10^3^ s^–1^), and this can account for the lower emission quantum yield of **Pd-B-2** than that of **Pd-B-1**. Nevertheless, the Pd(ii) complexes described herein display more intense emission than many other reported Pd(ii) complexes.^9^,^11^ The rigid organic framework of tetradentate ligands helps minimize structural distortion in the excited states of the complexes, as revealed by the slight variations in the integrated area of the emission spectra of the complexes with increasing temperature. Together with the destabilization of the non-radiative d–d excited state by the strong C donor atoms of the tetradentate ligands, the Pd(ii) complexes herein show a much smaller *k*_nr_ than many other reported Pd(ii) complexes. Moreover, the emission properties of this class of Pd(ii) complexes can be readily manipulated by modifying the chemical structure of the ligand system. For example, **Pd-B-4**, which is derived from **Pd-B-1** with the replacement of a pyridine ring by a 1-isoquinoline ring, shows a remarkable red shift in the emission maximum relative to the latter (466 nm [**Pd-B-1**] *versus* 599 nm [**Pd-B-4**]). From the DFT/TDDFT analysis, the LUMO of **Pd-B-1** is mainly localized on the 2-phenylpyridine moiety of the ligand (Fig. 3). Therefore, the red shift in the emission maximum of **Pd-B-4** can be attributed to the lower LUMO level of the 1-phenylisoquinoline moiety than that of the 2-phenylpyridine moiety.

There has been a growing interest in applying organometallic photo-catalysts in organic synthesis owing to the mild reaction conditions, such as excitation wavelength in the visible region, which can be better tolerated by organic molecules containing functional groups that are sensitive to UVC radiation (100–290 nm).14b,c Employing transition metal complexes other than that of Ir(iii) and Ru(ii) for photo-reductive C–C bond formation is very rare. Notable examples include [Au_2_(μ-dppm)_2_]Cl_2_ and [Pt(R–C^N^N–R′)(C≡CArF_5_)] for reductive C–C bond formation of unactivated alkyl/aryl bromide, and [Pt(C^N)acac] for trifluoromethylation of alkenes and heteroarenes.13h,^27^ Although the use of an organic sensitizer for photocatalytic [2 + 2] cycloaddition induced by visible light has been reported,^28^ Ir(iii) sensitizers such as [Ir{dF(CF_3_)ppy}_2_(dtbbpy)]PF_6_ are advantageous as they have a high triplet energy (∼2.6 eV) and intense absorptions in the visible region. Nonetheless, there have been no examples of transition metal complexes that show both high-energy emission (*E*_t_ ∼ 2.5 eV) and long emission lifetimes on the order of 100 μs as well as with intense absorptions in the visible region. Complexes that possess all these properties are good candidates for photochemical reactions driven by visible light. In this work, **Pd-N-1**, having *E*_t_ ∼ 2.48 eV and *τ*_obs_ ∼ 272 μs, and fairly strong absorptions (*ε* ∼6 × 10^3^ dm^3^ mol^–1^ cm^–1^) at *λ*_abs_ of 400–450 nm, was found to be an efficient photo-catalyst for both C–C bond formation of unactivated alkyl bromide and [2 + 2] cycloaddition of styrene using blue LED/23 W CFL as the light source.^29^ These are the first examples of applying Pd(ii) complexes in these photo-catalytic reactions.

Fluorescence-based OLEDs (flu-OLEDs) are attractive owing to their high colour purity and long operational lifetime.^22^ However, the EQE of flu-OLEDs is limited to 5%, assuming the out-coupling efficiency of OLEDs to be 20%.^22^ Recently, there has been a surge of interest to use thermally activated delayed fluorescence (TADF) compounds as dopant; TADF-assisted fluorescence OLEDs (TAF-OLEDs) and triplet harvesting in flu-OLEDs with EQEs of 13.4–18% have been reported.^22^ In this work, Pd(ii) complexes having long-lived excited states have been used to fabricate green and yellow PSF-OLEDs with devices having high colour purity, EQE of up to 14.32% and maximum LT_90_ > 80 000 h realized. The gentle efficiency roll-off of these Pd(ii)-based PSF-OLED devices is attributed to the short emission lifetime of the fluorescent emitter as well as rapid energy transfer from the Pd(ii) complex to the fluorescent dopant. The cascade of energy transfer in these PSF-OLEDs is rather simple, involving a direct energy transfer from triplet excited state of the phosphorescent Pd(ii) complex to singlet excited state of the fluorescent dopant *via* Förster resonance energy transfer.15*a* Although the energy loss *via* the Dexter mechanism cannot be excluded as in the case of TAF-OLEDs,^18^,^22^,^30^ we envision that the device performance can be further improved by fine-tuning the device structure. Since the triplet energy of the Pd(ii) complexes can be tuned, as shown in this work, utilizing strongly phosphorescent Pd(ii) complexes in PSF-OLEDs provides a simple alternative for TAF-OLEDs.

## Conclusion

We have developed a panel of highly phosphorescent Pd(ii) complexes which display sky blue to red phosphorescence with emission lifetimes of up to 272 μs. With these Pd(ii) complexes as emitters, green and sky blue phosphorescent OLEDs have been fabricated. High EQEs of 14.45% and 16.48% have been achieved for the green and blue Pd-OLEDs by using **Pd-G-1** and **Pd-B-1** as emitters, respectively. By using these Pd(ii) complexes as sensitizers, green and yellow PSF-OLEDs with high EQEs of up to 14.32%, high colour purity and long operational lifetimes LT_90_ of more than 80 000 h have been realized. The visible light-catalysed reductive C–C bond formation of alkyl bromide using the Pd(ii) complexes as the catalysts has been investigated with conversions and yields of up to 90% and 83%, respectively. The [2 + 2] cycloaddition of styrenes catalysed by energy transfer from Pd(ii) complexes in the excited state has been explored, with conversions and yields comparable to those reported for Ir(iii) complexes.

## Supplementary Material

Supplementary information

Crystal structure data
