# Supplementary material for: Highly luminescent palladium(ii) complexes with sub-millisecond blue to green phosphorescent excited states. Photocatalysis and highly efficient PSF-OLEDs
Source: Chem Sci. 2016 Jun 15;7(9):6083–98. doi: 10.1039/c6sc00462h (PMC6024183; doi:10.1039/c6sc00462h)
Supplement: Supplementary file 1 [file SC-007-C6SC00462H-s001.pdf]

Electronic Supplementary Material (ESI) for Chemical Science.
This journal is © The Royal Society of Chemistry 2016

Electronic Supplementary Information

Highly luminescent palladium(II) complexes with sub-millisecond blue to green phosphorescent excited states. Photocatalysis and highly efficient PSF-OLEDs

Pui-Keong Chow,[a] Gang Cheng,[ab] Glenna So Ming Tong,[a] Chensheng Ma,[c] Wai-Ming Kwok, [d] Wai-Hung Ang,[a] Clive Yik-Sham Chung,[a] Chen Yang,[a] Feng Wang[a] and Chi-Ming Che*[ab]

[a] State Key Laboratory of Synthetic Chemistry, Institute of Molecular Functional Materials, HKU-CAS Joint Laboratory on New Materials and Department of Chemistry, The University of Hong Kong, Pokfulam Road, Hong Kong, China. E-mail: cmche@hku.hk
[b] HKU Shenzhen Institute of Research and Innovation, Shenzhen 518053, China.
[c] School of chemistry and Chemical Engineering, Shenzhen University, Shenzhen 518060, China.
[d] Department of Applied Biology and Chemical Technology, The Hong Kong Polytechnic University, Hung Hom, Kowloon, Hong Kong SAR, China.

Table of Contents

Synthesis and characterization	Page 2
Steady-state emission and lifetime measurements	3
Time-resolved spectroscopy	4
OLEDs fabrications and characterization	4
Computational details	5
General procedure for visible-light-induced reductive C–C bond formation	6
General procedure for [2+2] cycloaddition of styrenes	6
Scheme for the syntheses of starting materials and ligands of **Pd-B-1, Pd-B-3, Pd-B-4 and Pd-N-1**	28
Scheme for the syntheses of metal complexes	36
General procedures for the syntheses of starting materials	28
General procedures for the syntheses of ligands	34
General procedures for the syntheses of complexes **Pd-B-1**to **Pd-B-4**, **Pd-G-1** and **Pd-N-1**	36
Further details on energy transfer processes	50
Example of NMR analysis of reaction mixture of photochemical reaction	42
References	51
**Table S1** Selected bond angles and distances of **Pd-B-3**	8
**Table S2** Selected bond angles and distances of **Pd-B-1**	8
**Table S3** Crystal data of **Pd-B-1** and **Pd-B-3**	9
**Table S4** Huang-Rhys factor for the normal modes with frequencies in the range $1500 < \omega_I \leq 1700$ cm^{-1} and the estimated non-radiative decay rates k_{nr} of the lowest energy triplet excited state for the complexes **Pd-B-1**, **Pd-B-2**, and **Pd-N-1**	19
**Table S5** Reason for the absence of charge transfer reactions between substrate **E₁** and **Pd-B-1**/**Pd-N-1** in excited state	24
**Tables S6–S11** Cartesian coordinates of the optimized geometries of **Pd-B-1**, **Pd-B-2** and **Pd-N-1**	43
**Fig. S1** ^{1}H NMR of **Pd-B-1** at 273 K to 323 K in $CDCl_3$ (400 MHz)	7
**Fig. S2** Thermograms of **Pd-B-1**, **Pd-B-2**, **Pd-B-4**, **Pd-G-1**, **Pd-G-2** and **Pd-N-1**	7
**Fig. S3** X-ray crystal structure of **Pd-B-3**	8
**Fig. S4** Cyclic voltammograms of **Pd-B-2**, **Pd-B-4**, **Pd-G-1**, **Pd-G-2** in DMF (0.1 mol dm^{-3} nBu$_4$NPF$_6$ as supporting electrolyte) at 298 K. Scan rate: 100 mV s^{-1}	10
**Fig S5** HOMO and LUMO surfaces for **Pd-B-1**, **Pd-B-2**, **Pd-B-4**, **Pd-N-1**, and **Pd-G-1**	10
**Fig. S6** Cyclic voltammograms of **Pd-B-1** and **Pd-B-2** in DMF (0.1 mol dm^{-3} nBu$_4$NPF$_6$ as supporting electrolyte) at 298 K	12
**Fig. S7** UV/Vis absorption spectra of **Pd-B-1**, **Pd-B-2**, and ligand of **Pd-B-1** in CH_2Cl_2 at room temperature	12
**Fig. S8** UV/Vis absorption spectra of **Pd-G-1**, **Pd-G-2**, and ligand of **Pd-G-2** in CH_2Cl_2 at room temperature	13

**Fig. S9** UV/Vis absorption spectra of **Pd-B-1** in various solvents (4×10^{-5} mol dm^{-3}) at room temperature	13
**Fig. S10** UV/Vis absorption spectra of **Pd-G-2** in various solvents (5×10^{-5} mol dm^{-3}) at room temperature	14
**Fig. S11** Emission spectra of **Pd-B-1** in various solvents (4×10^{-5} mol dm^{-3}) at room temperature	14
**Fig. S12** Emission spectra of **Pd-G-2** in various solvents (5×10^{-5} mol dm^{-3}) at room temperature	15
**Fig. S13** Temperature-dependent emission spectra of **Pd-B-1** in toluene (5×10^{-5} mol dm^{-3})	15
**Fig. S14** Plots of τ_{obs} against temperature of **Pd-B-1** in the solid state and in PMMA (5% dopant concentration)	16
**Fig. S15** Relevant MO surfaces for the T_1 excited state of the **Pd-B-2**	17
**Fig. S16** Relevant MO surfaces for the T_1 excited state of the **Pd-B-1**	18
**Fig. S17** Relevant MO surfaces for the T_1 excited state of the **Pd-N-1**	19
**Fig. S18** Temporal evolution of fs-TRF and experimental and fitted kinetic decays of fs-TRF obtained for **Pd-N-1** and **Pd-G-1** in CH_2Cl_2 with excitation at 300 nm wavelength	20
**Fig. S19** Temporal evolution of fs-TRF and experimental and fitted kinetic decays of fs-TRF obtained for **Pd-B-1** and **Pd-B-2** in CH_2Cl_2 with excitation at 300 nm wavelength	20
**Fig. S20** nsTRE and ns-TA spectra of **Pd-B-1** in degassed CH_2C_2 (5×10^{-5} mol dm^{-3}) at room temperature.	21
**Fig. S21** Time resolved absorption difference spectra of **Pd-B-1** (5×10^{-5} mol dm^{-3}) in degassed CH_3CN monitored at 0–100 μs; Time resolved absorption difference spectra of **Pd-B-1** (5×10^{-5} mol dm^{-3}) in non-degassed CH_3CN monitored at 0–400 ns	21
**Fig. S22** Time resolved absorption difference spectra of **Pd-B-1** (5×10^{-5} mol dm^{-3}) and TMEDA (0.69 mol dm^{-3}) in degassed CH_3CN monitored at 0–100 μs. Inset shows the decay of emission at 466 nm (black) and TA at 398 nm (red)	22
**Fig. S23** Stern-Volmer plot of the quenching of **Pd-B-1** at 466 nm by MV^{2+}	22
**Fig. S24** Time resolved absorption difference spectra of **Pd-B-1** (5×10^{-5} mol dm^{-3}) and $MV(PF_6)_2$ (2.5×10^{-4} mol dm^{-3}) in CH_3CN monitored at 0–2 μs. Insets show the decays of emission at 466 nm (black), TA at 436 nm (red) and TA at 398 (blue)	23
**Fig. S25** Time-resolved absorption difference spectra of **Pd-N-1** (5×10^{-5} mol dm^{-3}) in degassed CH_3CN monitored at 0–80 μs; time-resolved absorption difference spectra of **Pd-N-1** (5×10^{-5} mol dm^{-3}) and *i*PrNEt (0.01 mol dm^{-3}) in degassed CH_3CN monitored at 0–80 μs; time-resolved absorption difference spectra of **Pd-N-1** (5×10^{-5} mol dm^{-3}) and substrate $\mathbf{A_1}$ (5×10^{-3} mol dm^{-3}) in degassed CH_3CN monitored at 0–80 μs	23
**Fig. S26** Stern-Volmer plot of quenching of emission of **Pd-B-1** and **Pd-N-1** by substrate $\mathbf{E_1}$	24
**Fig. S27** Normalized EL spectra and external quantum efficiency-luminance characteristics of OLEDs with **Pd-G-1** at the dopant concentrations of 4, 10 and 20 wt%	25
**Fig. S28** Normalized EL spectra and external quantum efficiency-luminance characteristics of OLEDs with **Pd-G-2** at the dopant concentrations of 4, 10 and 20 wt%	25
**Fig. S29** Normalized EL spectra and external quantum efficiency-luminance characteristics of OLEDs with **Pd-B-1** at the dopant concentrations of 2, 6 and 10 wt%	26
**Fig. S30** Normalized EL spectra and external quantum efficiency-luminance characteristics of OLEDs with **Pd-B-2** at the dopant concentrations of 2, 6 and 10 wt%.	26
**Fig. S31** Device lifetime (T_{90}) of the OLED with **Pd-G-1** as emitter as well as PSF-OLED with **Pd-G-1** as phophorescent sensitizer and TBRb as fluorescent emitter	27
**Fig. S32–S37** ^{1}H NMR spectra of **Pd-B-1** to **Pd-B-4**, **Pd-G-1** and **Pd-N-1**	37

Synthesis and characterization

Palladium(II) acetate and the starting materials for the synthesis of ligands (purchased from Sigma-Aldrich) were used as received. Unless specified, solvents of analytical grade were used in the syntheses. THF and toluene were distilled over sodium/benzophenone under a nitrogen atmosphere prior to use.

^{1}H NMR spectroscopic experiments were performed with a Bruker Avance 500, 400, 600 or DPX-300 FT-NMR spectrometer. ^{19}F NMR spectroscopic experiments were performed with a Bruker Avance 400 FT-NMR spectrometer. Chemical shifts [ppm] reported were calibrated to the solvent residual peak(s) or tetramethylsilane (TMS) as a internal reference. Peak assignments were based on ^{1}H, ^{1}H COSY and NOESY 2D NMR spectroscopic experiments. EI mass spectra were recorded with a Finnigan MAT 95 mass spectrometer. Elemental analyses of the complexes were performed at the Institute of Chemistry of the Chinese Academy of Sciences, Beijing. UV/Vis absorption spectra were recorded with a Hewlett-Packard 8453 diode spectrophotometer. X-ray diffraction data of single crystals were collected with a Bruker X8 Proteum diffractometer. The diffraction images were interpreted and the diffraction intensities were integrated by using the program SAINT. The crystal structure was solved by direct methods employing the SHELXS-97 program.[1] Cyclic voltammetric measurements were performed with a Princeton Applied Research electrochemical analyzer (potentiostat/galvanostat Model 273A) with a conventional three-compartmental cell. $^{n}Bu_4NPF_6$ (0.1 mol dm^{-3}) in DMF was used as a supporting electrolyte for the electrochemical measurements at room temperature. All solutions used in electrochemical measurements were deaerated with pre-purified argon gas. Ag/$AgNO_3$ (0.1 mol dm^{-3} in acetonitrile), a glassy carbon electrode, and a platinum wire were used as reference electrode, working electrode, and counter electrode, respectively. Ferrocene was used as an internal reference.

Steady-state emission and lifetime measurements

Steady-state excitation and emission spectra were obtained with a SPEX Fluorolog-3 spectrophotometer. All solutions for photophysical measurements were degassed with no less than 5 successive freeze-pump-thaw cycles prior to the measurements. The emission spectra were corrected for monochromator and photomultiplier efficiency and for xenon-lamp stability. Emission lifetime measurements were carried out with a Quanta Ray GCR 150-10 pulsed Nd:YAG laser system (pulse output: 355 nm, 5–6 ns). Luminescence quantum yields were measured by the optical dilution method with $[Ru(bpy)_3](PF_6)_2$ (bpy = 2,2'-bipyridine) in degassed acetonitrile (Φ_{em} = 0.062) or BPEA (9,10-bis(phenylethynyl)anthracene) in degassed benzene (Φ_{em}= 0.85) as a

standard and calculated by: $\Phi_s = \Phi_r(B_r/B_s)(n_s/n_r)_2(D_s/D_r)$, in which the subscripts s and r refer to sample and reference standard solution, respectively, n is the refractive index of the solvents, D is the integrated intensity, and Φ_{em} is luminescence quantum yield. The refractive indices of the solvents at room temperature were taken from standard sources. Errors for Φ_{em} values (± 10%) were estimated. Thin film samples for photoluminescence (PL) spectra and absolute quantum yield (QY) measurement were fabricated by drop casting PMMA-complex solutions (PMMA to complex ratio is 95:5; chlorobenzene to solute ratio is 95:5) on quartz plates. PL spectra and absolute QY were recorded on a Horiba Jobinyvon Fluorolog-3 fluorescence spectrofluorometer equipped an integrating sphere model: F-3018.

Time-resolved spectroscopy

Femtosecond time-resolved fluorescence (fs-TRF) measurements were performed based on a commercial Ti:sapphire regenerative amplifier laser system (800 nm, 40 fs, 1 KHz, and 3.5 mJ/pulse). The pump pulse with wavelength at 355, 380, 400 nm was produced from TOPAS-C pumped by the 800 nm fundamental laser. The fs-TRF was achieved by employing the Kerr-gate technique.[2] A Kerr device, consisting of a 1 mm-thick Kerr medium (benzene contained in a quartz cell) equipped within a crossed polarizer pair, was driven by the 800 nm laser to function as an ultrafast optical shutter to sample transient fluorescence spectra at various selected pump/probe delays. The temporal delay of probe to pump pulse was varied by a computer controlled optical delay line. The fs-TRF signals were collected by a monochromator and detected with a liquid-nitrogen cooled CCD detector. The instrument response function (IRF) of fs-TRF is wavelength-dependent. As the detection wavelength varies from 600 to 280 nm, the IRF varies from ~0.5 to ~2 ps. All the time-resolved measurements were done at room temperature and atmospheric pressure with ~15 mL samples flowed into a cell with 0.5 mm path length to avoid photo-degradation. The sample solutions were monitored by UV/Vis absorption spectroscopy and revealed no degradation after the fs time-resolved experiments.

The nanosecond time-resolved emission spectra (ns-TRE) and nanosecond time-resolved absorption difference spectra (ns-TA) were performed with a LP920-KS Laser Flash Photolysis Spectrometer (Edinburgh Instruments LTD., Livingston, UK). The excitation source was the 355 nm output (third harmonic) of an Nd:YAG laser (Spectra-Physics Quanta-Ray Lab-130 Pulsed Nd:YAG Laser). The signals were processed by a PC plugin controlled with L900 software. The preparation of samples for the measurements was the same as those for steady-state emission measurements.

OLEDs fabrications and characterization
(a) Materials

TAPC (4,4'-cyclohexylidenebis[*N,N*-bis(4-methylphenyl)benzamine]),
NPB (*N,N*' -bis(naphthalen-1-yl)-*N,N*'-bis(phenyl)-benzidinemCP (1,3-bis(carbazol-9-yl)benzeneCzSi (9-(4-*tert*-butylphenyl)-3,6-bis(triphenylsilyl)-9*H*-carbazolemCBP (3,3-di(9*H*-carbazol-9-yl)biphenyl),
BAlq (bis(2-methyl-8-quinolinolate)-4- (phenylphenolato)aluminiumAlq (tris(8-hydroxyquinolinato)aluminum),
TmPyPB (3,3'-[5'-[3-(3-pyridinyl)phenyl] [1,1':3',1''-terphenyl]-3,3''-diyl] bispyridine),
TBRb (2,8-Di-*tert*-butyl-5,11-bis(4-*tert*-butylphenyl)-6,12-diphenyltetracene),
TTPA (9,10-bis[*N,N*-di-(p-tolyl)-amino]anthracene)
TCTA (tris(4-carbazoyl-9-ylphenyl)amine),
TSPO1 (diphenyl-4-triphenylsilyl phenyl-phosphineoxide)
were purchased from Luminescence Technology Corp. MoO_3 and LiF were purchased from Sigma-Aldrich. All of these materials were used as received.

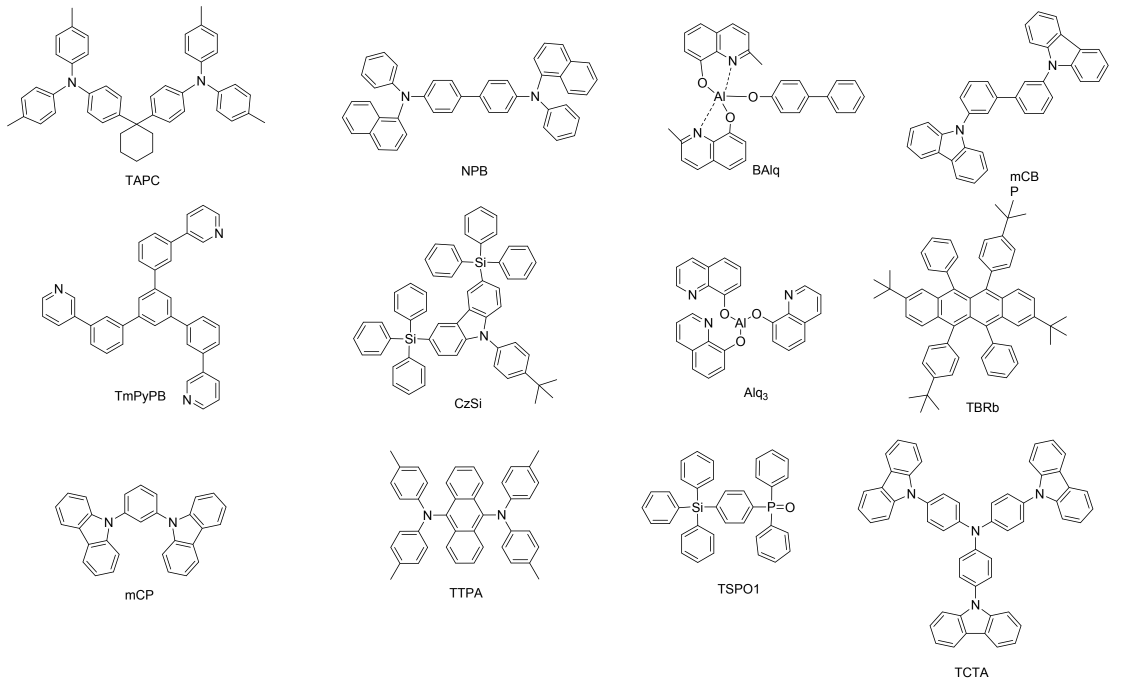

(b) Device fabrication and characterization

Pre-patterned ITO-glass substrates were cleaned in an ultrasonic bath of Decon 90 detergent and deionized water, rinsed with deionized water, and then cleaned in sequential ultrasonic baths of deionized water, acetone, and *iso*-propanol, and subsequently dried in an oven for 1 hour. OLEDs were fabricated in a Kurt J. Lesker SPECTROS vacuum deposition system with a base pressure of 10^{-8} mBar. In the vacuum chamber, MoO_3, organic materials, LiF and Al were thermally deposited. Film thicknesses were determined *in-situ* by calibrated oscillating quartz-crystal

sensors. The Commission Internationale de L'Eclairage (CIE) coordinates, luminance-current density-voltage characteristics (*L*-*J*-*V*), and electroluminescence (EL) spectra were measured simultaneously with a programmable Keithley model 2400 source-meter measurement unit and a Photoresearch PR-655 spectrascan spectroradiometer. All devices were characterized at room temperature without encapsulation except for those for device stability investigation. UV-curable sealant, cover glass and desiccant were used to encapsulate the OLEDs for stability investigation. External quantum efficiency and power efficiency were calculated by assuming a Lambertian distribution.

Computational details

In this work, the hybrid density functional, PBE0,[3] was employed for all calculations using the program package G09.[4] The 6-31G* basis set[5] is used for all atoms except Pd, which is described by the Stuttgart relativistic pseudopotential and its accompanying basis set (ECP28MWB).[6] Solvent effect was also included by means of the polarizable continuum model (PCM).[7] The solvent employed is dichloromethane. Geometry optimizations of the singlet ground state (S_0) were carried out using the density functional theory without symmetry constraints. For the triplet excited state, both unrestricted DFT (UDFT) and time-dependent DFT (TDDFT) were employed to locate the minimum energy triplet excited states. Frequency calculations were performed on the optimized structures to ensure that they are minimum energy structures by the absence of imaginary frequency (i.e. NImag = 0). Stability calculations were also performed for all the optimized structures to ensure that all the wavefunctions obtained are stable. Details of the Huang-Rhys factors (*S*) and non-radiative rate calculations were reported elsewhere.[8]

As UDFT and TDDFT are two different methods, the minimum energy structures were located by comparing the energies of the triplet excited states calculated using the state-specific SS-PCM TDDFT approach.[9]

General procedure for visible-light-induced reductive C–C bond formation

Alkyl bromide (~0.02–0.025 g), **Pd-N-1**/**Pd-B-1** (1 mol%) and diisopropylethylamine ($^i Pr_2NEt$) were dissolved in acetonitrile (2.3 mL). The reaction mixture was prepared in a test tube (24 mm internal diameter, 170 mm tall) equipped with a magnetic stirrer and a rubber septum. The solution was degassed by bubbling nitrogen gas through the solution for 15 min in the dark. A 2.5 W blue LED (λ_{em} = 420–520 nm, λ_{max} = 462 nm)/150 W Xenon Lamp was used as the light source. After irradiation of 10–20 h, the solvent was removed under reduced pressure. All the conversions and product

yields were calculated on the basis of ^{1}H NMR spectroscopic analysis using 4,4'-dimethyl-2,2'-bipyridine as an internal standard.

General procedure for [2+2] cycloaddition of styrenes

Styrene (0.01 mol dm^{-3}) and **Pd-N-1/Pd-B-1**/*fac*-Ir(ppy)$_3$ **(**1 mol%) were dissolved in acetonitrile (5 mL). The reaction mixture was prepared in a test tube (24 mm internal diameter, 170 mm tall) equipped with a magnetic stirrer and a rubber septum. The solution was degassed by bubbling nitrogen gas through the solution for 15 min in the dark. A 2.5 W blue LED (λ_{em} = 420–520 nm, λ_{max} = 462 nm)/150 W Xenon Lamp/23 W compact fluorescent lamp (1450 lm, 23 lm/W, Philips) was used as the light source. After irradiation of 3–24 h, the solvent was removed under reduced pressure. All the conversions and product yields were calculated on the basis of ^{1}H NMR spectroscopic analysis using 4,4'-dimethyl-2,2'-bipyridine as an internal standard.

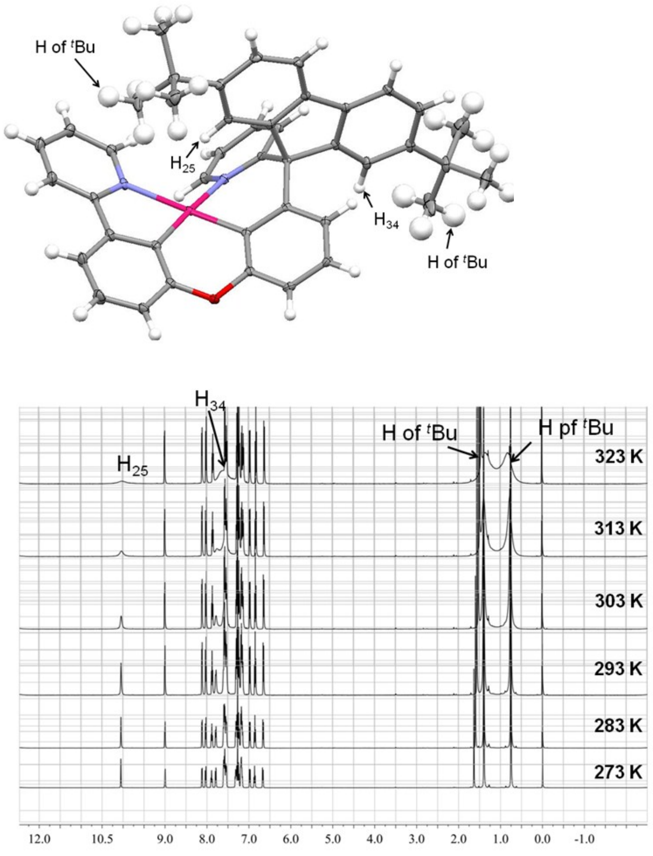

Fig. S1 ^{1}H NMR of **Pd-B-1** at 273 K to 323 K in CDCl$_3$ (400 MHz).

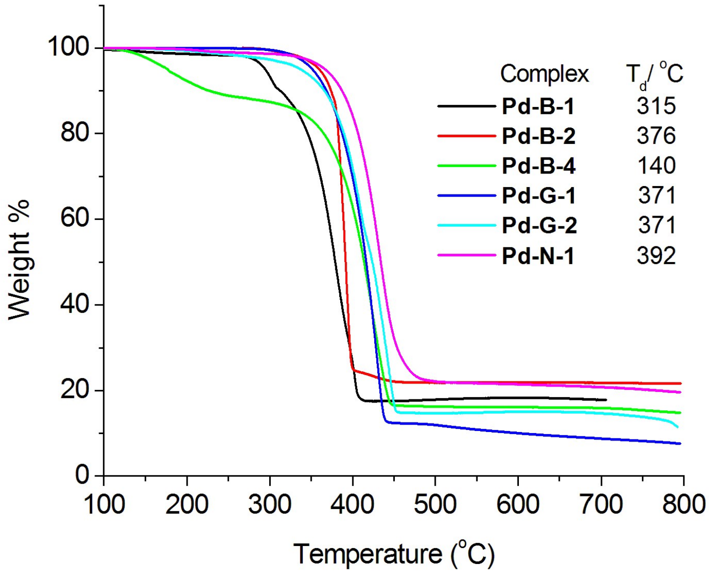

Fig. S2 Thermograms of **Pd-B-1**, **Pd-B-2**, **Pd-B-4**, **Pd-G-1**, **Pd-G-2** and **Pd-N-1**.

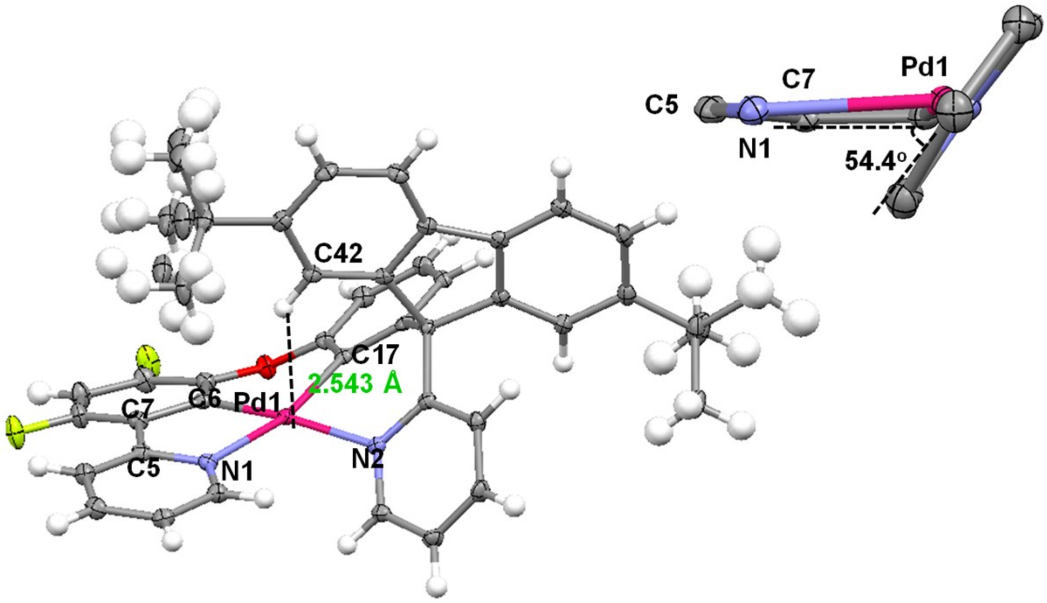

Fig. S3 X-ray crystal structure of **Pd-B-3**. Thermal ellipsoids are drawn at the 35% probability level (note: the distance between the hydrogen atom residing on C42 and the normal plane of the Pd1-C6-C7-C5-N1 chelating ring is 2.543 Å); the inset depicts the angle between the pyridine ring and the aforementioned chelating ring.

Table S1 Selected bond angles and distances of **Pd-B-3**

Pd(1)–N(2)	2.0968(19) Å	Pd(1)–N(1)	2.1386(18) Å
Pd(1)–C(6)	1.964(2) Å	Pd(1)–C(17)	1.985(2) Å
C(6)-Pd(1)-N(2)	172.67(8) °	C(17)-Pd(1)-N(1)	166.77(8) °
C(6)-Pd(1)-N(1)	80.10(9) °	C(6)-Pd(1)-C(17)	91.51(10) °
C(17)-Pd(1)-N(2)	88.97(8) °	N(2)-Pd(1)-N(1)	100.66(7) °

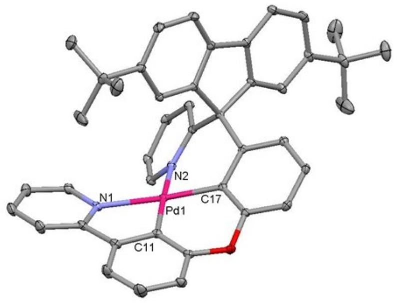

Table S2 Selected bond angles and distances of **Pd-B-1**

Pd(1)–N(2)	2.0968(19) Å	Pd(1)–N(1)	2.1441(18) Å
Pd(1)–C(11)	1.958(2) Å	Pd(1)–C(17)	1.982(2) Å
C(11)-Pd(1)-N(2)	173.74(8)^o	C(17)-Pd(1)-N(1)	165.96(7) ^{o}
C(11)-Pd(1)-N(1)	80.51(8) ^{o}	C(11)-Pd(1)-C(17)	90.39(9) ^{o}
C(17)-Pd(1)-N(2)	90.27(8) ^{o}	N(2)-Pd(1)-N(1)	99.95(7) ^{o}

Table S3 Crystal data of **Pd-B-1** and **Pd-B-3**

Complex	**Pd-B-1**	**Pd-B-3**
Empirical formula	$C_{43}H_{38}N_2O_1Pd{\cdot}C_4H_8O_2$	$C_{43}H_{36}F_2N_2OPd$
Formula weight	793.26	741.14
Temperature [K]	100	100
Wavelength [Å]	1.54178	1.54178
Crystal system	Monoclinic	Monoclinic
Space group	P 1 21/c 1	$P2_{1/n}$
a [Å]	19.469(3)	15.2388(13)
b [Å]	12.2196(16)	14.5866(13)
c [Å]	16.391(2)	15.9868(14)
α [^{o}]	90	90
β [^{o}]	102.099(3)	106.4424(18)
γ [^{o}]	90	90
Volume [$Å^3$]	3813.0(9)	3408.3(5)
Z	4	4
Density (calcd) [g cm^{-3}]	1.382	1.444
Crystal size [mm^3]	0.4 × 0.3 × 0.3	0.1 × 0.1 × 0.1
μ [mm^{-1}]	4.271	4.781
F(000)	1648	1520.0
$2\theta_{max}$[^{o}]	133.88	134.214
Index ranges	–23≦h≦22<br>–14≦k≦10	–18≦h≦18<br>–17≦k≦17

	–16≦1≦19	–18≦1≦19
No. of unique data	6652	6015
No. obsd. Data for I > 2σ(*l*)	6622	5863
No. variables	486	464
R_1	0.0323	0.0379
wR_2	0.0885	0.1013
Goodness of fit	1.043	1.038
Residual ρ [e Å^{-3}]	+1.056, –0.565	+0.447, –0.996
CCDC number	CCDC 1048773	CCDC 1449190

Note: $R_1 = \Sigma||F_o| - |F_c||/\Sigma|F_o|$, $wR_2 = \{\Sigma[w(F_o^2 - F_c^2)^2]/\Sigma[w(F_o^2)^2]\}^{1/2}$

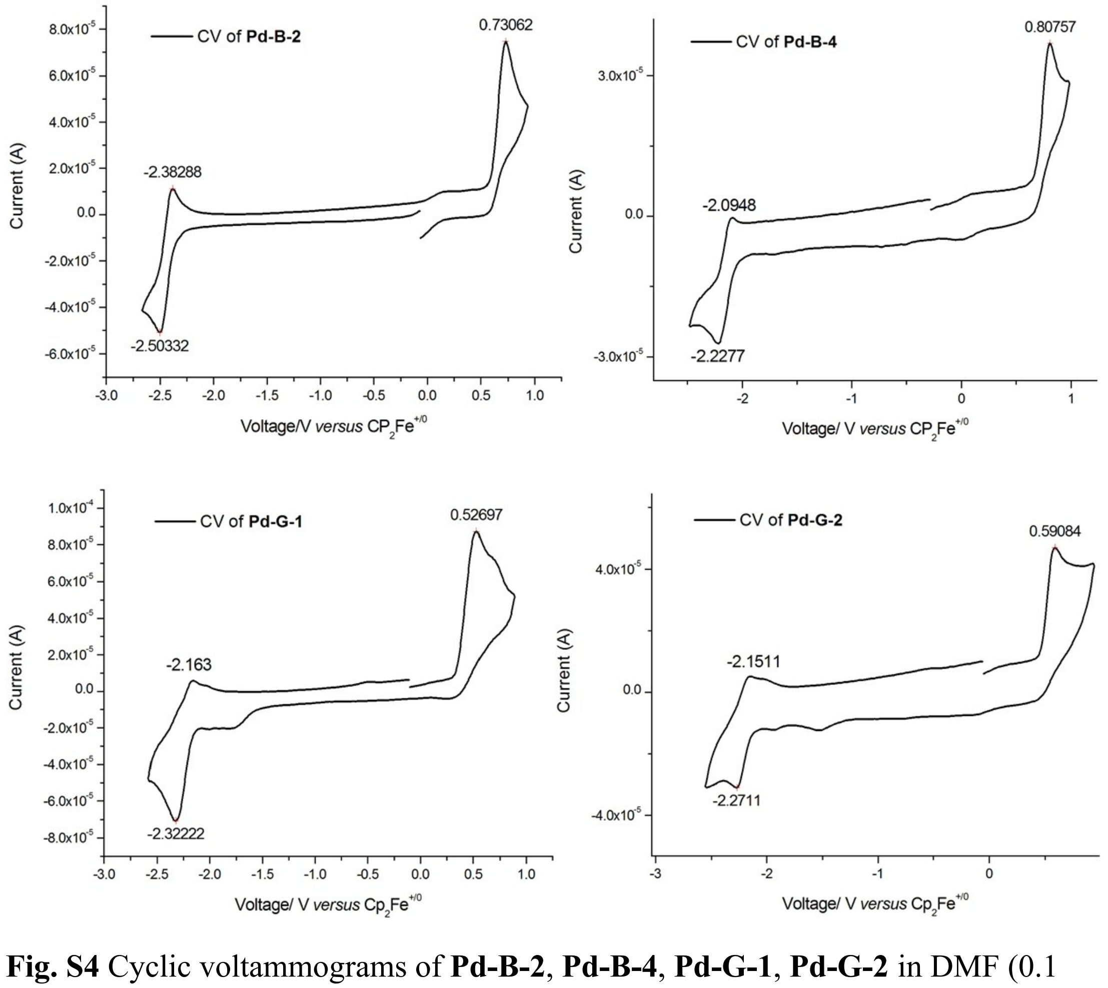

Fig. S4 Cyclic voltammograms of **Pd-B-2**, **Pd-B-4**, **Pd-G-1**, **Pd-G-2** in DMF (0.1 mol dm^{-3} $^{n}Bu_4NPF_6$ as supporting electrolyte) at 298 K. Scan rate: 100 mV s^{-1}.

Fig. S5 HOMO and LUMO surfaces for **Pd-B-1**, **Pd-B-2**, **Pd-B-4**, **Pd-N-1**, and **Pd-G-1**[a]

	**HOMO**	**LUMO**

**Pd-G-1**	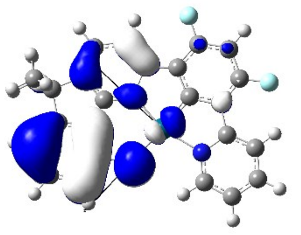 -5.51 eV (4%)	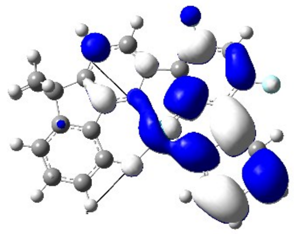 -1.74 eV (7%)
**Pd-N-1**	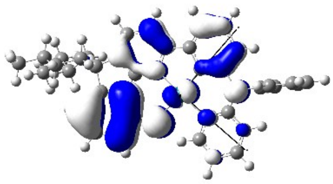 -5.34eV (8%)	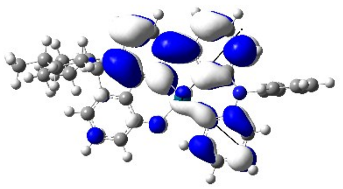 -1.48eV (4%)
**Pd-B-1**	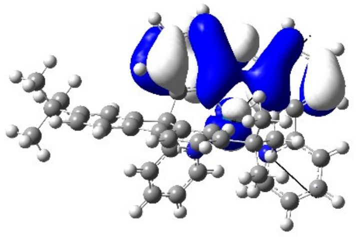 -5.57eV (15%)	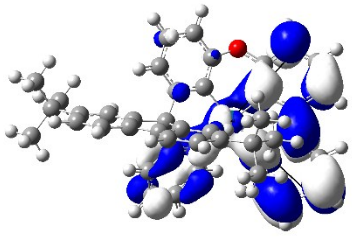 -1.51eV (5%)
**Pd-B-2**	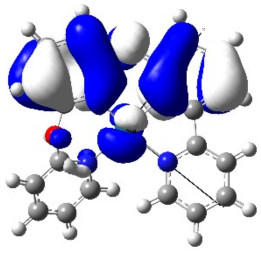 -5.65eV (15%)	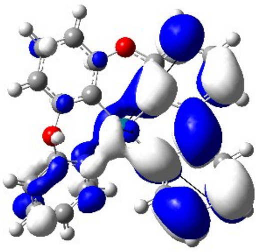 -1.55eV (5%)
**Pd-B-4**	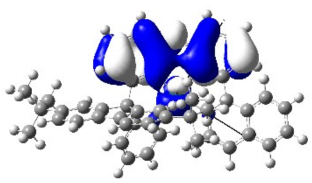 -5.58eV (16%)	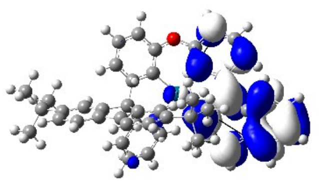 -1.94eV (3%)

[a] orbital energies are also included with the % of metal character in the parentheses.

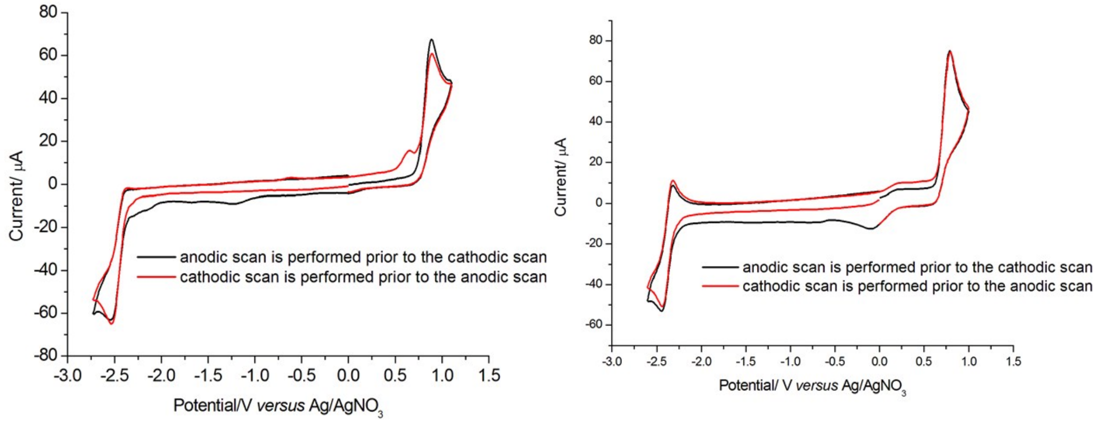

Fig. S6 Left: Cyclic voltammograms of **Pd-B-1** in DMF (0.1 mol dm^{-3} $^{n}Bu_4NPF_6$ as supporting electrolyte) at 298 K. Scan rate: 100 mV s^{-1}. Right: Cyclic voltammograms of **Pd-B-2** in DMF (0.1 mol dm^{-3} $^{n}Bu_4NPF_6$ as supporting electrolyte) at 298 K. Scan rate: 100 mV s^{-1}

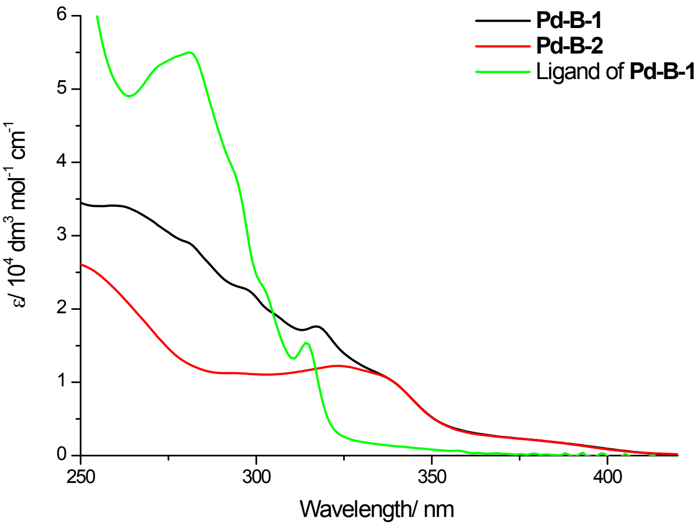

Fig. S7 UV/Vis absorption spectra of **Pd-B-1**, **Pd-B-2**, and ligand of **Pd-B-1** in CH_2Cl_2 at room temperature.

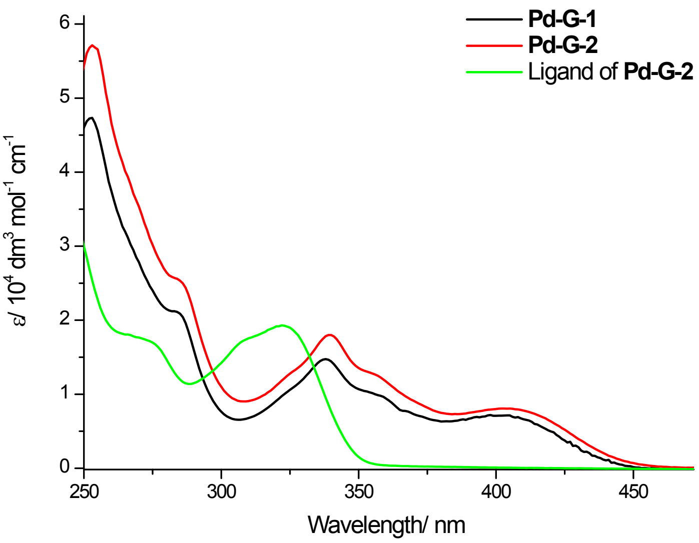

Fig. S8 UV/Vis absorption spectra of **Pd-G-1**, **Pd-G-2**, and ligand of **Pd-G-2** in CH_2Cl_2 at room temperature.

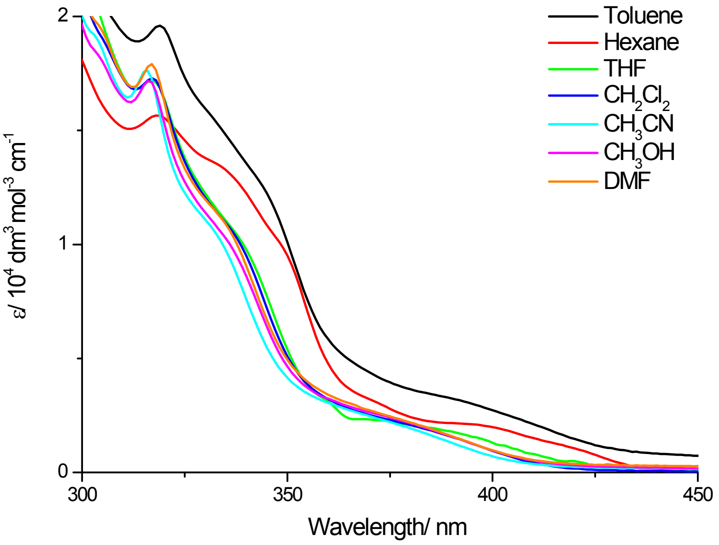

Fig. S9 UV/Vis absorption spectra of **Pd-B-1** in various solvents (4×10^{-5} mol dm^{-3}) at room temperature.

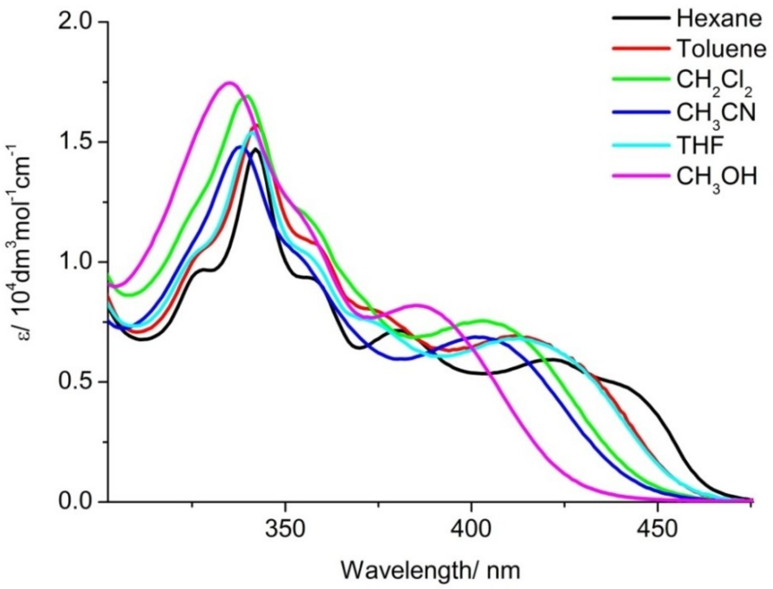

Fig. S10 UV/Vis absorption spectra of **Pd-G-2** in various solvents (5×10^{-5} mol dm^{-3}) at room temperature.[10]

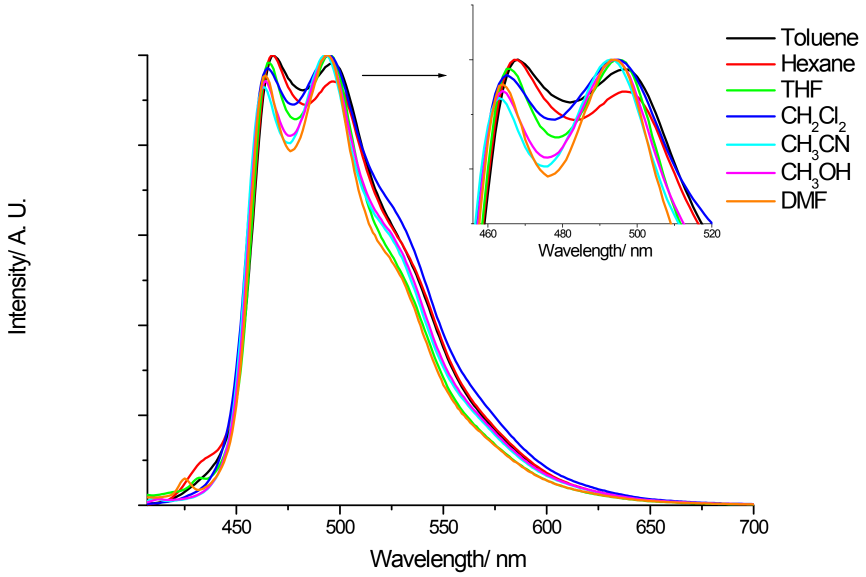

Fig. S11 Emission spectra of **Pd-B-1** in various solvents (4×10^{-5} mol dm^{-3}) at room temperature.

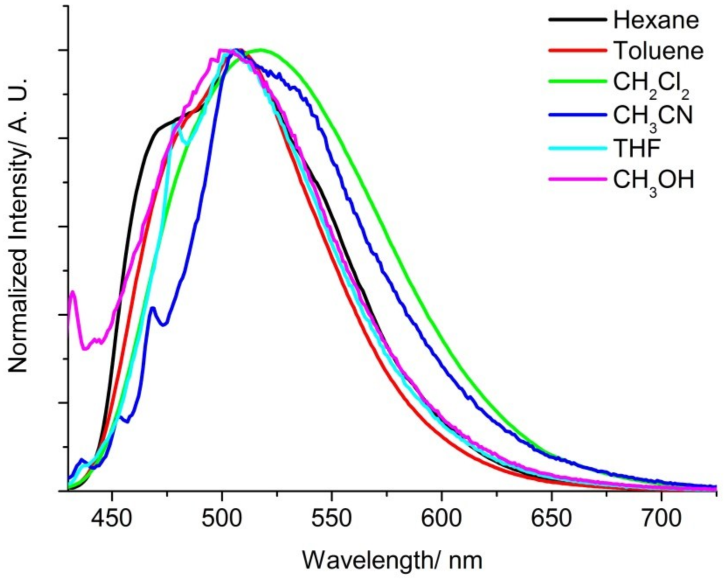

Fig. S12 Emission spectra of **Pd-G-2** in various solvents (5×10^{-5} mol dm^{-3}) at room temperature.[10]

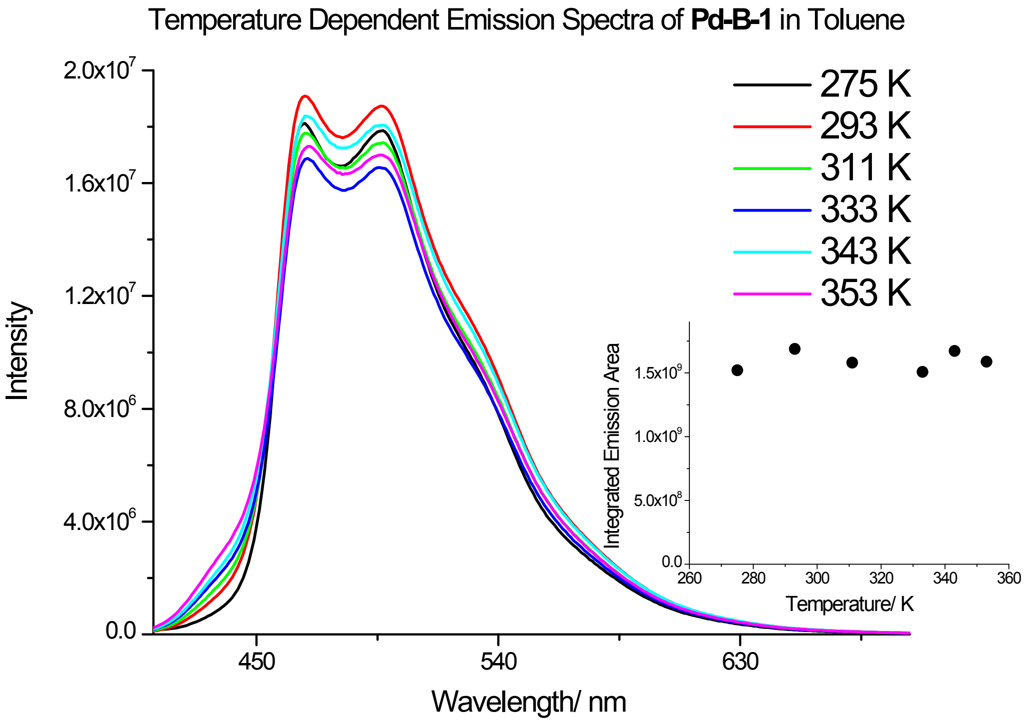

Fig. S13 Temperature-dependent emission spectra of **Pd-B-1** in toluene (5×10^{-5} mol dm^{-3}) (inset: the plot of integrated emission area versus temperature).

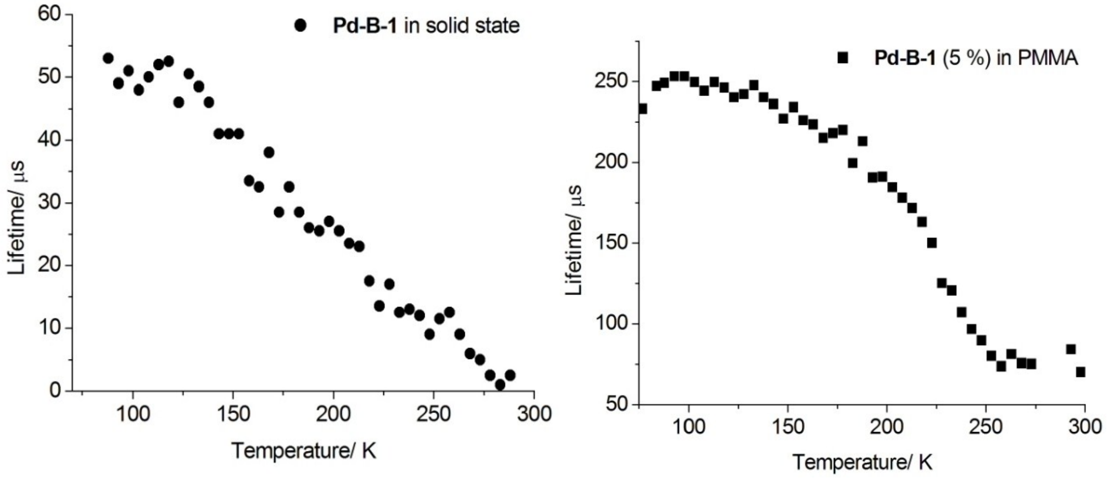

Fig. S14 Plots of τ_{obs} against temperature of **Pd-B-1** in the solid state (left) and in PMMA (5% dopant concentration) (right).

MO	Pd	Phenyl ring of N^C	Pyridine ring of N^C
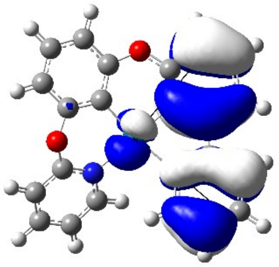 H−1	13	53	33
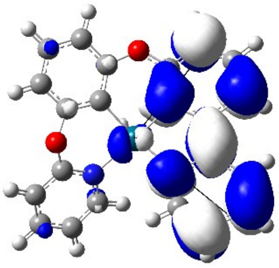 LUMO	5	38	53

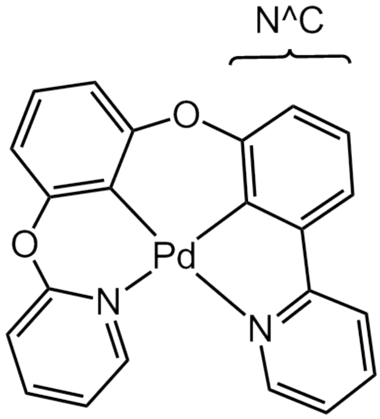

Fig. S15 Relevant MO surfaces for the T_1 excited state of the **Pd-B-2**. Top: H−1; bottom: LUMO. The values are the % contribution of the moiety to the MO.

MO	Pd	Phenyl ring of N^C	Pyridine ring of N^C	Phenyl ring of N-Flu-C	Spiro-flurene moiety
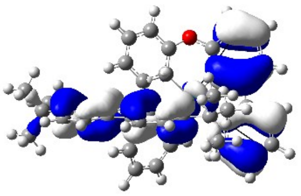 , H−2	7	33	19	1	39
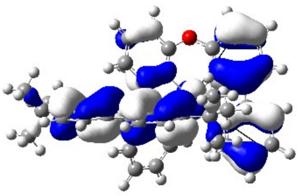 H−1	8	21	13	7	51
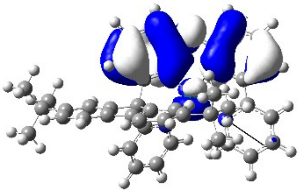 HOMO	12	30	1	38	1
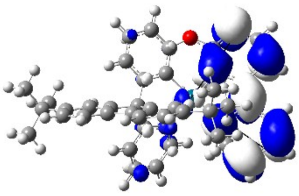 LUMO	4	38	53	1	0

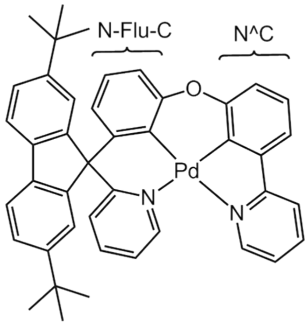

Fig. S16 Relevant MO surfaces for the T_1 excited state of the **Pd-B-1**. From top to bottom: H−2, H−1, HOMO, and LUMO. The values are the % contribution of the moiety to the MO.

MO	Pd	Py-A	Ph	Py-B	O-Ph	N-Ph
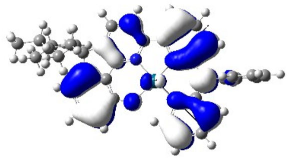 H−2	2	19	30	8	25	16
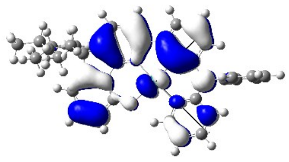 H−1	15	7	28	24	18	7
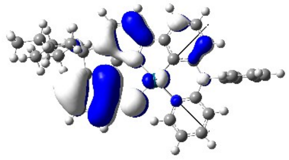 HOMO	3	1	5	14	75	1
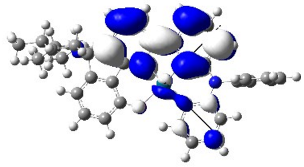 LUMO	4	6	38	49	2	0

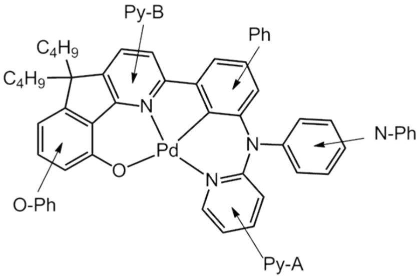

Fig. S17 Relevant MO surfaces for the T_1 excited state of the **Pd-N-1** complex. From top to bottom: H−2, H−1, HOMO, and LUMO. The values are the % contribution of the moiety to the MO.

Table S4 Huang-Rhys factor for the normal modes with frequencies in the range 1500 < $\omega_i \le 1700$ cm^{-1} and the estimated non-radiative decay rates k_{nr} of the lowest energy triplet excited state for the complexes **Pd-B-1**, **Pd-B-2**, and **Pd-N-1**

	**Pd-B-1**	**Pd-B-2**	**Pd-N-1**
ΣS_i	0.98	1.02	0.89
k_{nr} / s^{-1}	6.38×10^3	1.42×10^4	1.77×10^3

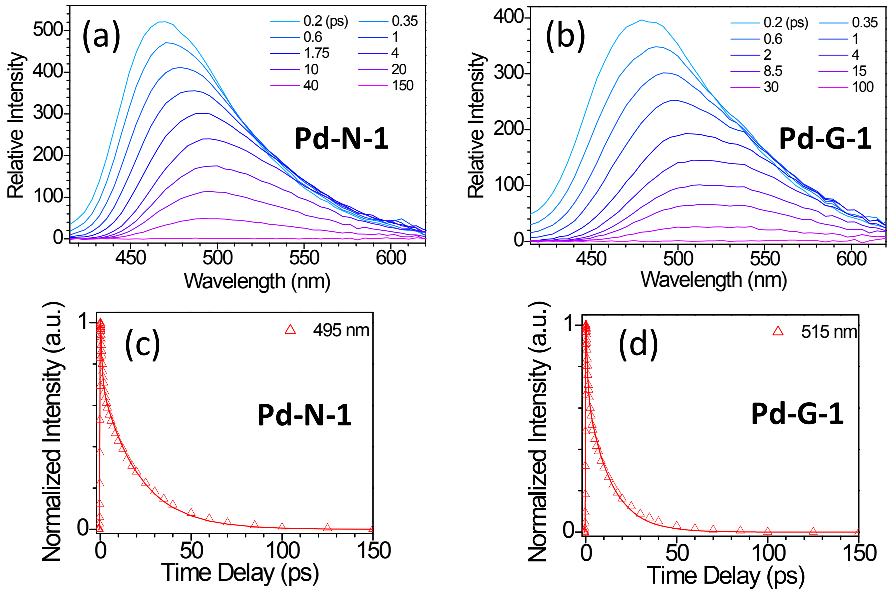

Fig. S18 (a, b) Temporal evolution of fs-TRF and (c, d) experimental (Δ) and fitted (line) kinetic decays of fs-TRF obtained for (a,c) **Pd-N-1** and (b,d) **Pd-G-1** in CH_2Cl_2 with excitation at 300 nm wavelength.

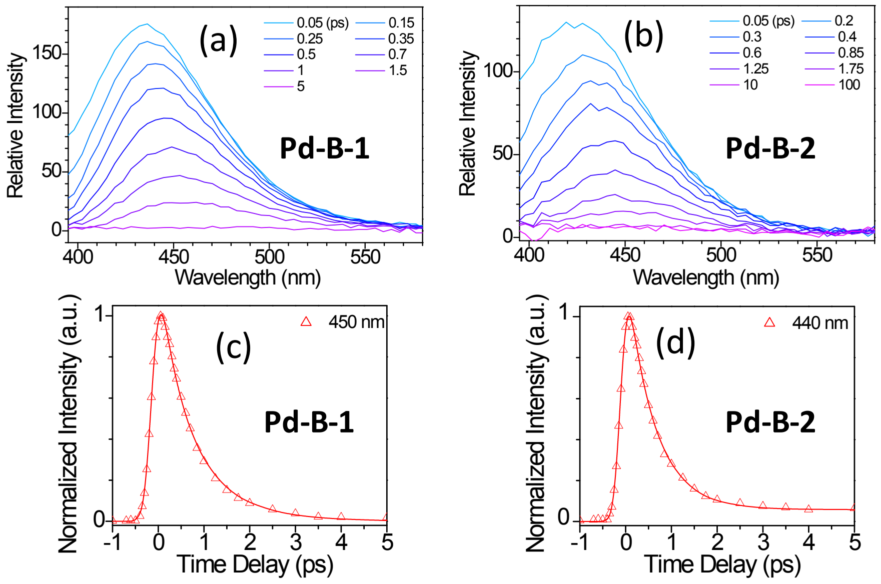

Fig. S19(a, b) Temporal evolution of fs-TRF and (c, d) experimental (Δ) and fitted (line) kinetic decays of fs-TRF obtained for (a,c) **Pd-B-1** and (b,d) **Pd-B-2** in CH_2Cl_2 with excitation at 300 nm wavelength.

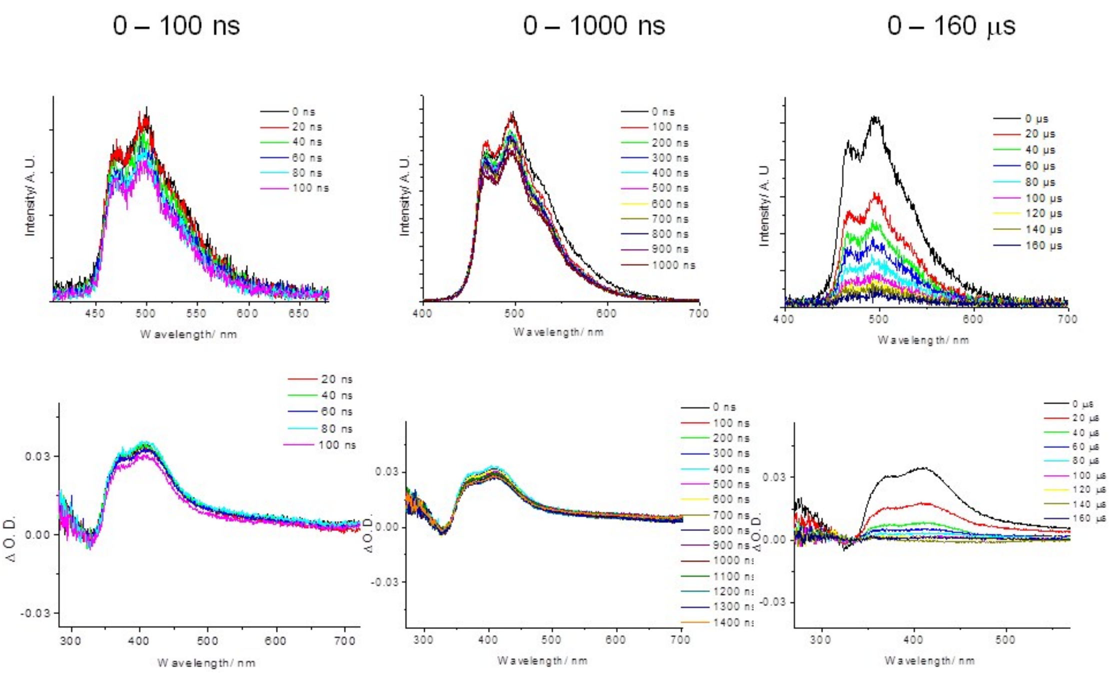

Fig. S20 ns-TRE (upper panel) and ns-TA (lower panel) spectra of **Pd-B-1** in degassed CH_2Cl_2 (5×10^{-5} mol dm^{-3}) at room temperature.

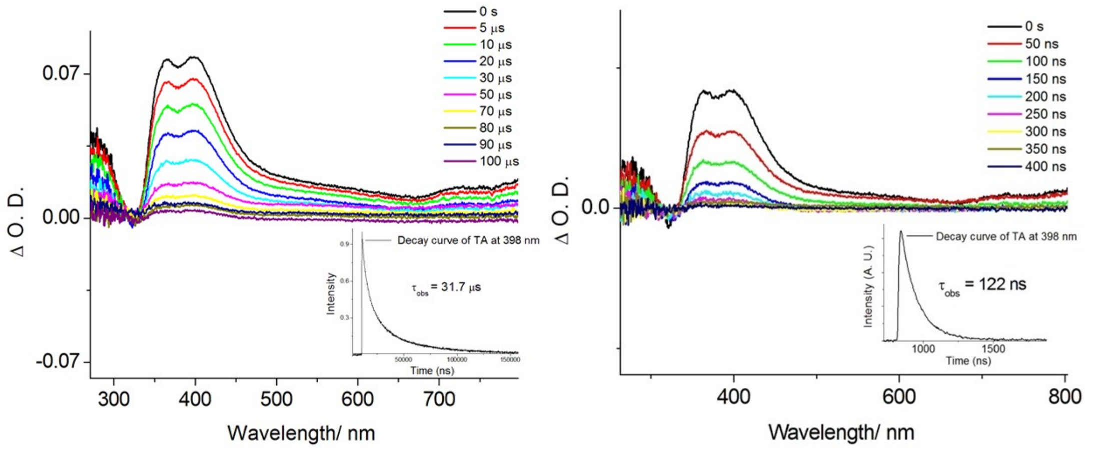

Fig. S21 Left: Time resolved absorption difference spectra of **Pd-B-1** (5×10^{-5} mol dm^{-3}) in degassed CH_3CN monitored at 0–100 μs; Right: Time resolved absorption difference spectra of **Pd-B-1** (5×10^{-5} mol dm^{-3}) in non-degassed CH_3CN monitored at 0–400 ns. Insets in these two graphs show the decays of TA at 398 nm.

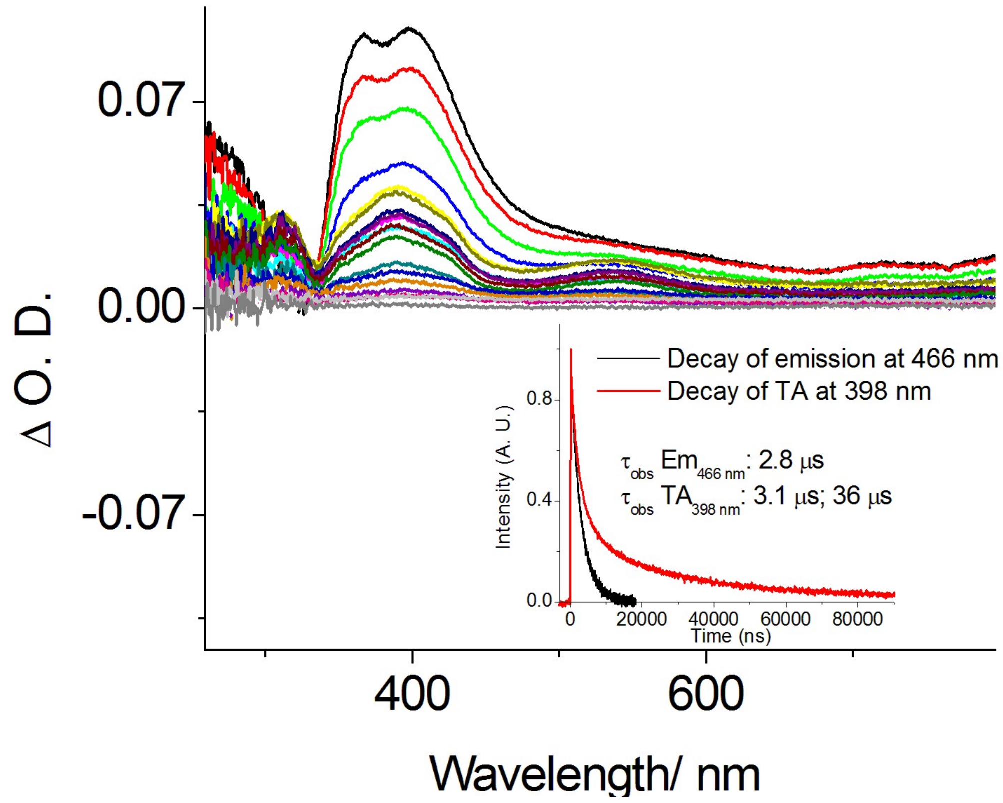

Fig. S22 Time resolved absorption difference spectra of **Pd-B-1** (5×10^{-5} mol dm^{-3}) and TMEDA (0.69 mol dm^{-3}) in degassed CH_3CN monitored at 0–100 μs. Inset shows the decay of emission at 466 nm (black) and TA at 398 nm (red).

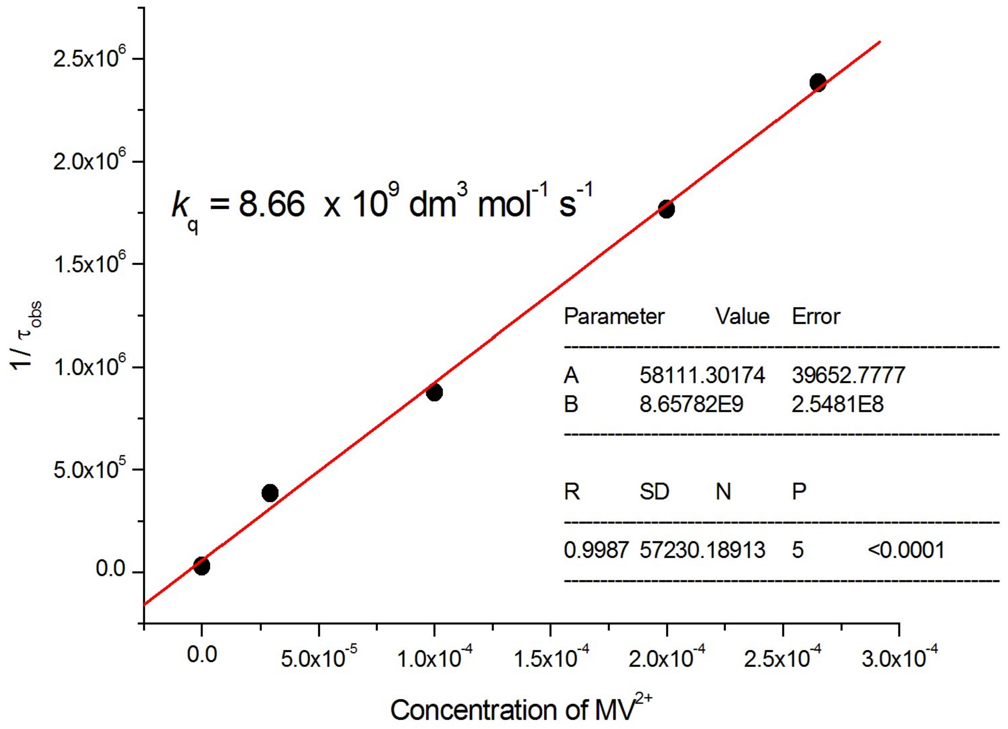

Fig. S23 Stern-Volmer plot of the quenching of **Pd-B-1** at 466 nm by MV^{2+}.

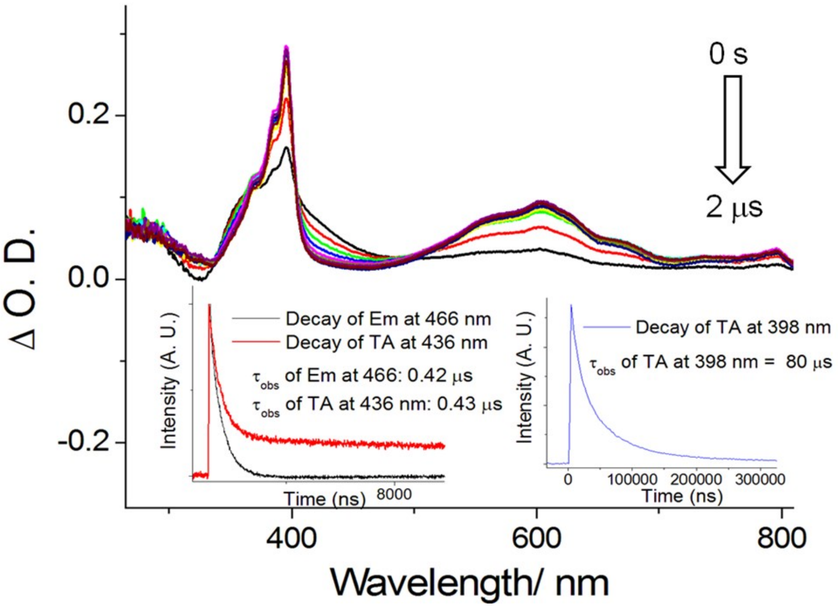

Fig. S24 Time resolved absorption difference spectra of **Pd-B-1** (5×10^{-5} mol dm^{-3}) and $MV(PF_6)_2$ (2.5×10^{-4} mol dm^{-3}) in CH_3CN monitored at 0–2 μs. Insets show the decays of emission at 466 nm (black), TA at 436 nm (red) and TA at 398 (blue).

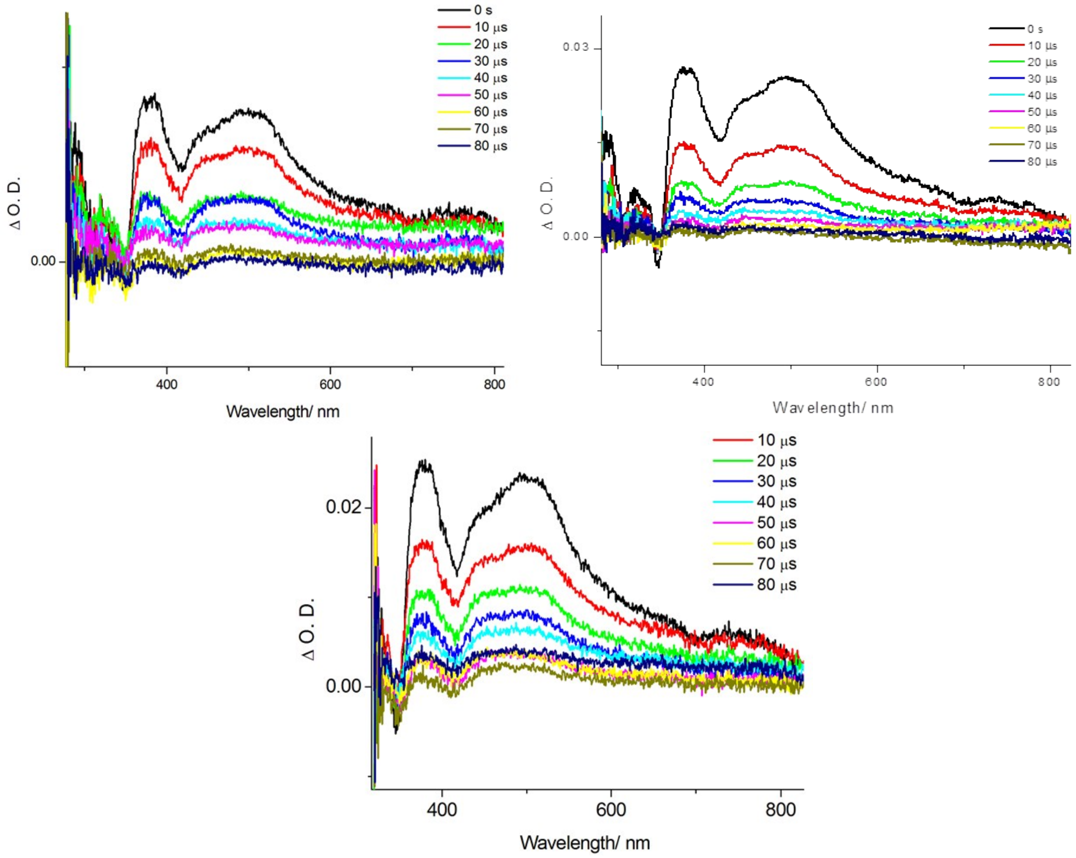

Fig. S25 Top left: time-resolved absorption difference spectra of **Pd-N-1** (5×10^{-5} mol dm^{-3}) in degassed CH_3CN monitored at 0–80 μs; Top right: time-resolved

absorption difference spectra of **Pd-N-1** (5×10^{-5} mol dm^{-3}) and ^{i}PrNEt (1.15 mol dm^{-3}) in degassed CH_3CN monitored at 0–80 μs; Bottom: time-resolved absorption difference spectra of **Pd-N-1** (5×10^{-5} mol dm^{-3}) and substrate $\mathbf{A_1}$ (5×10^{-2} mol dm^{-3}) in degassed CH_3CN monitored at 0–80 μs.

Table S5 Reason for the absence of charge transfer reactions between substrate $\mathbf{E_1}$ and **Pd-B-1**/**Pd-N-1** in excited state

	E_{pa} (V)	$E_{1/2}^{red}$ (V)	E_{0-0} (eV)	E(M*/M^{-1}) (V)	E(M^{+}/M*) (V)
**Pd-N-1**	0.53	–2.47	2.48	0.01	–1.95
**Pd-B-1**	0.81	–2.54	2.71	0.17	–1.90
**Pd-B-2**	0.73	–2.44	2.70	0.26	–1.97
**Pd-G-1**	0.53	–2.24	2.61	0.37	–2.08

Substrate $\mathbf{E_1}$ oxidizes at the potential +1.11 V and reduces at the potential of –2.95 V.

Imaginary cases:

Case 1: $\mathbf{E_1}$ were oxidized by **Pd-N-1** in excited state. Driving force = – 1.1 V;

Case 2: $\mathbf{E_1}$ were reduced by **Pd-N-1** in excited state. Driving force = – 1 V;

Case 3: $\mathbf{E_1}$ were oxidized by **Pd-B-1** in excited state. Driving force = –0.94 V;

Case 4: $\mathbf{E_1}$ were reduced by **Pd-B-1** in excited state. Driving force = – 1.05 V;

Therefore the charge transfer reactions between $\mathbf{E_1}$ and **Pd-N-1**/**Pd-B-1** are highly thermodynamically unfavourable.

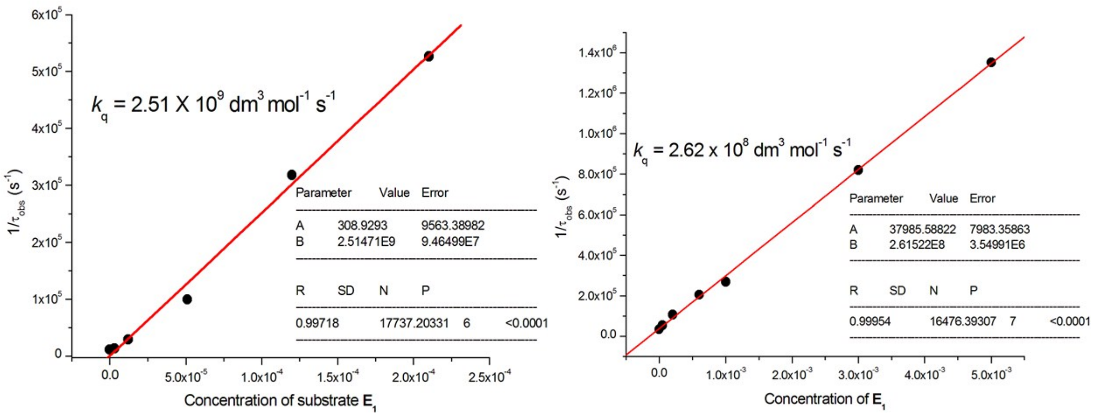

Fig. S26 Left: Stern-Volmer plot of quenching of **Pd-B-1** at 466 nm by substrate $\mathbf{E_1}$; Right: Stern-Volmer plot of quenching of **Pd-N-1** at 515 nm by substrate $\mathbf{E_1}$.

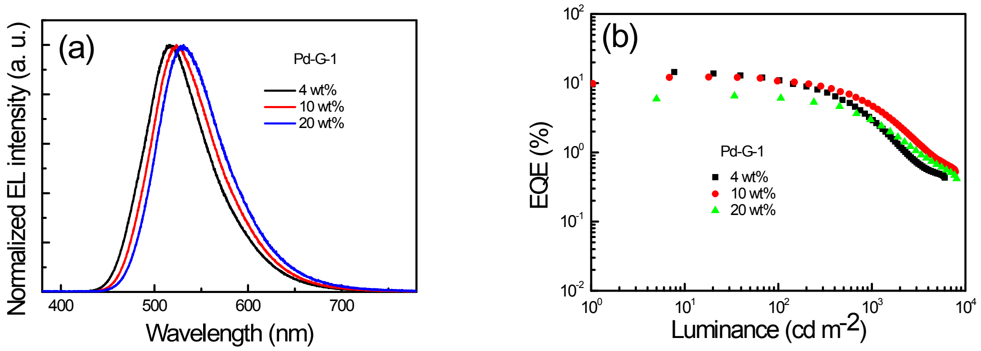

Fig. S27. (a) Normalized EL spectra and (b) external quantum efficiency-luminance characteristics of OLEDs with **Pd-G-1** at the dopant concentrations of 4, 10 and 20 wt%.

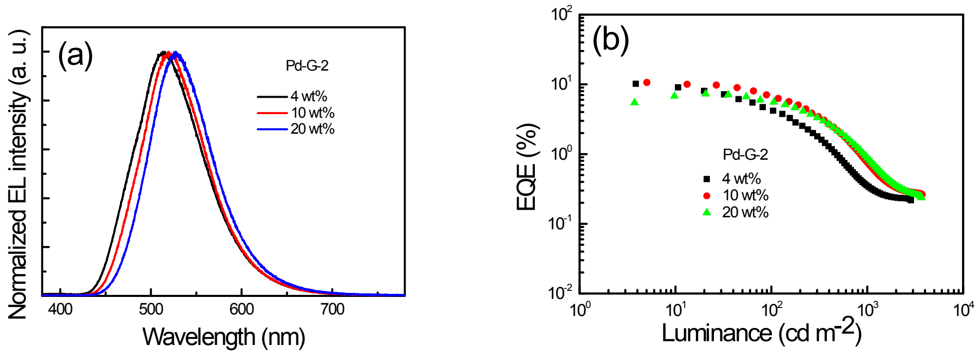

Fig. S28. (a) Normalized EL spectra and (b) external quantum efficiency-luminance characteristics of OLEDs with **Pd-G-2** at the dopant concentrations of 4, 10 and 20 wt%.

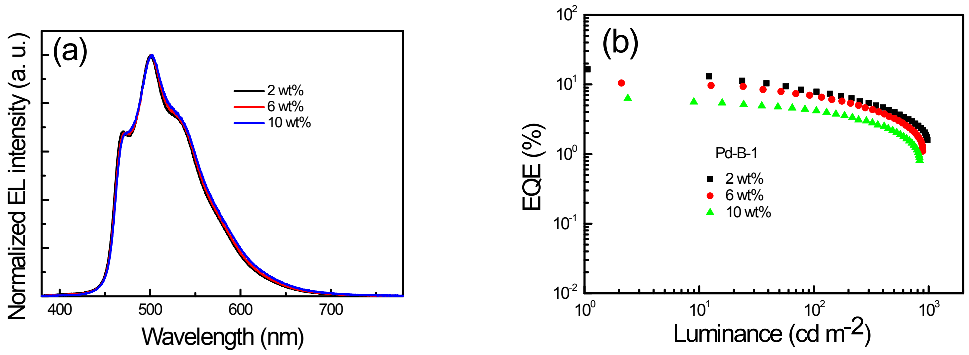

Fig. S29. (a) Normalized EL spectra and (b) external quantum efficiency-luminance characteristics of OLEDs with **Pd-B-1** at the dopant concentrations of 2, 6 and 10 wt%.

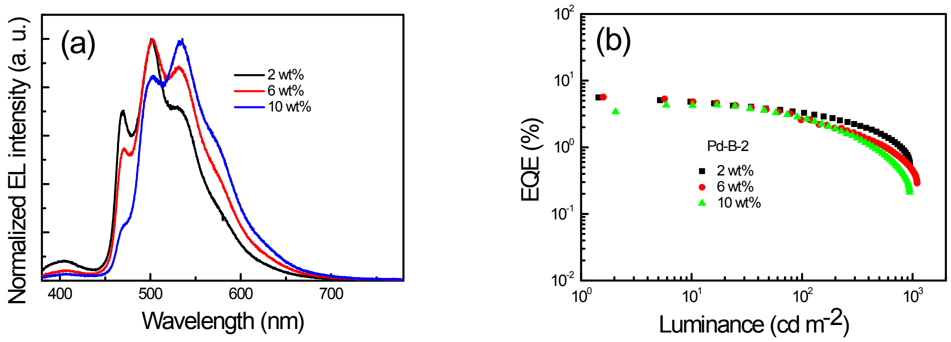

Fig. S30. (a) Normalized EL spectra and (b) external quantum efficiency-luminance characteristics of OLEDs with **Pd-B-2** at the dopant concentrations of 2, 6 and 10 wt%.

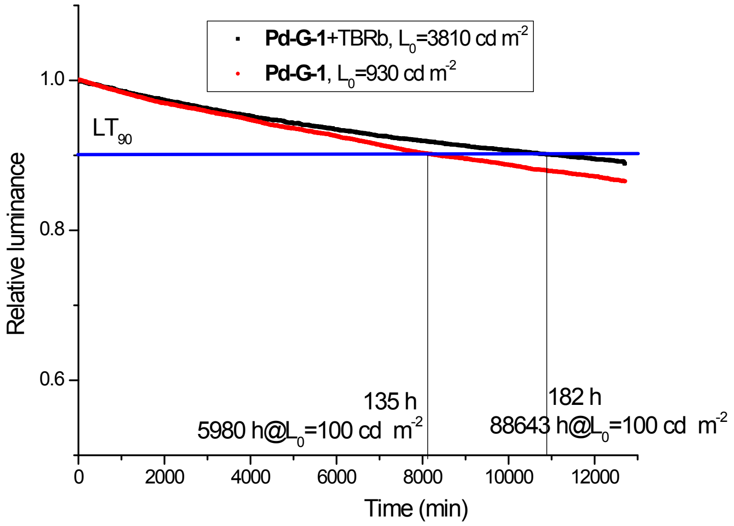

Fig. S31 Device lifetime (T_{90}) of the OLED with **Pd-G-1** as emitter as well as PSF-OLED with **Pd-G-1** as phophorescent sensitizer and TBRb as fluorescent emitter.

Synthesis of ligand precursors, ligands and metal complexes

The syntheses of the ligand of **Pd-G-1**[11], and the complex **Pd-G-2** and its ligand[10] were reported in our previous publications. The ligand of **Pd-B-2** was prepared according to the literature.[12]

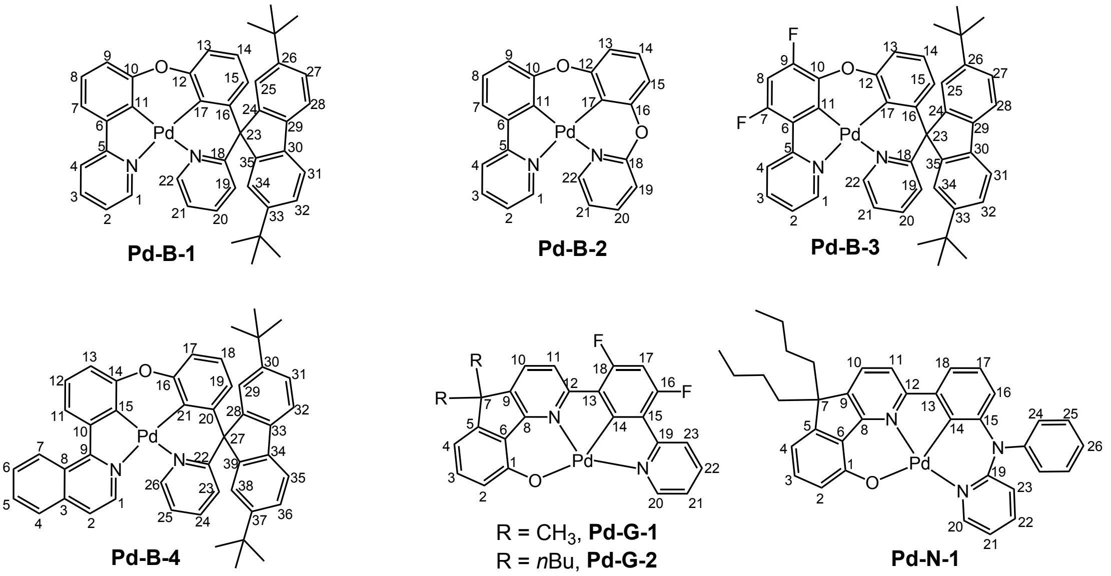

Atomic numbering of the seven complexes

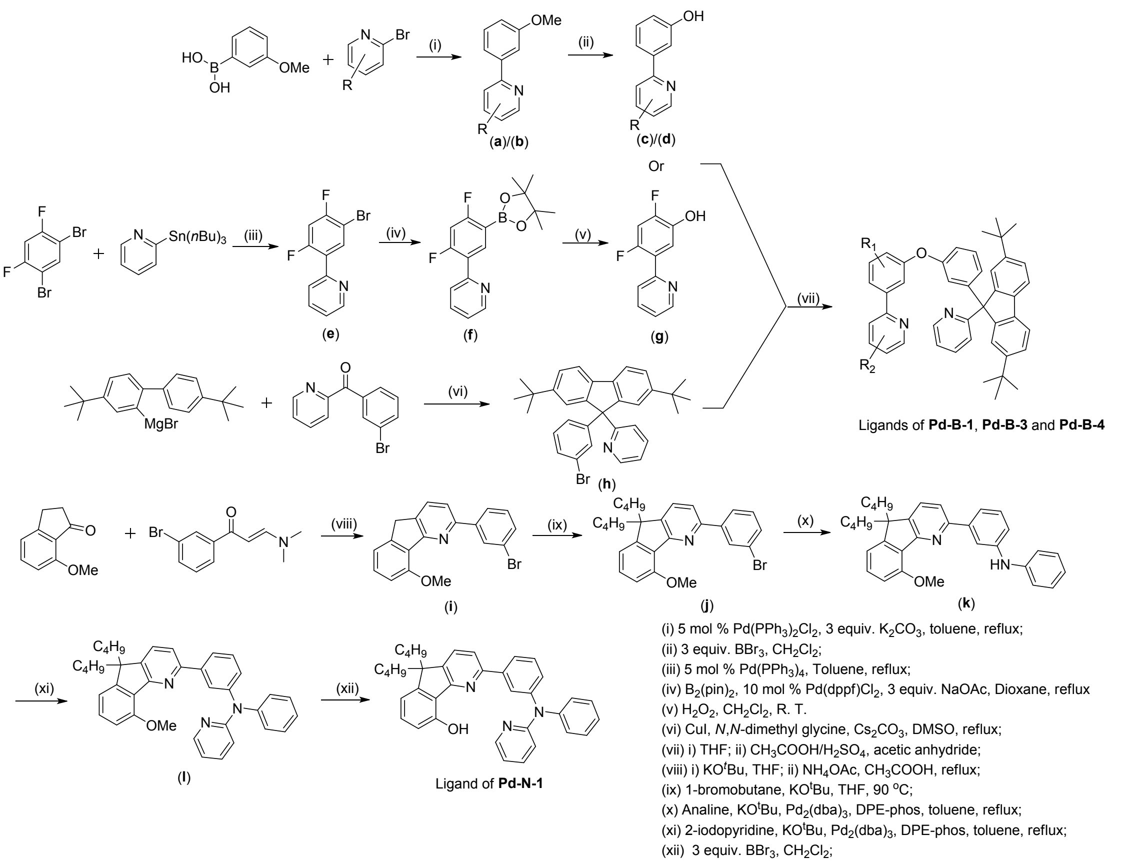

Part 1. Synthesis of ligands precursors

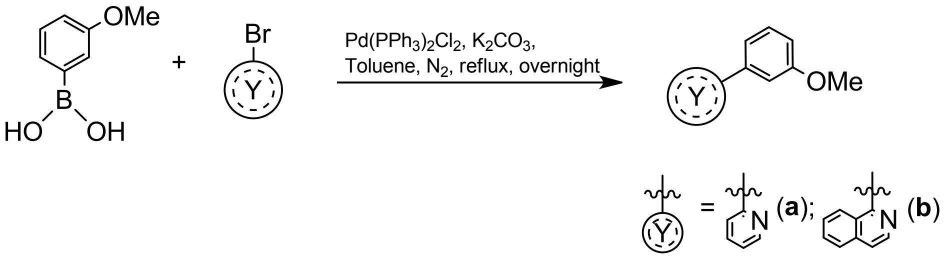

A flask was charged with $Pd(PPh_3)_2Cl_2$ (5 mol%), K_2CO_3 (3.0 equiv.), 3-methoxyphenylboronic acid (1.0 equiv.), 2-bromopyridine/1-bromoisoquinoline (1 equiv.) and 100 mL of anhydrous toluene under nitrogen atmosphere. The reaction mixture was heated to reflux for 20 hours. After cooling down to ambient temperature, the mixture was diluted with dichloromethane and washed with 30 mL water three times. The combined organic extracts were dried over anhydrous $MgSO_4$, concentrated under reduced pressure and the resulting residue was purified by flash chromatogrphy on silica gel to provide the desired product.

(**a**):[15] Prepared from 3-methoxyphenylboronic acid (5.0 g, 32.9 mmol) and 2-bromopyridine (3.1 mL, 32.9 mmol); Yellow oil; Isolated yield: 5.2 g (85%); ^{1}H NMR (400 MHz, $CDCl_3$, 25 ^{o}C): δ = 8.68 (d, J = 4.5 Hz, 1H), 7.76–7.70 (m, 2H),

7.58 (s, 1H), 7.54 (d, J = 7.7 Hz, 1H), 7.38 (t, J = 8.0 Hz, 1H), 7.26–7.21 (m, 1H), 6.96 (d, J = 8.1 Hz, 1H), 3.89 (s, 3H).

(**b**):[16] Prepared from 3-methoxyphenylboronic acid (4.7 g, 30.1 mmol) and 1-bromoisoquinoline (6.3 g, 30.1 mmol); Yellow oil; Isolated yield: 6.5 g (91%); ^{1}H NMR (300 MHz, $CDCl_3$, 25 °C): δ = 8.59 (d, J = 5.7 Hz, 1H), 8.11 (d, J = 8.5 Hz, 1H), 7.83 (d, J = 8.2 Hz, 1H), 7.67–7.60 (m, 2H), 7.52–7.39 (m, 2H), 7.27–7.24 (m, 2H), 7.04 (d, J = 8.4 Hz, 1H), 3.85 (s, 3H).

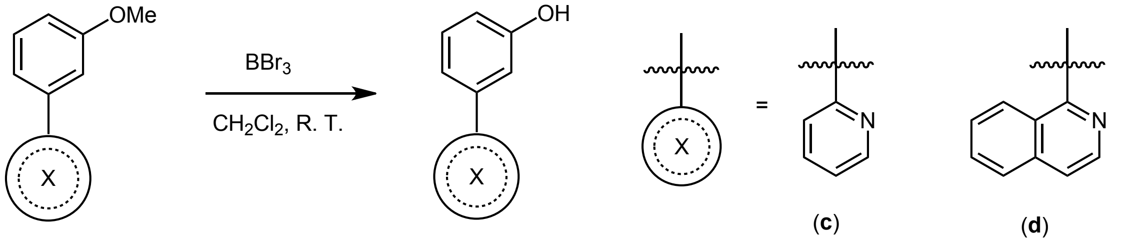

To a solution of aryl methyl ether (1.0 equiv.) in 30 mL of dry dichloromethane, BBr_3 (1.0 mol dm^{-3} in dichloromethane, 3 equiv.) was slowly added under ice bath. The mixture was allowed to stir at room temperature for 2 hours. The crude mixture was slowly poured into crushed ice and neutralized with saturated Na_2CO_3 solution. The organic layer was washed with water twice and extracted with dichloromethane. The solvent was removed under reduced pressure. The crude was purified by flash column chromatography on silica gel using *n*-hexane/ethyl acetate (7: 3 v/v) as eluent to provide the product. Recrystallization from *n*-hexane/diethyl ether mixture gave (**c**)/(**d**) as white solids.

(**c**): Prepared from (**a**) (1.5 g, 8.1 mmol); White solid; Isolated yield: 1.0 g (73%); ^{1}H NMR (400 MHz, $CDCl_3$, 25 °C): δ = 8.68 (d, J = 4.2 Hz, 1H), 7.77 (t, J = 7.2 Hz, 1H), 7.70 (d, J = 7.9 Hz, 1H), 7.57 (s, 1H), 7.46 (d, J = 7.7 Hz, 1H), 7.34 (t, J = 7.9 Hz, 1H), 7.25 (s, 1H), 6.90 (d, J = 8.0 Hz, 1H), 6.03 (s, –OH).

(**d**): Prepared from (**b**) (4.0 g, 17.0 mmol); White solid; Isolated yield: 3.2 g (84%); ^{1}H NMR (400 MHz, CD_3OD, 25 °C): δ = 8.45 (d, J = 5.9 Hz, 1H), 8.09 (d, J = 8.5 Hz, 1H), 8.03 (d, J = 8.2 Hz, 1H), 7.88 (d, J = 8.9 Hz, 1H), 7.83 (m, 1H), 7.65 (t, J = 4.5 Hz, 1H), 7.39 (t, J = 4.1 Hz, 1H), 7.09–6.99 (m, 3H).

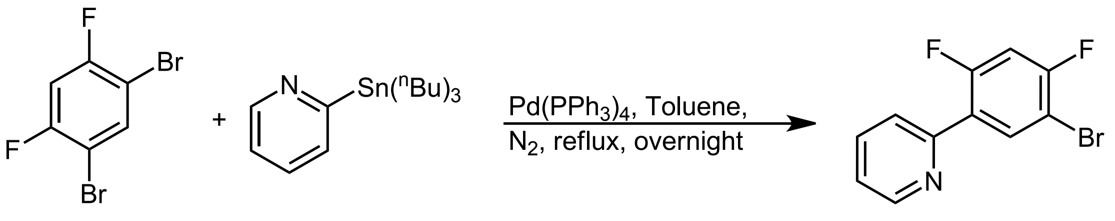

(**e**)

A flask was charged with $Pd(PPh_3)_2Cl_2$ (1.3 g, 1.8 mmol, 5 mol%), 1,5-dibromo-2,4-difluorobenzene (10 g, 36.8 mmol, 1.0 equiv.) and 2-(tributylstannyl)pyridine (11.9 mL, 36.8 mmol, 1.0 equiv.). The flask was evacuated and backfilled with nitrogen. 80 mL of anhydrous toluene was added under nitrogen atmosphere and the mixture was heated to reflux overnight. The crude mixture was filtered through a small portion of celite and then concentrated under reduced pressure. The crude product was purified by flash chromatography on silica gel using *n*-hexane/ethyl acetate (15:1 v/v) as eluent to give the product as a yellow solid.

(**e**) Isolated yield: 7.4 g (75%); ^{1}H NMR (400 MHz, $CDCl_3$, 25 °C): δ = 8.63 (d, J = 4.8 Hz, 1H), 8.21 (t, J = 8.0 Hz, 1H), 7.68 (s, 2H), 7.25–7.19 (m, 1H), 6.91 (t, J = 9.4 Hz, 1H); ^{19}F NMR (376 MHz, $CDCl_3$, 25 °C): δ = –102.62, –113.78.

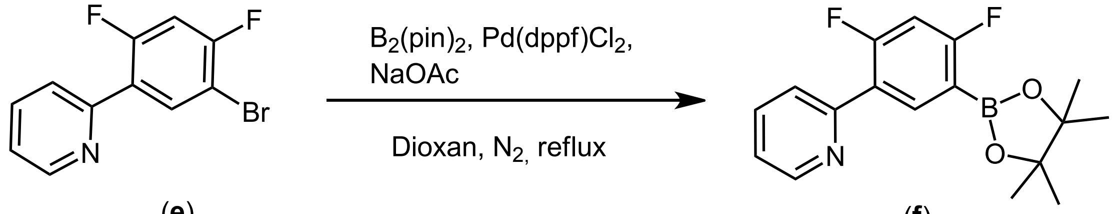

(**e**) (**f**)

A flask was charged with $Pd(dppf)Cl_2$ (2.0 g, 2.5 mmol, 10 mol%), NaOAc (7.3 g, 73.6 mmol, 3.0 equiv.), bis(pinacolato)diboron (9.5 g, 36.8 mmol, 1.5 equiv.) and compound (**e**) (6.6 g, 24.5 mmol, 1.0 equiv.). The flask was evacuated and backfilled with nitrogen. 100 mL of anhydrous 1,4-dioxane was added under nitrogen atmosphere and the mixture was heated to reflux overnight. The crude mixture was filtered through a small portion of celite and then concentrated under reduced pressure. The crude product was purified by flash chromatography on a silica gel column using *n*-hexane/ethyl acetate (10:1 v/v) as eluent to give the product as a pale yellow solid.

(**f**): Isolated yield: 4.7 g (60%); ^{1}H NMR (400 MHz, $CDCl_3$, 25 °C): δ = 8.72 (d, J = 4.6 Hz, 1H), 8.34 (t, J = 8.2 Hz, 1H), 7.77–7.69 (m, 2H), 7.30–7.24 (m, 1H), 6.88 (t, J = 10.1 Hz, 1H), 1.60 (s, 12H); ^{19}F NMR (376 MHz, $CDCl_3$, 25 °C): δ = –98.41, –108.81.

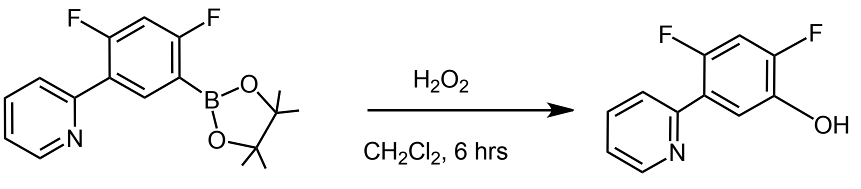

(**f**) (**g**)

A flask was charged with compound (**f**) (2.0 g, 6.3 mmol, 1.0 equiv.); 40 mL of dichloromethane was added to dissolve the solid. 20 mL of 30% (*w*/*w*) (10 equiv.) H_2O_2 was added and the mixture was allowed to stir at room temperature overnight. The crude product was diluted with dichloromethane and washed with 50 mL water three times. The combined organic extracts were dried over anhydrous $MgSO_4$, concentrated under reduced pressure. The resulting residue was purified by flash chromatography on a silica gel column using *n*-hexane/ethyl acetate (10 : 1 v/v) as eluent to give (**g**) as a pale brown solid.

(**g**): Isolated yield: 0.8 g (65%); ^{1}H NMR (400 MHz, $CDCl_3$, 25 ^oC): δ = 8.69 (d, J = 4.7 Hz, 1H), 8.48 (s, –OH), 7.80 (t, J = 7.7 Hz, 1H), 7.72 (d, J = 8.1 Hz, 1H), 7.54 (t, J = 8.6 Hz, 1H), 7.31 (t, J = 6.2 Hz, 1H), 6.89 (t, J = 10.3 Hz, 1H); ^{19}F NMR (376 MHz, $CDCl_3$, 25 ^oC): δ = –124.41, –133.16.

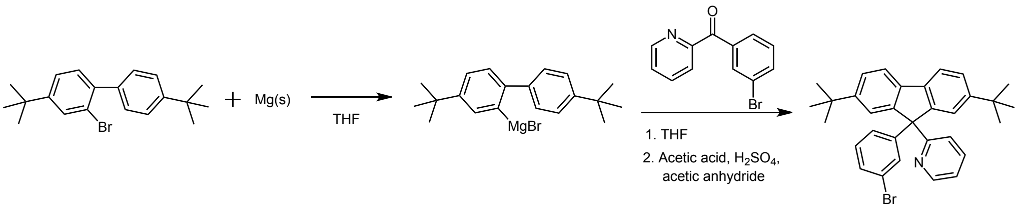

(**h**)

To a Schlenk flask equipped with a magnetic stirrer were added magnesium (0.6 g, 22.8 mmol, 1.2 equiv.) and 4,4'-di-*tert*-2-bromobiphenyl (7.9 g, 22.8 mmol, 1.2 equiv.). The flask was evacuated and subsequently filled with nitrogen for 3 times. 60 mL of anhydrous THF was added to the flask under nitrogen atmosphere. The reaction mixture was heated to reflux until complete consumption of magnesium (indicated by a clear solution) and was then cooled down to ambient temperature. A solution of 2-(3-bromobenzoyl)pyridine (5.0 g, 19.1 mmol, 1.0 equiv.) in 30 mL anhydrous THF was slowly added to the reaction mixture at room temperature under nitrogen atmosphere. The reaction mixture was heated to reflux overnight. After removing the solvent under reduced pressure, the crude product was poured into a solution containing 5.0 mL concentrated H_2SO_4, 5.0 mL acetic anhydride and 90 mL glacial acetic acid. The reaction mixture was stirred at 150 ^oC for 6 hours. The resultant mixture was poured into cool CH_3OH. After filtration and washing with cool methanol twice, the crude product was obtained as a pale yellow solid. Further

purification was done by flash chromatography on a silica gel column using hexane/ethyl acetate (15:1 v/v) as eluent to provide the desired product (**h**) as a pale yellow solid.

(**h**): Pale yellow solid; Yield: 7.9 g (81%). ^{1}H NMR (300 MHz, $CDCl_3$, 25 °C): δ = 7.60 (d, J = 1.7 Hz, 2H), 7.56 (d, J = 7.9 Hz, 2H), 7.32 (dd, J = 8.0 Hz, J = 1.8 Hz, 2H), 7.23–7.19 (m, 3H), 7.13–7.09 (m, 2H), 6.97 (dd, J = 5.8 Hz, J = 2.7 Hz, 1H), 6.90–6.87 (m, 2H), 1.26 (s, 18H).

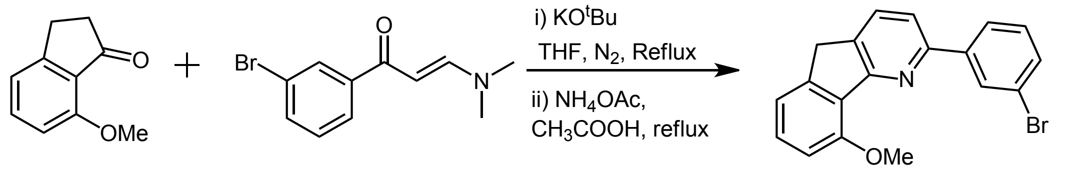

(i)

To a dry, nitrogen-flushed flask was charged with 7-methoxy-2,3-dihydro-1*H*-inden-1-one (2.5 g, 15 mmol, 1.0 equiv.), 1-(3-bromophenyl)-3-(dimethylamino)prop-2-en-1-one (3.92 g, 15 mmol, 1.0 equiv.), potassium *tert*-butoxide (2.08 g, 18 mmol, 1.2 equiv.) and anhydrous THF. The mixture was stirred for 12 hours. Excess ammonium acetate (~25 g) and acetic acid (~50 mL) was added and the resultant mixture was reflux for 2 hours. After cooling to room temperature, the crude was extracted with $CHCl_3$ (3 × 20 mL). The combined organic layer was dried over anhydrous $MgSO_4$. After removal of the volatiles under reduced pressure, the crude was purified by flash column chromatography on a silica gel column using *n*-hexane/ethyl acetate (10:1 v/v), yielding (**i**) as yellow oil.

(**i**): Isolated yield: 3.80 g (70%); ^{1}H NMR (400 MHz, $CDCl_3$, 25 °C): δ = 8.35 (m, 1H), 8.12 (d, J = 8.1 Hz, 1H), 7.85 (d, J = 7.9 Hz, 1H), 7.61 (d, J = 7.9 Hz, 1H), 7.52 (d, J = 8.1 Hz, 1H), 7.35–7.40 (m, 2H), 7.20 (d, J = 7.5 Hz, 1H), 6.98 (d, J = 8.3 Hz, 1H), 4.12 (s, 3H, OCH_3), 3.92 (s, 2H, $-CH_2-$).

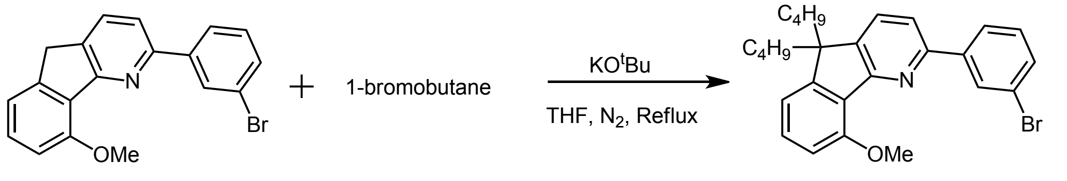

(i) (j)

To a dry, nitrogen-flushed flack was charged with (**i**) (2.0 g, 5.68 mmol, 1.0 equiv.), 1-bromobutane (1.5 mL, 14 mmol, 2.5 equiv.) and anhydrous THF. The mixture was refluxed for 12 hours. The crude was extracted with $CHCl_3$ (3 × 20 mL). The

combined organic layer was dried over anhydrous $MgSO_4$ (s). After removal of the volatiles under reduced pressure, the crude was purified by flash column chromatography on a silica gel column using *n*-hexane/ethyl acetate (10:1 v/v) as eluent. Compound (**j**) was obtained as a yellow solid.

‘

(**j**): Isolated yield: 2.03 g (77%); ^{1}H NMR (400 MHz, $CDCl_3$, 25 ^oC): δ = 8.38 (s, 1H), 8.10 (d, J = 7.8 Hz, 1H), 7.63 (q, J = 7.9 Hz, 2H), 7.51 (d, J = 8.0 Hz, 1H), 7.32–7.42 (m, 2H), 7.00 (d, J = 7.6 Hz, 1H), 6.93 (d, J = 8.0 Hz, 1H), 4.11 (s, 3H, $-OCH_3$), 1.96–2.01 (s, 4H, $-\underline{CH_2}CH_2CH_2CH_3$), 1.02–1.10 (m, 4H, $-CH_2\underline{CH_2}CH_2CH_3$), 0.57–0.67 (m, 10H, $-CH_2CH_2\underline{CH_2CH_3}$).

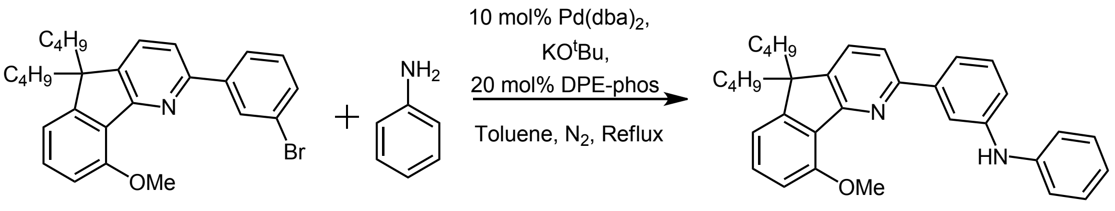

(**j**) (**k**)

To a dry, nitrogen-flushed flask was charged with (**j**) (1.5 g, 3.23 mmol, 1.0 equiv.), potassium *tert*-butoxide (0.43 g, 3.88 mmol, 1.2 equiv.), $Pd(dba)_2$ (0.19 g, 0.32 mmol, 10 mol%), DPE-phos (0.35 g, 0.65 mmol, 20 mol%), aniline (0.29 mL, 3.23 mmol, 1.0 equiv.), and anhydrous toluene, The mixture was reflux for 24 hours. After cooling to room temperature, ethyl acetate was added, and the mixture was stirred for five minutes. The crude was extracted with $CHCl_3$ (3 × 20 mL). The combined organic layer was dried over anhydrous $MgSO_4$. After removal of the volatiles under reduced pressure, the crude was purified by flash column chromatography on a silica gel column with *n*-hexane/ethyl acetate (10:1 v/v) as eluent. Compound (**k**) was obtained as a yellow solid.

(**k**): Isolated yield: 1.14 g (74%); ^{1}H NMR (300 MHz, $CDCl_3$, 25 ^oC): δ = 8.00 (s, 1H), 7.69 (d, J = 7.8 Hz, 1H), 7.62 (s, 2H), 7.33–7.41 (m, 3H), 7.30 (s, 1H), 7.13–7.15 (m, 3H), 6.90–7.00 (m, 3H), 5.82 (s, 1H), 4.05 (s, 3H, $-OCH_3$), 1.95–2.00 (m, 4H, $-\underline{CH_2}CH_2CH_2CH_3$), 1.02–1.10 (m, 4H, $-CH_2\underline{CH_2}CH_2CH_3$), 0.57–0.67 (m, 10H, $-CH_2CH_2\underline{CH_2CH_3}$).

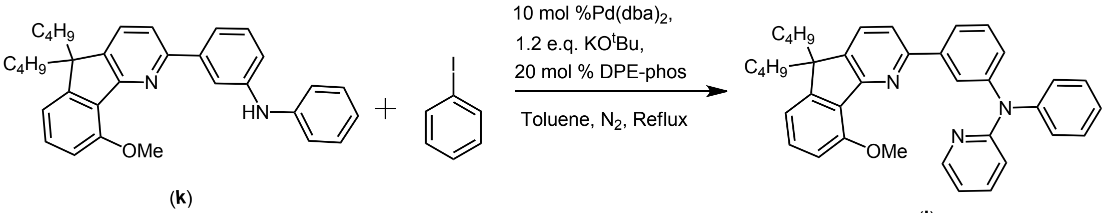

(**k**)

(**l**)

To a dry, nitrogen-flushed flask was charged with (**k**) (1.00 g, 2.10 mmol, 1.0 equiv.), potassium *tert*-butoxide (0.28 g, 2.52 mmol, 1.2 equiv.), Pd(dba)$_2$ (0.12 g, 0.21 mmol, 10 mol%), DPE-phos (0.23 g, 0.42 mmol, 20 mol%), 2-iodopyridine (0.19 mL, 2.10 mmol, 1.0 equiv.), and anhydrous toluene. The mixture was refluxed for 24 hours. After cooling to room temperature, ethyl acetate was added, and the mixture was stirred for five minutes. The crude was extracted with $CHCl_3$ (3 × 20 mL). The combined organic layer was dried over anhydrous $MgSO_4$. After removal of the volatiles under reduced pressure, the crude was purified by flash column chromatography on a silica gel column using *n*-hexane/ethyl acetate (10:1 v/v) as eluent. Compound (**l**) was yielded as a yellow solid.

(**l**): Isolated yield: 0.81 g (70%); ^{1}H NMR (400 MHz, $CDCl_3$, 25 ^oC): δ = 8.26 (d, J = 7.7 Hz, 1H), 8.05 (s, 1H), 7.93 (d, J = 7.9 Hz, 1H), 7.60 (d, J = 7.8 Hz, 1H), 7.55 (d, J = 8.0 Hz, 1H), 7.45 (q, J = 8.3 Hz, 2H), 7.30–7.38 (m, 4H), 7.20–7.23 (m, 2H), 7.11 (t, J = 7.0 Hz, 1H), 6.97 (d, J = 7.5 Hz, 1H), 6.85 (d, J = 8.0 Hz, 1H), 6.81 (d, J = 7.7 Hz, 1H), 6.78 (t, J = 6.5 Hz, 1H), 3.95 (s, 3H, –OCH_3), 1.55–1.97 (m, 4H, –$\underline{CH_2}CH_2CH_2CH_3$), 1.00–1.07 (m, 4H, –$CH_2\underline{CH_2}CH_2CH_3$), 0.53–0.65 (m, 10H, –$CH_2CH_2\underline{CH_2CH_3}$).

Part 2. Synthesis of ligands of Pd-B-1, Pd-B-3, Pd-B-4 and Pd-N-1

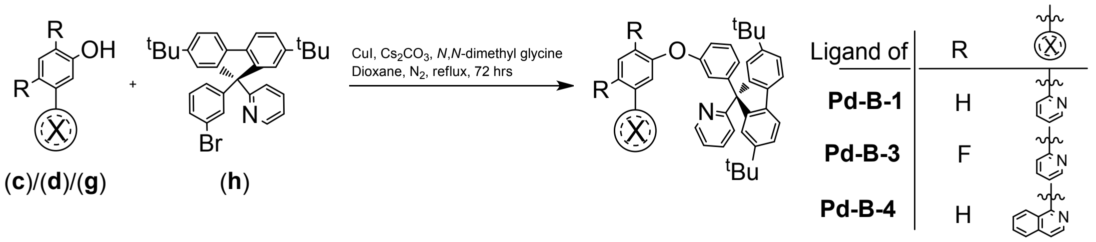

To a Schlenk flask equipped with a magnetic stirrer was were added CuI (10 mol%), Cs_2CO_3 (3.0 equiv.), *N*,*N*-dimethyl glycine (30 mol%), (**c**)/(**d**)/(**g**) (1.0 equiv.) and (**h**) (1.0 equiv.). The flask was evacuated and subsequently filled with nitrogen for 3 times. 100 mL of anhydrous DMSO was added to the flask under nitrogen atmosphere. The mixture was stirred at 150 ^oC for 3 days. The solid was filtered off and the filtrate was washed with deionized water for three times, extracted with dichloromethane and then dried over anhydrous $MgSO_4$. The mixture was concentrated and purified by flash chromatography on a silica gel column using *n*-hexane/ethyl acetate mixture as eluent to give desired product as off-white solids.

Ligand of **Pd-B-1**: Prepared from (**c**) (0.5 g, 2.9 mmol) and (**h**) (1.5 g, 2.9 mmol); Off-white solid; Isolated yield: 0.71 g (41%); ^{1}H NMR (500 MHz, $CDCl_3$, 25 ^oC): δ =

8.66 (d, J = 4.5 Hz, 2H, H^1, H^{23}), 7.70 (dt, J = 2.2 Hz, J = 7.1 Hz, 1H, H^3), 7.70 (d, J = 8.2 Hz, 1H, H^9), 7.67–7.58 (m, 6H, H^{28}, H^{25}, H^{17}, H^4), 7.42–7.32 (m, 4H, H^{27}, H^{21}, H^8), 7.22 (dt, J = 2.1 Hz, J = 6.5 Hz, 1H, H^2), 7.20 (t, J = 8.1 Hz, 1H, H^{14}), 7.15 (dt, J = 3.2 Hz, J = 7.5 Hz, 1H, H^{22}), 6.96–6.92 (m, 2H, H^{20}, H^7), 6.84–6.79 (m, 3H, H^{15}, H^{13}, H^{11}), 1.27 (s, 18H, ^{t}Bu); EI-MS(+ve, m/z): 600 [M^+].

Ligand of **Pd-B-3**: Prepared from (**g**) (1.0 g, 4.8 mmol) and (**h**) (2.5 g, 4.8 mmol); Off-white solid; Isolated yield: 1.9 g (61%); ^{1}H NMR (600 MHz, $CDCl_3$, 25 °C): δ = 8.64 (d, J = 4.4 Hz, 1H, H^1), 8.64 (d, J = 6.2 Hz, 1H, H^{23}), 7.77–7.69 (m, 3H, H^8, H^4, H^3), 7.61 (d, J = 8.0 Hz, 2H, H^{28}), 7.58 (s, 2H, H^{25}), 7.40 (t, J = 7.5 Hz, 1H, H^{21}), 7.37 (d, J = 8.0 Hz, 2H, H^{27}), 7.22 (t, J = 5.6 Hz, 1H, H^2), 7.14 (t, J = 7.9 Hz, 1H, H^{14}), 7.07 (t, J = 5.2 Hz, 1H, H^{22}), 6.96–6.92(m, 2H, H^{20}, H^{17}), 6.79–6.74 (m, 3H, H^{15}, H^{13}, H^{11}), 1.26 (s, 18H, ^{t}Bu); ^{19}F NMR (376 MHz, $CDCl_3$, 25 °C): δ = –115.36, –125.14; EI-MS(+ve, m/z): 636 [M^+].

Ligand of **Pd-B-4**: Prepared from (**d**) (0.5 g, 2.3 mmol) and (**h**) (1.2 g, 2.3 mmol); Off-white solid; Isolated yield: 0.66 g (44%); ^{1}H NMR (600 MHz, $CDCl_3$, 25 °C): δ = 8.61 (d, J = 4.8 Hz, 1H, H^{27}), 8.57 (d, J = 6.7 Hz, 1H, H^1), 8.06 (d, J = 8.5 Hz, 1H, H^7), 7.87 (d, J = 8.2 Hz, 1H, H^4), 7.68 (t, J = 7.1 Hz, 1H, H^5), 7.65–7.59 (m, 5H, H^{32}, H^{29}, H^2), 7.52 (t, J = 7.2 Hz, 1H, H^6), 7.41–7.32 (m, 6H, H^{31}, H^{25}, H^{15}, H^{12}, H^{11}), 7.17 (t, J = 7.9 Hz, 1H, H^{18}), 7.06–7.02 (m, 2H, H^{26}, H^{13}), 6.93–6.86 (m, 3H, H^{24}, H^{21}, H^{17}), 6.77 (d, J = 7.1 Hz, 1H, H^{19}), 1.26 (s, 18H, ^{t}Bu); EI-MS(+ve, m/z): 650 [M^+].

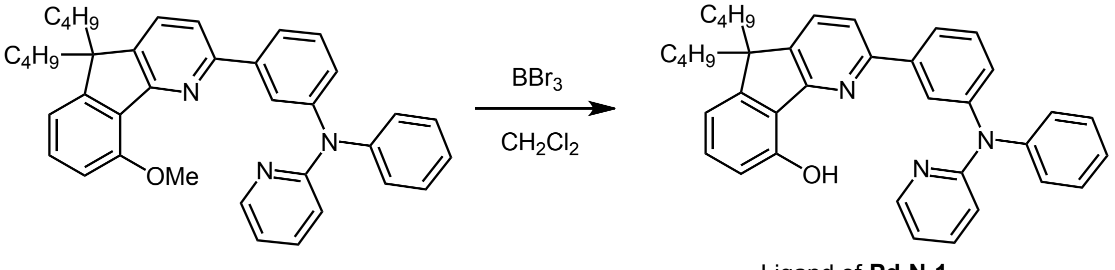

(**l**)

Ligand of **Pd-N-1**

To a solution of (**l**) (0.75 g, 1.35 mmol, 1.0 equiv.) in CH_2Cl_2 chilled in an ice bath was added BBr_3 (4.1 mL 1 mol dm^{-3} BBr_3 solution in CH_2Cl_2, 4.06 mmol, 3.0 equiv.) dropwise. The reaction mixture was warmed to room temperature and stirred for 3 hours. A solution of Na_2CO_3 (1 mol dm^{-3} in H_2O) was added dropwise until all the solids dissolved. The resultant mixture was extracted with $CHCl_3$ (3 × 20 mL). The combined organic layer was dried over anhydrous $MgSO_4$. After removal of all the volatiles under reduced pressure, the crude was purified on a silica gel column using n-hexane/ethyl acetate (8:2 v/v) as eluent. The ligand of **Pd-N-1** was yielded as a pale

yellow solid.

Ligand of **Pd-N-1**: Isolated yield: 0.66 g (90%); ^{1}H NMR (500 MHz, CD_2Cl_2, 25 ^{o}C): δ = 9.17 (s, 1H), 8.21 (d, J = 5.2 Hz, 1H), 7.83 (s, 1H), 7.79 (d, J = 8.0 Hz, 1H), 7.68 (d, J = 7.9 Hz, 1H), 7.54 (d, J = 7.9 Hz, 1H), 7.50 (t, J = 7.8 Hz, 1H), 7.45 (t, J = 7.9 Hz, 1H), 7.36 (t, J = 7.9 Hz, 2H), 7.31 (t, J = 7.9 Hz, 1H), 7.21–7.25 (m, 3H), 7.18 (t, J = 7.4 Hz, 1H), 6.93 (d, J = 7.3 Hz, 1H), 6.82–6.84 (m, 3H), 1.92–1.97 (m, 4H, –$\underline{CH_2}CH_2CH_2CH_3$), 1.06–1.10 (m, 4H, –$CH_2\underline{CH_2}CH_2CH_3$), 0.66–0.71 (m, 10H, –$CH_2CH_2\underline{CH_2CH_3}$); EI-MS (+ve, m/z): 539 [M^{+}].

Part 3. Synthesis of metal complexes

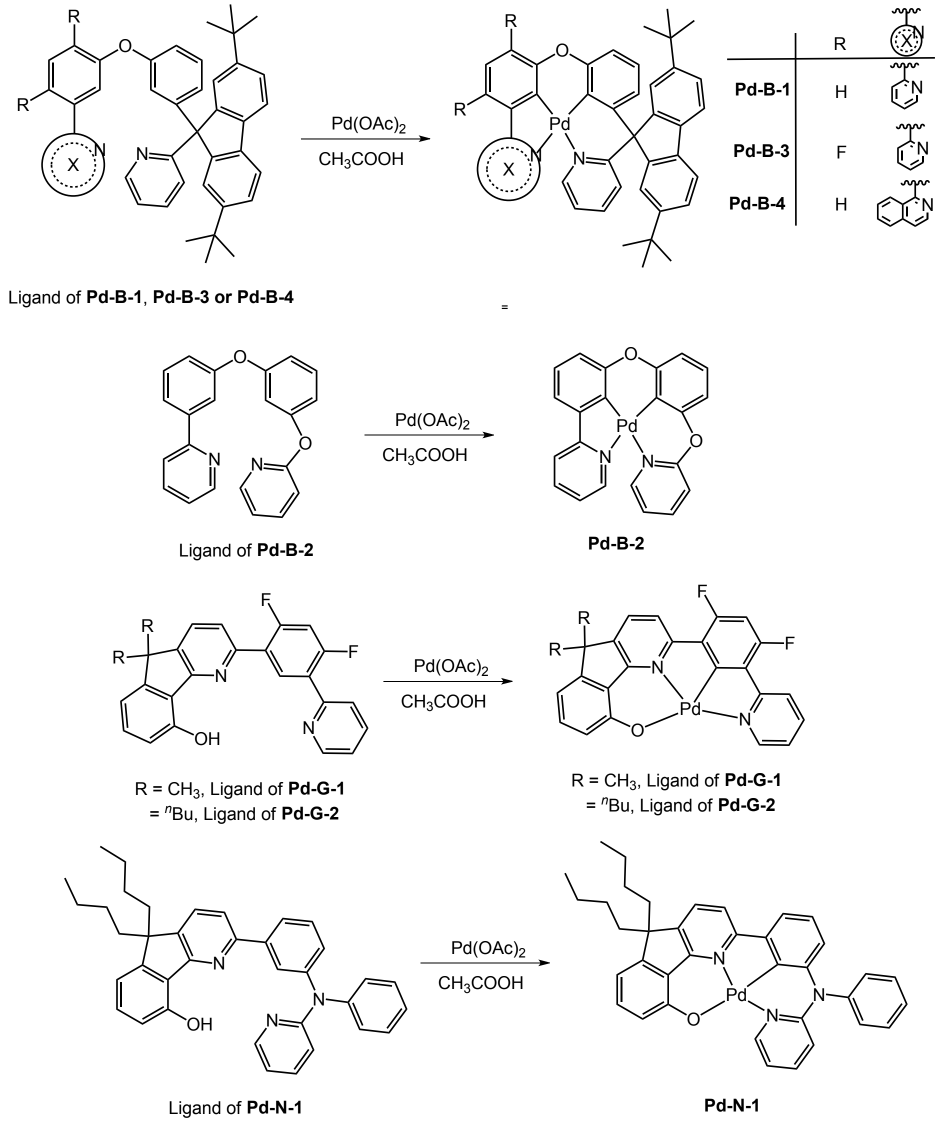

As shown in the above scheme, the ligand was mixed with 1.1 equiv. of $Pd(OAc)_2$ in glacial acetic acid (50 mL), and the reaction mixture was refluxed for 12 hours. After that, 50 mL dionized water was poured into the reaction mixture and the resultant

mixture was extracted with $CHCl_3$ (3 × 50 mL). The combined organic layer was dried over anhydrous $MgSO_4$. The solvent was removed under reduced pressure. Purification was done by flash column chromatography on an alumina column using CH_2Cl_2 or $CHCl_3$ as eluent to give the product as yellow solids. **Pd-B-1**, **Pd-B-2**, **Pd-G-1** and **Pd-G-2** were further purified by sublimation at 280–300 °C under 4 × 10^{-5} Torr, and were harvested as pale yellow/yellow crystalline solids.

Pd-B-1: Prepared from the corresponding ligand (0.30 g, 0.50 mmol) and $Pd(OAc)_2$ (0.12 g, 0.55 mmol); Pale yellow crystalline solid; Isolated yield: 0.12 g (33%); 1H NMR (500 MHz, $CDCl_3$, 25 °C): δ = 10.0 (s, 1H, H^{25}), 9.0 (d, J = 5.3 Hz, 1H, H^{22}), 8.11 (d, J = 5.2 Hz, 1H, H^{1}), 8.02 (d, J = 8.1 Hz, 1H, H^{4}), 7.87 (t, J = 7.5 Hz, 1H, H^{3}), 7.77 (s, 1H, H^{34}), 7.52–7.58 (m, 5H, H^{7}, H^{9}, H^{20}, H^{27}, H^{28}), 7.21–7.31 (m, 4H, H^{8}, H^{21}, H^{31}, H^{32}), 7.13–7.18 (m, 2H, H^{2}, H^{15}), 6.97 (d, J = 8.4 Hz, 1H, H^{19}), 6.84 (t, J = 7.8 Hz, 1H, H^{14}), 6.64 (d, J = 7.8 Hz, 1H, H^{13}), 1.39 (s, 9H, ^{t}Bu), 0.76 (s, 9H, *t*Bu); ^{13}C NMR (126 MHz, $CDCl_3$, 25°C): δ = 165.1, 164.6, 162.9, 153.5, 153.0, 152.0, 150.5, 150.4, 147.9, 147.6, 143.5, 140.4, 137.9, 137.6, 131.2, 126.8, 126.7, 125.4, 125.2, 125.1, 124.8, 124.5, 123.9, 122.8, 122.1, 121.3, 119.7, 119.6, 118.9, 118.2, 116.9, 116.1, 71.1, 31.6, 31.0; FAB-MS(+ve, *m/z*): 704 [M^+]; Elemental analyses calcd (%) for $C_{43}H_{38}N_2OPd$: C 73.24, H 5.43, N 3.97. Found: C 73.23, H 5.38, N 3.99.

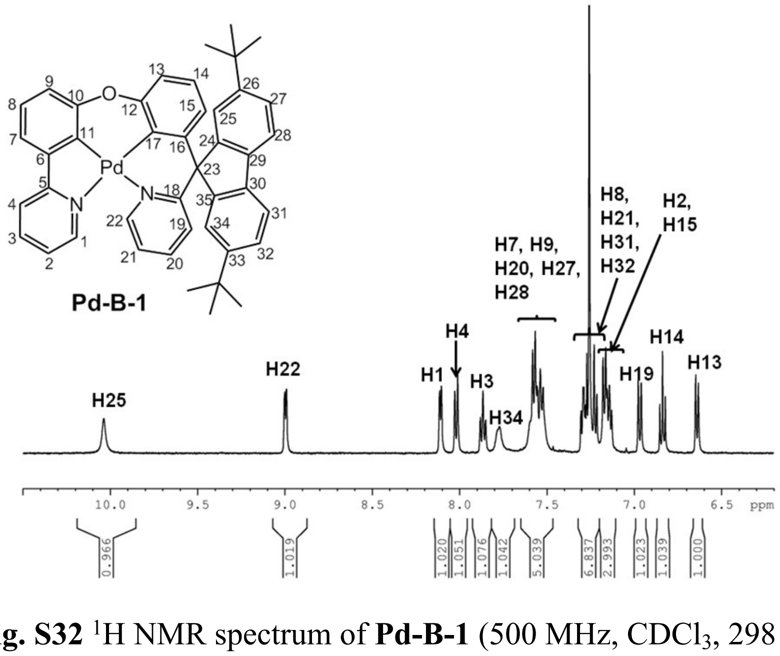

Fig. S32 ^{1}H NMR spectrum of **Pd-B-1** (500 MHz, $CDCl_3$, 298 K).

Pd-B-2: Prepared from the corresponding ligand (0.26 g, 0.76 mmol) and $Pd(OAc)_2$ (0.19 g, 0.84 mmol); Pale yellow crystalline solid; Isolated yield: 0.13 g (37%); ^{1}H NMR (500 MHz, $CDCl_3$, 25 °C): δ = 8.50–8.51 (m, 1H, H^{22}), 8.34 (d, J = 4.7 Hz, 1H, H^{1}), 7.95 (d, J = 8.1 Hz, 1H, H^{4}), 7.91 (ddd, J = 1.9 Hz, 7.2 Hz, 8.4 Hz, 1H, H^{21}), 7.86

(ddd, J = 1.6 Hz, 7.5 Hz, 8.1 Hz, 1H, H^2), 7.52 (dd, J = 1.0 Hz, 7.2 Hz, 1H, H^7), 7.36 (d, J = 8.3 Hz, 1H, H^{19}), 7.21–7.28 (m, 4H, H^3, H^8, H^9, H^{20}), 7.13–7.19 (m, 2H, H^{14}, H^{15}), 6.95 (dd, J = 1.5 Hz, 7.3 Hz, 1H, H^{13}); ^{13}C NMR (126 MHz, $CDCl_3$, 25 °C): δ = 164.7, 160.7, 156.4, 153.4, 152.2, 147.9, 147.7, 147.7, 140.6, 138.1, 136.6, 125.7, 125.6, 122.3, 119.7, 119.6, 118.4, 117.7, 116.1, 115.3, 113.0, 110.8; FAB-MS(+ve, *m*/*z*): 444 [M^+]; Elemental analyses calcd (%) for $C_{22}H_{14}N_2O_2Pd$: C 59.41, H 3.17, N 6.30. Found: C 59.21, H 3.32, N 6.29.

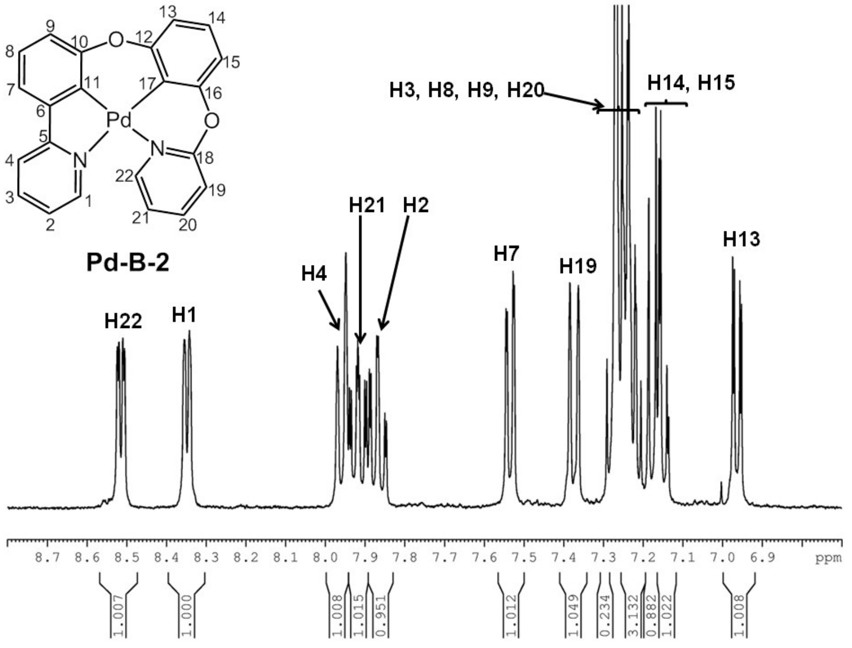

Fig. S33 ^{1}H NMR spectrum of **Pd-B-2** (500 MHz, $CDCl_3$, 298 K).

Pd-B-3: Prepared from the corresponding ligand (0.22 g, 0.35 mmol) and $Pd(OAc)_2$ (0.085 g, 0.38 mmol); Pale yellow solid; Isolated yield: 0.10 g (40%); ^{1}H NMR (500 MHz, $CDCl_3$, 25 °C): δ = 9.84 (s, 1H, H^{25}), 8.98 (dd, J = 1.35 Hz, 5.5 Hz, 1H, H^{22}), 8.30 (d, J = 8.3 Hz, 1H, H^4), 8.04 (dd, J = 1.0 Hz, 5.4 Hz, 1H, H^1), 7.87 (t, J = 7.6 Hz, 1H, H^3), 7.77 (d, J = 7.9 Hz, 1H, H^{28}), 7.59 (d, J = 7.7 Hz, 1H, H^{27}), 7.50–7.54 (m, 3H, H^{20}, H^{32}, H^{34}), 7.21–7.25 (m, signals overlap with residue solvent peak, 2H, H^{13}, H^{21}), 7.16 (d, J = 7.6 Hz, 1H, H^{31}), 7.13 (ddd, J = 1.1 Hz, 5.5 Hz, 6.9 Hz, 1H, H^2), 6.93 (d, J = 8.4 Hz, 1H, H^{19}), 6.84 (t, J = 7.8, 1H, H^{14}), 6.80 (t, J = 10.8 Hz, 1H, H^8), 6.62 (dd, J = 1.2 Hz, 7.8 Hz, 1H, H^{15}), 1.38 (s, 9H, ^{t}Bu), 0.73 (s, 9H, ^{t}Bu); ^{13}C NMR (126 MHz, $CDCl_3$, 25 °C): δ = 162.7, 161.9 (d), 156.0 (d), 153.9 (d), 152.6, 152.5, 150.7, 150.6, 150.5, 150.4, 149.7, 147.8, 145.5, 143.1, 139.3, 138.4, 137.8, 136.4, 131.0, 127.9, 126.6, 125.3, 124.9, 124.7, 123.6, 123.5, 123.3, 123.0, 122.1, 121.5, 119.7, 119.0, 116.2, 101.4 (dd), 71.0, 31.6, 30.9; ^{19}F NMR (376 MHz, $CDCl_3$, 25 °C): δ = –118.04, –126.75; FAB-MS(+ve, *m*/*z*): 740 [M^+]; Elemental analyses calcd (%) for $C_{43}H_{36}F_2N_2OPd \cdot H_2O$: C 68.03, H 5.05, N 3.69. Found: C 67.71, H 5.02, N 3.64.

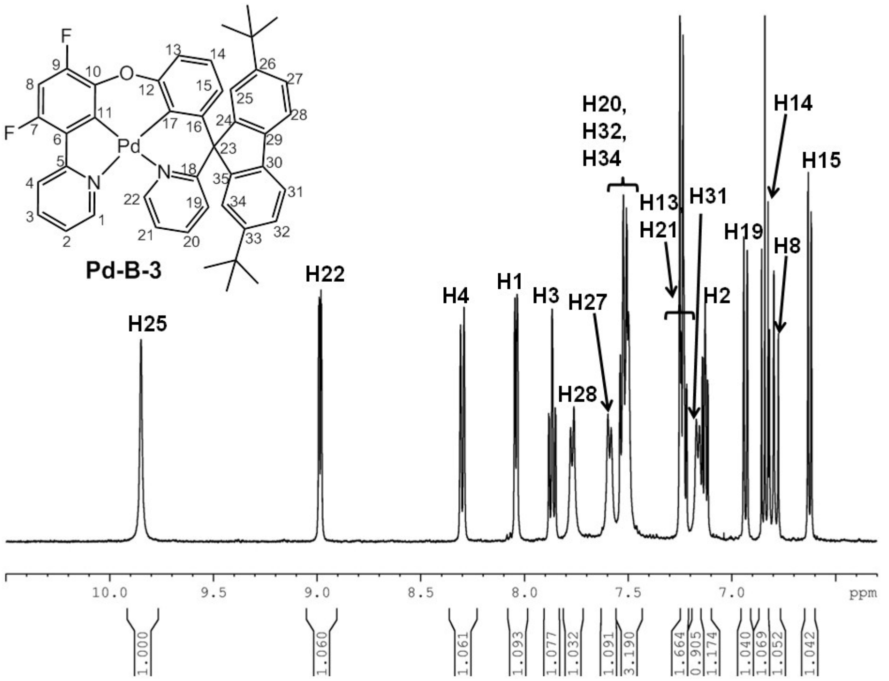

Fig. S34 ^{1}H NMR spectrum of **Pd-B-3** (500 MHz, $CDCl_3$, 298 K).

Pd-B-4: Prepared from the corresponding ligand (0.25 g, 0.38 mmol) and $Pd(OAc)_2$ (0.095 g, 0.42 mmol); Yellow solid; Isolated yield: 0.13 g (46%); ^{1}H NMR (600 MHz, $CDCl_3$, 25 °C): δ = 9.91 (s, 1H, H^{29}), 9.22–9.23 (m, 1H, H^{26}), 8.97 (d, J = 8.5 Hz, 1H, H^{4}), 8.04 (d, J = 6.1 Hz, 1H, H^{1}), 7.91 (d, J = 7.4 Hz, 1H, H^{13}), 7.86 (d, J = 7.9 Hz, 1H, H^{7}), 7.77–7.81 (m, 3H, H^{5}, H^{6}, H^{32}), 7.48–7.75 (m, 5H, H^{2}, H^{24}, H^{31}, H^{35}, H^{36}), 7.35–7.38 (m, 2H, H^{12}, H^{25}), 7.31 (d, J = 7.8 Hz, 1H, H^{11}), 7.18 (dd, J = 1.0 Hz, 7.7 Hz, 1H, H^{17}), 7.08 (s, 1H, H^{38}), 6.97 (d, J = 8.4 Hz, 1H, H^{23}), 6.84 (t, J = 7.8 Hz, 1H, H^{18}), 6.61 (dd, J = 1.0 Hz, 7.7 Hz, 1H, H^{19}), 1.39 (s, 9H, ^{t}Bu), 0.42 (s, 9H, ^{t}Bu); ^{13}C NMR (126 MHz, $CDCl_3$, 25 °C): δ = 167.0, 162.8, 153.0, 150.6, 150.3, 149.9, 148.9, 143.4, 142.2, 139.5, 139.2, 137.7, 137.6, 136.5, 131.2, 130.9, 128.8, 128.2, 127.0, 126.7, 126.3, 125.2, 125.1, 125.0, 124.5, 124.4, 123.9, 122.9, 121.5, 120.1, 119.6, 118.8, 116.6, 115.8, 70.9, 31.6, 30.5; FAB-MS(+ve, *m*/*z*): 754 [M^{+}]; Elemental analyses calcd (%) for $C_{47}H_{40}N_2OPd$•$0.5CHCl_3$•$0.5H_2O$: C 69.24, H 5.08, N 3.40. Found: C 69.39, H 5.34, N 3.32.

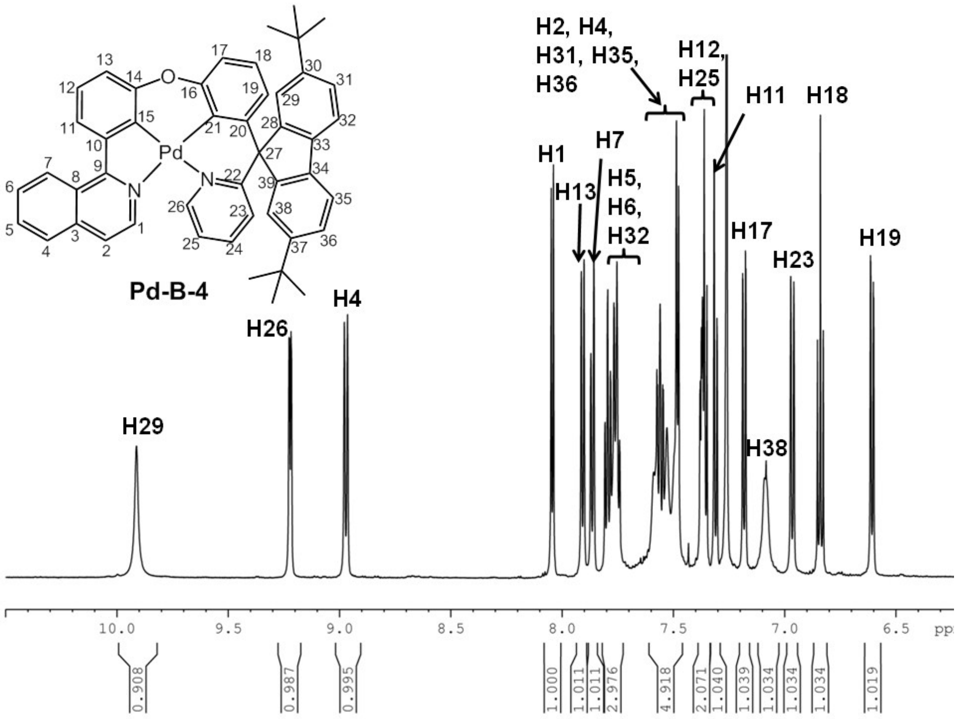

Fig. S35 ^{1}H NMR spectrum of **Pd-B-4** (600 MHz, $CDCl_3$, 298 K).

Pd-G-1: Prepared from the corresponding ligand (0.27 g, 0.67 mmol) and $Pd(OAc)_2$ (0.18 g, 0.74 mmol); Yellow crystalline solid; Isolated yield: 0.19 g (57%); ^{1}H NMR (500 MHz, $CDCl_3$, 25 °C): δ = 8.84–8.85 (m, 1H, H^{20}), 7.79 (d, J = 7.7 Hz, 1H, H^{10}), 7.75–7.56 (m, 2H, H^{21}, H^{22}), 7.52 (d, J = 7.6 Hz, 1H, H^{11}), 7.40 (t, J = 7.5 Hz, 1H, H^3), 7.17–7.20 (m, 1H, H^{21}), 7.03 (d, J = 8.4 Hz, 1H, H^4), 6.70 (d, J = 7.1 Hz, 1H, H^2), 6.54 (t, J = 11 Hz, 1H, H^{17}), 1.87 (s, 6H, $-CH_3$); ^{13}C NMR (126 MHz, $CDCl_3$, 25 °C): δ = 163.7, 162.3, 159.0, 158.9, 157.2, 156.9, 156.8, 156.7, 156.3, 155.4, 150.9, 138.1, 133.2, 129.8, 126.2, 123.0, 122.7, 122.1 (d), 119.7, 116.1 (d), 107.3, 100.2 (t), 46.9, 26.8; ^{19}F NMR (376 MHz, $CDCl_3$, 25 °C): δ = –109.07 (t, J = 11.3 Hz), –109.8 (t, J = 11.3 Hz); FAB-MS(+ve, *m*/*z*): 504 [M^+]; Elemental analyses calcd (%) for $C_{25}H_{16}F_2N_2OPd$: C 59.48, H 3.19, N 5.55. Found: C 59.21, H 3.12, N 5.66.

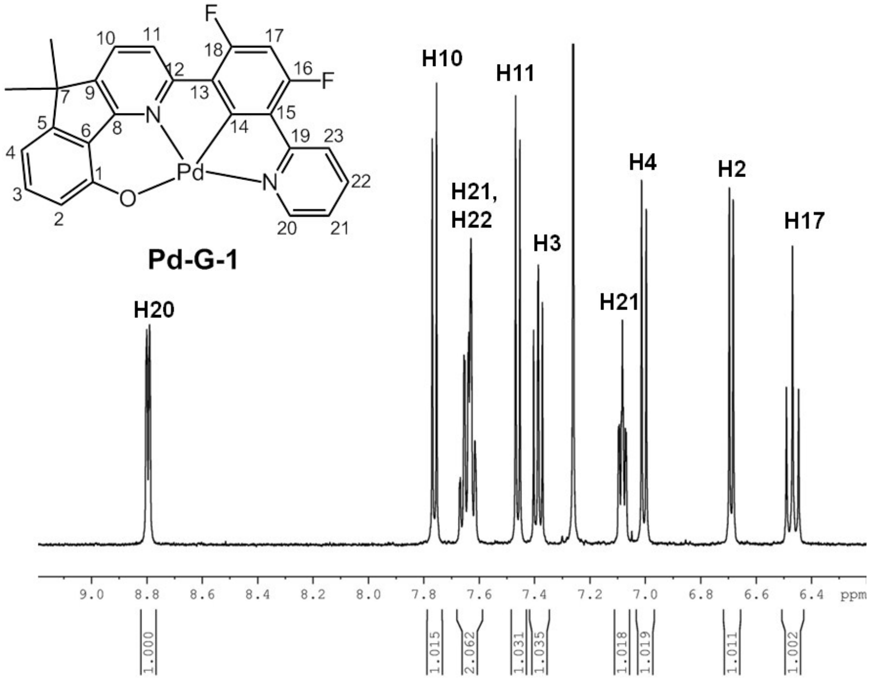

Fig. S36 ^{1}H NMR spectrum of **Pd-G-1** (500 MHz, $CDCl_3$, 298 K).

Pd-N-1: Prepared from the corresponding ligand (0.22 g, 0.41 mmol) and $Pd(OAc)_2$ (0.1 g, 0.45 mmol); Yellow solid; Isolated yield: 0.15 g (55%); ^{1}H NMR (600 MHz, $CDCl_3$, 25 °C): δ= 10.0 (m, 1H, H^{20}), 7.71 (d, J = 7.7 Hz, 1H, H^{10}), 7.68 (t, J = 7.6 Hz, 2H, H^{25}), 7.59 (t, J = 7.7 Hz, 1H, H^{26}), 7.53 (d, J = 7.7 Hz, 1H, H^{11}), 7.42–7.46 (m, 2H, H^{18}, H^{22}), 7.39 (t, J = 7.7 Hz, 1H, H^{3}), 7.34 (d, J = 7.8 Hz, 2H, H^{24}), 7.02 (d, J = 8.2 Hz, 1H, H^{2}), 6.99 (t, J = 7.9 Hz, 1H, H^{17}), 6.90 (t, J = 6.4 Hz, 1H, H^{21}), 6.58 (d, J = 7.0 Hz, 1H, H^{4}), 6.37 (d, J = 8.9 Hz, 1H, H^{23}), 6.15 (d, J = 8.2 Hz, 1H, H^{16}), 1.96–2.04 (m, 4H, –$\underline{CH_2}CH_2CH_2CH_3$), 1.08–1.26 (m, 4H, –$CH_2\underline{CH_2}CH_2CH_3$), 0.67–0.80 (m, 10H, –$CH_2CH_2\underline{CH_2CH_3}$); FAB-MS(+ve, m/z): 643 [M^+]; Elemental analyses calcd (%) for $C_{37}H_{35}N_3OPd$: C 68.99, H 5.48, N 6.52. Found: C 68.77, H 5.55, N 6.30.

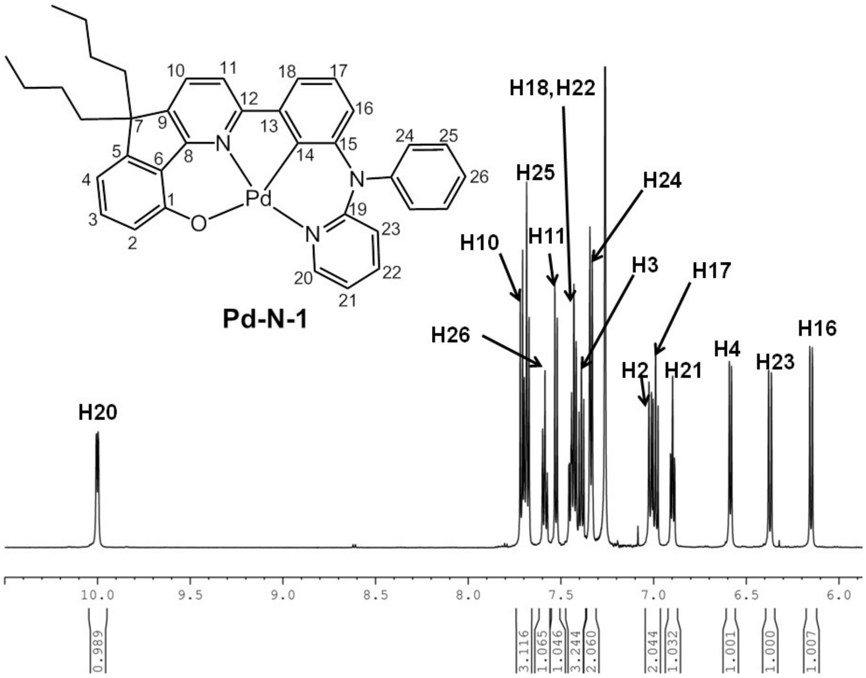

Fig. S37 ^{1}H NMR spectrum of **Pd-N-1** (600 MHz, $CDCl_3$, 298 K).

NMR analysis for the photochemical reactions

All the NMR data of substrates and products of the photochemical reactions in this work were matched with those reported in literatures.[13,14]

Showing below is example of NMR spectra of the resultant mixture of the photochemical reactions:

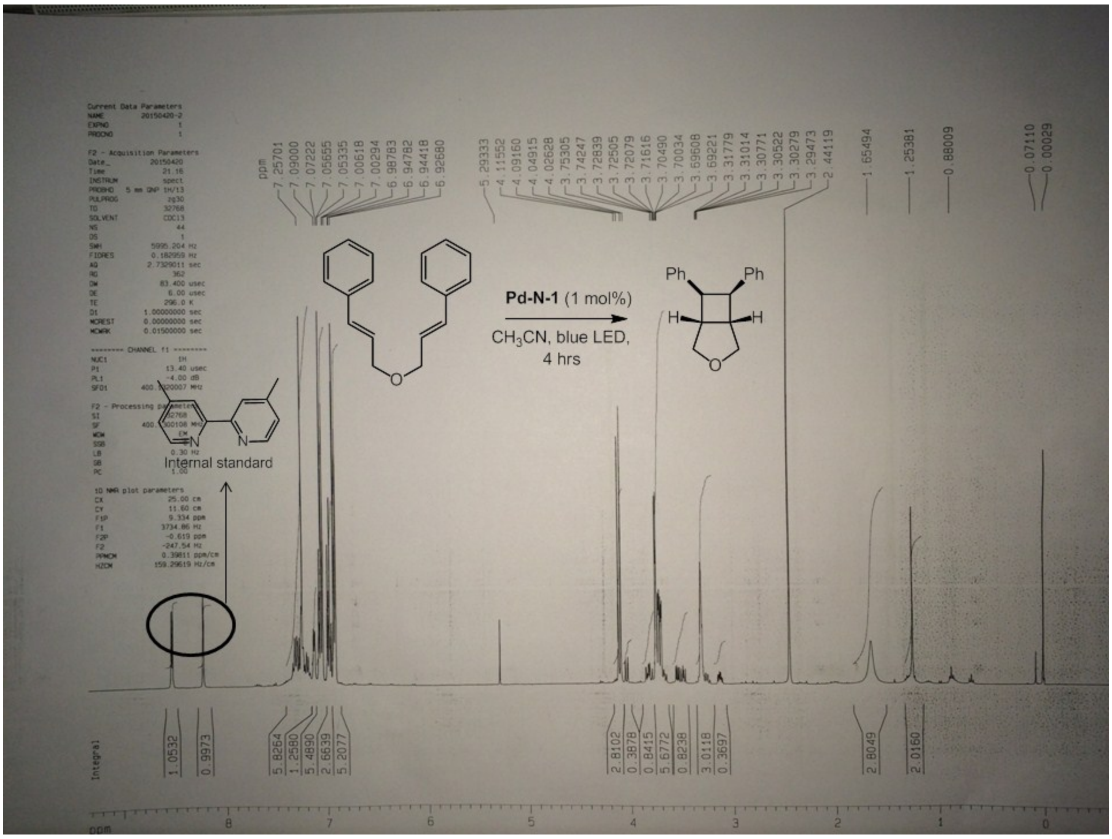

Cartesian coordinates of the optimized geometries

Table S6 S_0 state of **Pd-B-1**

Center Number	Atomic Number	Atomic Type	Coordinates (Angstroms) X	Y	Z
1	46	0	0.006397	0.035220	0.044492
2	7	0	0.013697	0.062713	2.192515
3	6	0	1.920524	-0.195226	0.369454
4	8	0	2.599628	-0.559529	-1.929816
5	6	0	0.307917	0.376138	-1.883279
6	7	0	-2.065069	-0.094077	-0.282290
7	6	0	-2.710164	-0.983289	0.495663
8	1	0	-2.077820	-1.591319	1.135418
9	6	0	2.869448	-0.512241	-0.594155
10	6	0	-2.756672	0.656082	-1.152178
11	6	0	-4.157552	0.593763	-1.157649
12	1	0	-4.720178	1.234132	-1.824168
13	6	0	1.522692	0.066830	-2.518083
14	6	0	-1.016025	0.329841	3.003895
15	1	0	-1.992166	0.391999	2.533085
16	6	0	0.818667	1.017210	-4.603103
17	1	0	1.004610	1.268686	-5.643780
18	6	0	1.267702	-0.002285	2.708871
19	6	0	-0.874677	0.522061	4.370539
20	1	0	-1.744900	0.733334	4.982323
21	6	0	-4.084903	-1.127860	0.502674
22	1	0	-4.552747	-1.863323	1.147931
23	6	0	1.776598	0.352170	-3.861510
24	1	0	2.734917	0.066086	-4.285687
25	6	0	2.324987	-0.253775	1.723093
26	6	0	0.403862	0.440039	4.915648
27	1	0	0.562862	0.587223	5.979850
28	6	0	4.183735	-0.849484	-0.251782
29	1	0	4.890413	-1.088993	-1.041858
30	6	0	1.479424	0.180626	4.078575
31	1	0	2.485475	0.136588	4.480271
32	6	0	-4.825583	-0.291027	-0.325684
33	1	0	-5.910540	-0.335832	-0.337857
34	6	0	-0.660561	1.058332	-2.666140
35	6	0	-0.380898	1.380972	-4.002233
36	1	0	-1.104988	1.930775	-4.590878
37	6	0	4.563768	-0.876459	1.085444
38	1	0	5.584515	-1.137344	1.350377
39	6	0	3.637638	-0.581283	2.081585
40	1	0	3.938687	-0.634121	3.124088
41	6	0	-5.040792	0.815708	-6.243202
42	6	0	-2.570254	3.952477	-2.059010
43	6	0	-1.807507	2.967080	-1.415308
44	6	0	-4.178734	3.882679	-4.106494
45	1	0	-4.445136	4.936356	-4.081722
46	6	0	-3.519616	1.160286	-4.200960
47	1	0	-3.244624	0.110276	-4.220214
48	6	0	-2.966876	1.987429	-3.229164
49	6	0	-2.536405	5.267295	-1.601094
50	1	0	-3.120424	6.043227	-2.090014
51	6	0	-3.290392	3.344642	-3.174255

52	6	0	-2.004988	1.597670	-2.103687
53	6	0	-1.735965	5.580776	-0.508442
54	1	0	-1.712845	6.610744	-0.161708
55	6	0	-0.957793	4.612192	0.146652
56	6	0	-1.009944	3.292296	-0.325810
57	1	0	-0.413390	2.513472	0.144152
58	6	0	-4.723833	3.049201	-5.075050
59	1	0	-5.413959	3.477945	-5.797173
60	6	0	-4.409602	1.679063	-5.148195
61	6	0	-0.079658	5.027708	1.329903
62	6	0	-4.671890	1.387736	-7.622080
63	1	0	-5.029362	2.415330	-7.747701
64	1	0	-5.122019	0.778773	-8.415395
65	1	0	-3.585548	1.387851	-7.767460
66	6	0	0.685994	3.847747	1.934075
67	1	0	0.008825	3.080392	2.326395
68	1	0	1.302557	4.203762	2.767497
69	1	0	1.353860	3.373292	1.205728
70	6	0	-0.959744	5.640506	2.432022
71	1	0	-1.503892	6.522495	2.077686
72	1	0	-0.339667	5.950289	3.282098
73	1	0	-1.695294	4.912902	2.794634
74	6	0	0.945134	6.072774	0.857794
75	1	0	1.593675	5.658032	0.077563
76	1	0	1.578330	6.386970	1.696594
77	1	0	0.457994	6.966243	0.452776
78	6	0	-6.569843	0.824967	-6.080689
79	1	0	-6.861966	0.407536	-5.110123
80	1	0	-7.036815	0.218901	-6.866528
81	1	0	-6.980839	1.837756	-6.150629
82	6	0	-4.561390	-0.637093	-6.185527
83	1	0	-3.475646	-0.713220	-6.315409
84	1	0	-5.033263	-1.209430	-6.992063
85	1	0	-4.829781	-1.118102	-5.237827

Table S7 T_1 state of **Pd-B-1**

Center Number	Atomic Number	Atomic Type	Coordinates (Angstroms) X	Y	Z
1	46	0	0.003923	0.081985	0.063956
2	7	0	-0.001911	0.046687	2.193090
3	6	0	1.912126	-0.153032	0.375884
4	8	0	2.629895	-0.458188	-1.904127
5	6	0	0.311519	0.416669	-1.869865
6	7	0	-2.071374	-0.042617	-0.263515
7	6	0	-2.720976	-0.921141	0.523017
8	1	0	-2.091200	-1.523302	1.170035
9	6	0	2.850173	-0.452538	-0.564582
10	6	0	-2.761126	0.695679	-1.145577
11	6	0	-4.162042	0.636099	-1.152735
12	1	0	-4.722050	1.268104	-1.829308
13	6	0	1.528381	0.112498	-2.505097
14	6	0	-0.998131	0.294525	3.021949
15	1	0	-1.972023	0.460563	2.568810
16	6	0	0.807684	0.998177	-4.611377

17	1	0	0.985611	1.224819	-5.659037
18	6	0	1.289175	-0.136167	2.725960
19	6	0	-0.863400	0.340988	4.421712
20	1	0	-1.730741	0.553094	5.036407
21	6	0	-4.096209	-1.062386	0.530241
22	1	0	-4.566095	-1.788922	1.184149
23	6	0	1.775355	0.363556	-3.857052
24	1	0	2.736398	0.079169	-4.276183
25	6	0	2.304369	-0.255851	1.776297
26	6	0	0.406458	0.091278	4.985841
27	1	0	0.539350	0.093162	6.063324
28	6	0	4.208430	-0.808762	-0.229108
29	1	0	4.905667	-1.042563	-1.026712
30	6	0	1.468769	-0.147052	4.151137
31	1	0	2.455733	-0.331492	4.560430
32	6	0	-4.834116	-0.235020	-0.309480
33	1	0	-5.919180	-0.277179	-0.322792
34	6	0	-0.663929	1.072703	-2.667319
35	6	0	-0.391999	1.365444	-4.012141
36	1	0	-1.122532	1.895639	-4.610509
37	6	0	4.607755	-0.808832	1.128027
38	1	0	5.640825	-1.034206	1.377159
39	6	0	3.708056	-0.522117	2.121072
40	1	0	4.026361	-0.514454	3.158245
41	6	0	-5.050656	0.796550	-6.236380
42	6	0	-2.564567	3.979368	-2.096211
43	6	0	-1.805161	2.999590	-1.440106
44	6	0	-4.173580	3.888749	-4.142543
45	1	0	-4.435431	4.943816	-4.131977
46	6	0	-3.527298	1.162486	-4.199485
47	1	0	-3.257593	0.110937	-4.203937
48	6	0	-2.969264	2.000486	-3.239938
49	6	0	-2.528061	5.299412	-1.653584
50	1	0	-3.109685	6.070981	-2.152201
51	6	0	-3.286865	3.359716	-3.203546
52	6	0	-2.007357	1.622116	-2.110406
53	6	0	-1.729056	5.623485	-0.562917
54	1	0	-1.704939	6.657108	-0.227199
55	6	0	-0.954498	4.660346	0.104403
56	6	0	-1.008320	3.335466	-0.353303
57	1	0	-0.415320	2.559958	0.126460
58	6	0	-4.723057	3.044543	-5.099254
59	1	0	-5.411486	3.466371	-5.827048
60	6	0	-4.415492	1.671990	-5.153325
61	6	0	-0.080876	5.084887	1.287675
62	6	0	-4.673344	1.344752	-7.622716
63	1	0	-5.020961	2.373652	-7.764127
64	1	0	-5.127021	0.728339	-8.408243
65	1	0	-3.586649	1.332713	-7.765117
66	6	0	0.691314	3.910930	1.895496
67	1	0	0.019784	3.137772	2.286090
68	1	0	1.303083	4.272111	2.730258
69	1	0	1.364043	3.441204	1.168625
70	6	0	-0.967595	5.695057	2.385871
71	1	0	-1.517417	6.571588	2.026623
72	1	0	-0.351796	6.012489	3.236252
73	1	0	-1.698668	4.963409	2.749373

74	6	0	0.938576	6.135127	0.815688
75	1	0	1.590642	5.723198	0.036897
76	1	0	1.568776	6.453922	1.654996
77	1	0	0.447284	7.025556	0.409032
78	6	0	-6.580055	0.820018	-6.078667
79	1	0	-6.878308	0.420362	-5.102520
80	1	0	-7.049285	0.205147	-6.856268
81	1	0	-6.983028	1.834656	-6.166124
82	6	0	-4.582711	-0.658854	-6.155062
83	1	0	-3.497420	-0.745710	-6.281928
84	1	0	-5.057839	-1.240074	-6.953291
85	1	0	-4.856425	-1.122460	-5.200241

Table S8 S_0 state of **Pd-B-2**

Center Number	Atomic Number	Atomic Type	Coordinates (Angstroms) X	Y	Z
1	46	0	0.020425	0.119809	0.074443
2	7	0	0.042121	0.151718	2.204939
3	6	0	1.952117	0.060909	0.362927
4	8	0	2.691743	-0.121647	-1.956170
5	6	0	0.299760	0.430437	-1.854477
6	7	0	-2.051945	-0.176395	-0.278349
7	6	0	-2.766026	-1.008034	0.509171
8	1	0	-2.199722	-1.527022	1.275600
9	6	0	2.930420	-0.113610	-0.611433
10	6	0	-2.683351	0.435171	-1.280511
11	6	0	-4.063508	0.320229	-1.483750
12	1	0	-4.517371	0.860994	-2.306240
13	6	0	1.519031	0.290406	-2.533590
14	6	0	-0.996079	0.342687	3.027210
15	1	0	-1.975534	0.378689	2.561359
16	6	0	0.578852	1.017753	-4.631418
17	1	0	0.686822	1.234476	-5.690235
18	6	0	1.301052	0.137576	2.713998
19	6	0	-0.858198	0.494033	4.398989
20	1	0	-1.734910	0.643456	5.019511
21	6	0	-4.126120	-1.198452	0.368903
22	1	0	-4.650447	-1.874875	1.034306
23	6	0	1.663803	0.560284	-3.899286
24	1	0	2.638463	0.421281	-4.357613
25	6	0	2.367442	-0.001808	1.715213
26	6	0	0.424561	0.451985	4.937751
27	1	0	0.580802	0.566093	6.006354
28	6	0	4.273327	-0.324396	-0.276078
29	1	0	4.999165	-0.453463	-1.074309
30	6	0	1.508308	0.278983	4.089003
31	1	0	2.516113	0.271468	4.488196
32	6	0	-4.791408	-0.499702	-0.642648
33	1	0	-5.861804	-0.614228	-0.784019
34	6	0	-0.750690	0.917023	-2.643156
35	6	0	-0.644744	1.211520	-3.998982
36	1	0	-1.512265	1.587472	-4.532229
37	6	0	4.656854	-0.368117	1.058114
38	1	0	5.699354	-0.533002	1.314766

39	6	0	3.706482	-0.208756	2.062251
40	1	0	4.014611	-0.266739	3.102142
41	8	0	-2.015119	1.230439	-2.127341

Table S9 T_1 state of **Pd-B-2**

Center Number	Atomic Number	Atomic Type	Coordinates (Angstroms) X	Y	Z
1	46	0	0.007494	0.157587	0.070241
2	7	0	0.019830	0.178490	2.181984
3	6	0	1.932223	0.099354	0.345432
4	8	0	2.695084	-0.085526	-1.948546
5	6	0	0.295276	0.433881	-1.868028
6	7	0	-2.069565	-0.133216	-0.279129
7	6	0	-2.791670	-0.939933	0.526497
8	1	0	-2.231280	-1.440065	1.309337
9	6	0	2.900691	-0.073084	-0.601951
10	6	0	-2.693654	0.451243	-1.302127
11	6	0	-4.072542	0.334101	-1.511046
12	1	0	-4.519322	0.852224	-2.351805
13	6	0	1.515301	0.281689	-2.544132
14	6	0	-0.981748	0.380096	3.015784
15	1	0	-1.967672	0.482862	2.570749
16	6	0	0.573581	0.930168	-4.665801
17	1	0	0.679984	1.111321	-5.731374
18	6	0	1.327453	0.090850	2.705549
19	6	0	-0.836828	0.457413	4.414004
20	1	0	-1.710410	0.626729	5.032976
21	6	0	-4.152020	-1.129198	0.384293
22	1	0	-4.682290	-1.784655	1.065827
23	6	0	1.660901	0.504700	-3.918139
24	1	0	2.637133	0.357193	-4.370511
25	6	0	2.341585	0.040259	1.750158
26	6	0	0.450802	0.293038	4.971415
27	1	0	0.591109	0.317970	6.047515
28	6	0	4.285209	-0.271303	-0.267440
29	1	0	5.004766	-0.412008	-1.067013
30	6	0	1.518560	0.110316	4.130924
31	1	0	2.518317	-0.005980	4.533562
32	6	0	-4.809000	-0.458258	-0.650774
33	1	0	-5.878907	-0.573342	-0.795472
34	6	0	-0.755605	0.889950	-2.673470
35	6	0	-0.650573	1.139048	-4.038786
36	1	0	-1.519555	1.494306	-4.583828
37	6	0	4.683544	-0.257989	1.091441
38	1	0	5.735132	-0.377887	1.335824
39	6	0	3.761002	-0.092814	2.089338
40	1	0	4.078886	-0.077395	3.126409
41	8	0	-2.018838	1.222510	-2.166575

Table S10 S_0 state of **Pd-N-1**

Center Number	Atomic Number	Atomic Type	Coordinates (Angstroms) X	Y	Z

1	46	0	-0.056482	0.179160	0.007854
2	7	0	-0.037780	0.468017	2.027745
3	6	0	1.878174	0.284339	-0.089919
4	7	0	0.130946	-0.076755	-1.957244
5	8	0	-2.157576	0.036790	-0.069534
6	6	0	-2.855961	-0.163896	-1.152499
7	6	0	-4.270977	-0.250420	-1.115225
8	1	0	-4.768060	-0.145213	-0.154110
9	6	0	-5.007547	-0.463870	-2.270370
10	1	0	-6.091234	-0.523950	-2.193806
11	6	0	-4.411087	-0.607107	-3.536003
12	1	0	-5.032085	-0.781002	-4.407421
13	6	0	-3.028964	-0.528746	-3.616900
14	6	0	-2.284136	-0.309807	-2.439023
15	6	0	-0.894948	-0.257646	-2.787565
16	6	0	-0.736178	-0.429506	-4.172186
17	6	0	0.543993	-0.409101	-4.688017
18	6	0	1.623625	-0.221024	-3.810980
19	1	0	2.636246	-0.202608	-4.198267
20	6	0	1.401119	-0.056952	-2.448443
21	1	0	0.731992	-0.536380	-5.751249
22	6	0	2.403463	0.143894	-1.400722
23	6	0	3.774854	0.189908	-1.652260
24	1	0	4.165287	0.079987	-2.659911
25	6	0	4.641564	0.377493	-0.584943
26	1	0	5.714337	0.416728	-0.750211
27	6	0	4.152381	0.516717	0.707690
28	1	0	4.862255	0.660212	1.512533
29	6	0	2.768663	0.472882	0.972707
30	6	0	1.048302	0.632686	2.818013
31	6	0	0.864068	0.824505	4.211518
32	1	0	1.723703	0.957893	4.851572
33	6	0	-0.397588	0.845610	4.758272
34	1	0	-0.513978	0.995258	5.827671
35	6	0	-1.507507	0.675301	3.928793
36	1	0	-2.523071	0.683359	4.307587
37	6	0	-1.273453	0.492585	2.587169
38	1	0	-2.083584	0.351859	1.879357
39	6	0	-2.096275	-0.617976	-4.831811
40	6	0	-2.285875	0.568277	-5.815555
41	6	0	-3.545483	0.625600	-6.674279
42	1	0	-1.415977	0.572506	-6.488137
43	1	0	-2.217915	1.493872	-5.226542
44	6	0	-3.606642	1.911400	-7.497293
45	1	0	-3.575583	-0.231806	-7.358847
46	1	0	-4.442655	0.563850	-6.045701
47	6	0	-4.834454	1.980661	-8.397321
48	1	0	-3.597284	2.775083	-6.817911
49	1	0	-2.696861	1.994329	-8.108722
50	1	0	-4.859072	2.913801	-8.971030
51	1	0	-4.847421	1.148877	-9.112093
52	1	0	-5.758501	1.925878	-7.809155
53	6	0	-2.097097	-2.006559	-5.531136
54	6	0	-3.432456	-2.687241	-5.826946
55	1	0	-1.513716	-2.695130	-4.904949
56	1	0	-1.528487	-1.898881	-6.466952
57	6	0	-3.240113	-3.989586	-6.601709

58	1	0	-3.947142	-2.904287	-4.882688
59	1	0	-4.099551	-2.029606	-6.396811
60	6	0	-4.551313	-4.719687	-6.866348
61	1	0	-2.739017	-3.774162	-7.556121
62	1	0	-2.559411	-4.646525	-6.041896
63	1	0	-4.387859	-5.648459	-7.424209
64	1	0	-5.055130	-4.978220	-5.927170
65	1	0	-5.239678	-4.096454	-7.450087
66	7	0	2.340021	0.621874	2.320775
67	6	0	3.388817	0.781189	3.294339
68	6	0	3.856169	2.056614	3.605683
69	6	0	3.939252	-0.344391	3.903696
70	6	0	4.880366	2.204720	4.537736
71	1	0	3.415991	2.920811	3.116134
72	6	0	4.963096	-0.190545	4.835295
73	1	0	3.562794	-1.329857	3.644188
74	6	0	5.433737	1.082619	5.152723
75	1	0	5.246136	3.197750	4.782487
76	1	0	5.393142	-1.066495	5.312326
77	1	0	6.232639	1.200523	5.879244

Table S11 T_2 state of **Pd-N-1**

Center Number	Atomic Number	Atomic Type	Coordinates (Angstroms) X	Y	Z
1	46	0	-0.065486	0.169702	0.050765
2	7	0	-0.029847	0.437147	2.068370
3	6	0	1.867050	0.272131	-0.059590
4	7	0	0.093730	-0.074968	-1.907337
5	8	0	-2.214345	0.028642	-0.065433
6	6	0	-2.891474	-0.161097	-1.135870
7	6	0	-4.318565	-0.241682	-1.108634
8	1	0	-4.818600	-0.137581	-0.150113
9	6	0	-5.031388	-0.445420	-2.265787
10	1	0	-6.115967	-0.501879	-2.211747
11	6	0	-4.411884	-0.588186	-3.537418
12	1	0	-5.032454	-0.754031	-4.410575
13	6	0	-3.039397	-0.519613	-3.618639
14	6	0	-2.291761	-0.304840	-2.431863
15	6	0	-0.910844	-0.252507	-2.751503
16	6	0	-0.758889	-0.424539	-4.155406
17	6	0	0.560509	-0.411551	-4.689466
18	6	0	1.605878	-0.232853	-3.828645
19	1	0	2.623509	-0.218352	-4.205533
20	6	0	1.397976	-0.057996	-2.398594
21	1	0	0.738990	-0.541428	-5.753276
22	6	0	2.372644	0.128407	-1.406694
23	6	0	3.773900	0.178547	-1.650808
24	1	0	4.164714	0.071277	-2.658598
25	6	0	4.633967	0.364608	-0.586876
26	1	0	5.706897	0.404280	-0.756189
27	6	0	4.156797	0.505101	0.716569
28	1	0	4.868853	0.648901	1.517959
29	6	0	2.754702	0.459330	0.985459
30	6	0	1.061069	0.607990	2.852596

31	6	0	0.886938	0.790215	4.249544
32	1	0	1.751341	0.929899	4.881941
33	6	0	-0.368926	0.792709	4.808292
34	1	0	-0.478136	0.934496	5.879449
35	6	0	-1.484778	0.612840	3.986484
36	1	0	-2.497021	0.605652	4.374132
37	6	0	-1.260256	0.442360	2.642460
38	1	0	-2.080054	0.299420	1.947951
39	6	0	-2.103813	-0.607673	-4.827373
40	6	0	-2.295368	0.582804	-5.808294
41	6	0	-3.524946	0.611720	-6.711463
42	1	0	-1.403355	0.614471	-6.450382
43	1	0	-2.270814	1.504896	-5.210852
44	6	0	-3.584405	1.894450	-7.539260
45	1	0	-3.512662	-0.247621	-7.394279
46	1	0	-4.443731	0.531962	-6.116052
47	6	0	-4.784988	1.939917	-8.476721
48	1	0	-3.611142	2.759663	-6.862358
49	1	0	-2.657837	1.991908	-8.122486
50	1	0	-4.807211	2.870638	-9.054373
51	1	0	-4.762219	1.105772	-9.188477
52	1	0	-5.725774	1.871885	-7.917130
53	6	0	-2.112140	-1.992066	-5.539532
54	6	0	-3.450766	-2.673794	-5.822493
55	1	0	-1.516968	-2.684182	-4.928921
56	1	0	-1.558489	-1.873005	-6.482637
57	6	0	-3.269296	-3.959807	-6.626410
58	1	0	-3.945066	-2.913175	-4.872437
59	1	0	-4.132007	-2.008066	-6.365927
60	6	0	-4.582286	-4.692146	-6.875583
61	1	0	-2.791192	-3.722658	-7.587336
62	1	0	-2.572480	-4.623233	-6.094925
63	1	0	-4.426089	-5.608994	-7.454743
64	1	0	-5.063524	-4.971856	-5.930633
65	1	0	-5.286882	-4.062000	-7.431876
66	7	0	2.344522	0.612189	2.339695
67	6	0	3.401711	0.793507	3.300420
68	6	0	3.852640	2.077993	3.597442
69	6	0	3.974960	-0.318521	3.913460
70	6	0	4.884244	2.249111	4.517439
71	1	0	3.394755	2.931299	3.105119
72	6	0	5.006194	-0.142008	4.832895
73	1	0	3.611065	-1.311518	3.665100
74	6	0	5.461097	1.140545	5.135317
75	1	0	5.237925	3.249490	4.749897
76	1	0	5.454869	-1.007671	5.311629
77	1	0	6.266044	1.276210	5.852046

Further details on energy transfer processes [17,18]

(1) energy transfer in [2+2] cycloaddition of styrene:

There are a number of mechanistic alternatives to the [2+2] photo-cycloaddition of styrenes, one of which involves the triplet excited state of the styrene. The lowest triplet excited state of styrene, frequently of ππ* character, is a diradical with a long lifetime in the μs regime. Thus, the radiative and non-radiative decay of the T_1 excited state of the styrene is less competitive with the photo-cycloaddition reaction and is a

more efficient reaction pathway. Population of the triplet excited state of a styrene could be brought about by direct excitation, but it can also be induced by energy transfer from another photoexcited molecule. Here, the triplet excited state of the Pd(II) complexes transfer energy to the triplet excited state of styrene *via* an electron exchange mechanism (Dexter mechanism). As this mechanism requires only a close spatial encounter of the Pd(II) complex and styrene and a favourable energy order (the triplet excited state of the styrene is lower-lying than the triplet excited state of the Pd(II) complexes) for effective triplet energy transfer, this mechanism is feasible in the present case as the triplet excited states of the Pd(II) complexes are up to 2.71 eV, which is higher-lying than the triplet excited states of the styrenes.

On the other hand, the energy transfer from the triplet excited state of the Pd(II) complexes to the singlet excited state of the styrene is not possible *via* Förster resonance energy transfer (FRET) owing to the fact that the singlet excited state of styrene is much higher-lying (~4.0 to 5.0 eV) than the triplet excited state of the Pd(II) complexes studied in this work. Energy transfer from the triplet excited state of the Pd(II) complexes to the triplet excited state of styrene by FRET is also forbidden as the $S_0 \rightarrow T_1$ absorption of styrene is spin-forbidden. Thus, the most probable mechanism for energy transfer from the Pd(II) complex to the styrene is the Dexter mechanism.

(2) energy transfer between the triplet excited state of the Pd(II) complexes and the singlet excited state of the fluorescent dyes, TBRb/TTPA, in PSF-OLED

There are two mechanism for triplet-singlet energy transfer: Förster resonance energy transfer (FRET) and Dexter mechanism. The energy transfer rates for these two mechanism are respectively

$$k_{FRET} \sim \frac{f_D f_A}{R^6 \tilde{\nu}^2} J \quad \text{and} \quad k_D \sim e^{\left(\frac{2R}{L}\right)} J$$

f_D and f_A denote the oscillator strengths of the donor (D) and the acceptor (A), R is the distance between the donor and the acceptor, and L is an effective average orbital radius of the donor and acceptor states. J is the spectral overlap between the normalized donor emission and acceptor absorption. As the phosphor-sensitized fluorescence is brought about by

$$^3D^* + {}^1A \rightarrow {}^1D + {}^1A^*$$

Dexter mechanism (also called a collision mechanism) is unlikely since it violates the Wigner-Witmer spin-conservation rule. On the contrary, for the FRET mechanism, as it is based on classical dipole-dipole interactions, this mechanism is feasible for this energy transfer process. Normally, f_D is zero for a triplet to singlet transition because it violates the spin-selection rule and $f_D = 0$ in the absence of spin-orbit coupling. Here, in the presence of a transition metal ion, Pd, heavy atom effect comes into play and lifts up the spin-selection rule such that $f_D \neq 0$. Thus, the most likely mechanism for the PSF-OLED is FRET.

References

1 G. M. Sheldrick, *Acta Crystallogr. Sect. A*, 2008, **64**, 112.

2 (*a*) W.-M. Kwok, C. Ma and D. L. Phillips, *J. Am. Chem. Soc.*, 2006, **128**, 11894; (*b*) W.-M. Kwok, C. Ma and D. L. Phillips, *J. Am. Chem. Soc.*, 2008, **130**, 5131; (*c*) P. Matousek, M. Towrie, C. Ma, W.-M. Kwok, D. L. Phillips, W. T. Toner and A. W. Parker, *J. Raman Spectrosc.*, 2001, **32**, 983; (*d*) P. Matousek, M. Towrie, A. Stanley and A. W. Parker, *Appl. Spectrosc.*, 1999, **53**, 1485.

3 C. Adamo and V. Barone, *J. Chem. Phys.*, 1999, **110**, 6158.
4 M. J. Frisch, G. W. Trucks, H. B. Schlegel, G. E. Scuseria, M. A. Robb, J. R. Cheeseman, G. Scalmani, V. Barone, B. Mennucci, G. A. Petersson, H. Nakatsuji, M. Caricato, X. Li, H. P. Hratchian, A. F. Izmaylov, J. Bloino, G. Zheng, J. L. Sonnenberg, M. Hada, M. Ehara, K. Toyota, R. Fukuda, J. Hasegawa, M. Ishida, T. Nakajima, Y. Honda, O. Kitao, H. Nakai, T. Vreven, J. A. Montgomery Jr, J. E. Peralta, F. Ogliaro, M. Bearpark, J. J. Heyd, E. Brothers, K. N. Kudin, V. N. Staroverov, R. Kobayashi, J. Normand, K. Raghavachari, A. Rendell, J. C. Burant, S. S. Iyengar, J. Tomasi, M. Cossi, N. Rega, J. M. Millam, M. Klene, J. E. Knox, J. B. Cross, V. Bakken, C. Adamo, J. Jaramillo, R. Gomperts, R. E. Stratmann, O. Yazyev, A. J. Austin, R. Cammi, C. Pomelli, J. W. Ochterski, R. L. Martin, K. Morokuma, V. G. Zakrzewski, G. A. Voth, P. Salvador, J. J. Dannenberg, S. Dapprich, A. D. Daniels, Ö. Farkas, J. B. Foresman, J. V. Ortiz, J. Cioslowski and D. J. Fox, *Gaussian 09 (Revision C.01),* Gaussian, Inc., Wallingford CT, 2009.
5 (*a*) M. M. Francl, W. J. Petro, W. J. Hehre, J. S. Binkley, M. S. Gordon, D. J. DeFree and J. A. Pople, *J. Chem. Phys.*, 1982, **77**, 3654; (*b*) P. C. Hariharan and J. A. Pople, *Theor. Chim. Acta*, 1973, **28**, 213.
6 (*a*) D. Andrae, U. Haeussermann, M. Dolg, H. Stoll and H. Preuss, *Theor. Chim. Acta*, 1990, **77**, 123; (*b*) J. M. L. Martin and A. Sundermann, *J. Chem. Phys.*, 2001, **114**, 3408.
7 M. Cossi, G. Scalmani, N. Rega and V. Barone, *J. Chem. Phys.*, 2002, **117**, 43.
8 (*a*) G. S. M. Tong, P. K. Chow, W.-P. To, W.-M. Kwok and C.-M. Che, *Chem. Eur. J.*, 2014, **20**, 6433; (*b*) G. S. M. Tong, K. T. Chan, X. Chang and C.-M. Che, *Chem. Sci.*, 2015, **6**, 3026.
9 R. Improta, V. Barone, G. Scalmani and M. J. Frisch, *J. Chem. Phys.*, 2006, **125**, 054103: 054101-054109.
10 P. K. Chow, C. Ma, W.-P. To, G. S. M. Tong, S.-L. Lai, S. C. F. Kui, W.-M. Kwok and C.-M. Che, *Angew. Chem. Int. Ed.*, 2013, **52**, 11775.
11 G. Cheng, P.-K. Chow, S. C. F. Kui, C.-C. Kwok and C.-M. Che, *Adv. Mater.*, 2013, **25**, 6765.
12 E. Turner, N. Bakken and J. Li, *Inorg. Chem.*, 2013, **52**, 7344.
13 G. Revol, T. McCallum, M. Morin, F. Gagosz and L. Barriault, *Angew. Chem. Int. Ed.*, 2013, **52**, 13342.
14 Z. Lu and T. P. Yoon, *Angew. Chem. Int. Ed.*, 2012, **51**, 10329.
15 C. O'Brien, M. Y. Wong, D. B. Cordes, A. M. Z. Slawin and E. Zysman-Colman, *Organometallics*, 2015, **34**, 13.
16 S. Thapa, A. Kafle, S. K. Gurung, A. Montoya, P. Riedel and R. Giri, *Angew. Chem. Int. Ed.*, 2015, **54**, 8236.
17 M. Klessinger, J. Michl, *Excited States and Photochemistry of Organic Molecules*, VCH Publishers, New York, 1995.
18 A. Köhler, H. Bässler, *Materials Science and Engineering R*, 2009, **66**, 71.
